# Molecular Diversity and Potential Anti-neuroinflammatory Activities of Cyathane Diterpenoids from the Basidiomycete *Cyathus africanus*

**DOI:** 10.1038/s41598-017-09118-z

**Published:** 2017-08-21

**Authors:** Jing Wei, Yuanyuan Cheng, Wan-Hui Guo, Da-Cheng Wang, Qiang Zhang, Ding Li, Jianhui Rong, Jin-Ming Gao

**Affiliations:** 1 Shaanxi Key Laboratory of Natural Products & Chemical Biology, Shaanxi Engineering Center of Bioresource Chemistry & Sustainable Utilization, College of Chemistry & Pharmacy, Northwest A&F University, Yangling, 712100 Shaanxi People’s Republic of China; 2 School of Chinese Medicine, Li Ka Shing Faculty of Medicine, University of Hong Kong, 10 Sassoon Road, Pokfulam, Hong Kong People’s Republic of China

## Abstract

Ten new polyoxygenated cyathane diterpenoids, named neocyathins A–J (**1**–**10**), together with four known diterpenes (**11**–**14**), were isolated from the liquid culture of the medicinal basidiomycete fungus *Cyathus africanus*. The structures and configurations of these new compounds were elucidated through comprehensive spectroscopic analyses including 1D NMR, 2D NMR (HSQC, HMBC, NOESY) and HRESIMS, and electronic circular dichroism (ECD) data. Neuroinflammation is implicated in the pathogenesis of various neurodegenerative diseases, such as Alzheimers’ disease (AD). All isolated compounds were evaluated for the potential anti-neuroinflammatory activities in BV2 microglia cells. Several compounds showed differential effects on the expression of inducible nitric oxide synthase (iNOS) and cyclooxygenase-2 (COX-2) in lipopolysaccharide (LPS)-stimulated and Aβ_1–42_-treated mouse microglia cell line BV-2. Molecular docking revealed that bioactive compounds (e.g., **11**) could interact with iNOS protein other than COX-2 protein. Collectively, our results suggested that this class of cyathane diterpenoids might serve as important lead compounds for drug discovery against neuroinflammation in AD.

## Introduction

Neuroinflammation is implicated in the pathogenesis of various neurodegenerative diseases including Alzheimer’s disease(AD)^1, 2^. Microglia are the resident immune cells in the central nervous system (CNS). It is well-known that microglia are often activated by different stimuli to mediate inflammatory responses in the brain, suggesting the importance of microglial inhibition against neuroinflammation and neurodegeneration^3^. Lipopolysaccharide (LPS) and amyloid-β (Aβ) could activate microglia and subsequently induce inducible oxide synthase (iNOS) and cyclooxygenase-2 (COX-2)^4, 5^. Excessive NO production directly causes neuronal injury and even neuronal death^3^. Thus, neuroinflammation may be a potential therapeutic target for treating AD and other neurodegenerative diseases.

Basidiomycetes are known to produce a broad spectrum of secondary metabolites. A number of cyathane diterpenoids with an unusual 5/6/7 tricyclic skeleton, including their xylosides, were isolated from a diverse variety of higher Basidiomycetes of the genera *Cyathus*, *Hericium*, *and Sarcodon* ^6–8^. These diterpenes were demonstrated to display a wide range of biological properties, including anti-inflammatory^9^, antimicrobial^10^ antitumor^11^ and antagonism toward the kappa-opioid receptor^12^. Several cyathane diterpenoids were found to stimulate the synthesis of NGF in human nerve cells^8, 13, 14^ indicating their potential as therapeutic agents to treat neurodegenerative ailment.

The fungi *Cyathus* is a genus in the family of Nidulariaceae, collectively known as the bird’s nest fungi. These fungi are widely distributed all over the world, and usually grow on decaying wood/woody debris and cow/horse dung. Although generally inedible, *Cyathus* species is well known as the prolific producers of bioactive cyathane diterpenoids.

We have been vigorously searching for novel bioactive compounds from basidiomycete fungi^11, 15–19^. The aim of the present study was to identify new cyathane diterpenoids against neuroinflammation. We isolated a total of 14 compounds and elucidated the structures by NMR, HRESIMS, and ECD analyses. All isolated compounds were evaluated for the anti-neuroinflammatory effects in lipopolysaccharide (LPS)- or Aβ_1–42_-stimulated mouse microglia cell line BV-2.

## Results and Discussion

### Structure elucidation

**Figure 1:**
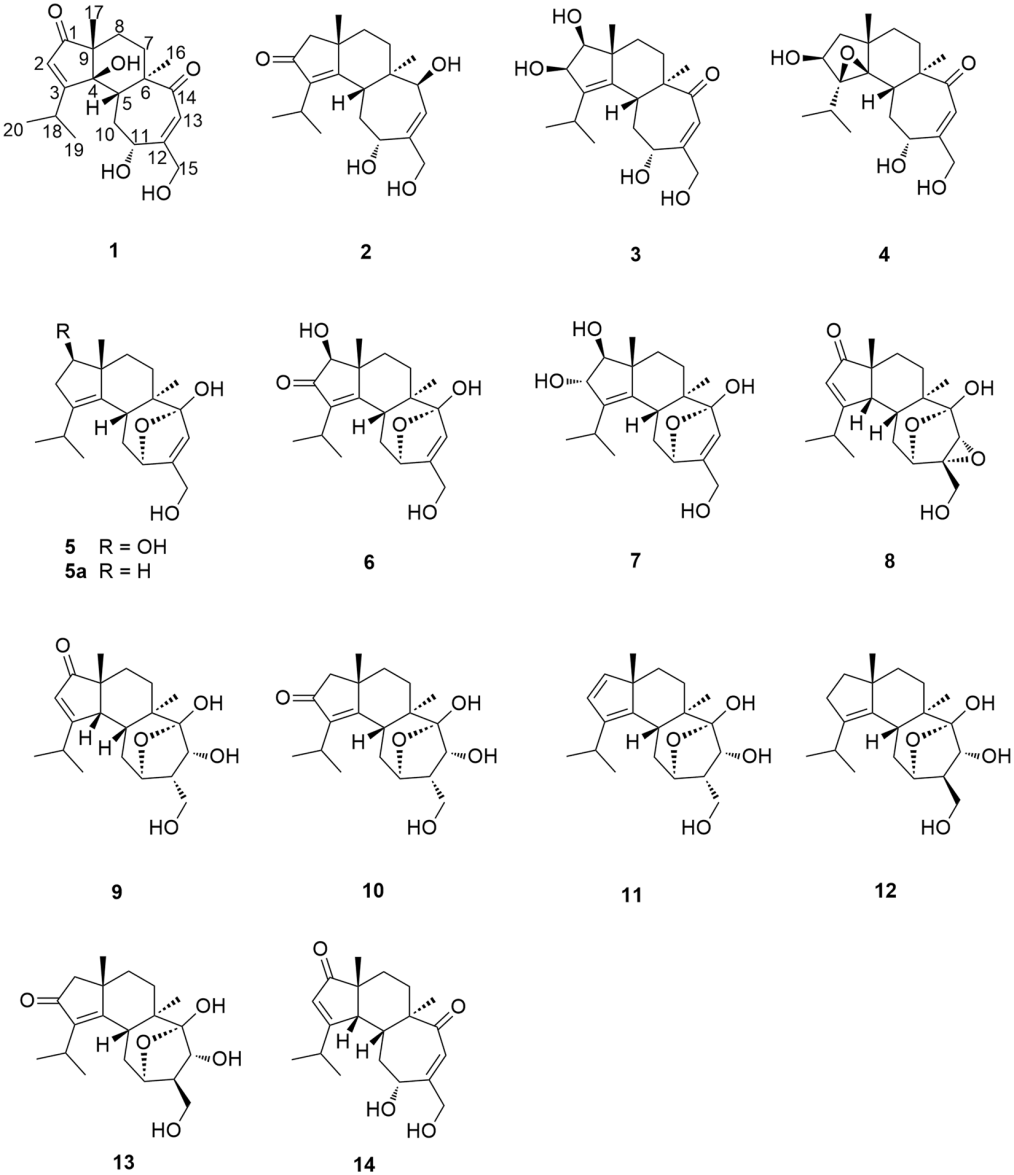
Structures of isolated compounds **1**–**14**.

The culture broth of *Cyathus africanus* was extracted with EtOAc and subjected to a series of chromatographic separations. Among a total of 14 compounds isolated, there were 10 new diterpenes, named neocyathins A–J (**1**–**10**) and four known congeners (**11**–**14**) (Fig. 1). The compounds (**11**–**14**) were identified as cyathin I (**11**)^20^ (12*R*)-11*α*,14*α*-epoxy-13*α*,14*β*,15-trihydroxycyath-3-ene (**12**)^21^ cyathin O (**13**)^22^ and allocyafrin B_4_ (**14**)^23^ respectively, by comparing their NMR data with those reported in literatures.

Compound **1** was isolated as a white powder. The molecular formula of **1** was determined to be C_20_H_28_O_5_, with seven degrees of unsaturation, on the basis of HRESIMS at *m/z* 371.1824 [M + Na]^+^. The ^1^H NMR spectrum (Table 1) revealed the presence of four methyls [δ_H_ 1.09 (3H, s, H_3_-17), 1.15 (3H, s, H_3_-16), 1.26 (3H, d, *J* = 6.7 Hz, H_3_-19), 1.37 (3H, d, *J* = 6.7 Hz, H_3_-20)], two olefinic protons [δ_H_ 5.96 (1H, s, H-2), 6.11 (1H, s, H-13)], one oxymethine [δ_H_ 4.24 (1H, m, H-11)], and two oxymethylene protons [δ_H_ 4.26 (1H, d, *J* = 15.6 Hz, H-15), 4.42 (1H, d, *J* = 15.6 Hz, H-15)]. The ^13^C NMR data (Table 2) revealed 20 carbon signals corresponding to four methyls, four methylenes (one oxygenated at δ_C_ 64.4 (C-15)), five methines [one oxygenated at δ_C_ 73.0 (C-11), two olefinic at δ_C_ 127.0 (C-2) and 122.6 (C-13)], and seven quaternary carbons [one oxygenated at δ_C_ 84.0 (C-4), two olefinic and two carbonyl).

**Table 1:** ^1^H NMR Spectroscopic Data for Compounds 1–10 in MeOH-*d*_4_.

No.	1^*a*^	2^*b*^	3^*b*^	4^*b*^	5^*b*^	6^*b*^	7^*b*^	8^*b*^	9^*b*^	10^*b*^
1		2.16 d (18.9) 2.14 d (18.9)	3.68 d (5.2)	1.50 m 1.47 m	3.79 dd (9.5 8.0)	3.75 s	3.59 d (6.9)			2.17 d (18.8) 2.16 d (18.8)
2	5.96 s		4.56 d (5.2)	4.37 m	2.44 dd (14.7 8.0) 2.09 m		4.46 d (6.9)	5.89 s	5.91 s	
4								2.79 m	2.76 dd (5.2, 1.8)	
5	2.21 d (6.3)	3.16 d (10.4)	2.72 d (7.9)	2.61 d (8.8)	2.51 m	3.03 dd (12.6, 4.1)	2.57 m	2.44 m	2.07 m	2.77 dd (12.8, 3.8)
7	1.69 m1.47 m	1.99 m 1.31 m	1.71 m 1.41 m	1.72 m 1.41 m	1.56 m 1.36 m	1.70 m 1.55 m	1.56 m 1.41 td (12.5, 4.1)	1.70 m 1.45 m	1.64 m 1.39 m	1.73 m 1.59 m
8	1.67 m 1.32 m	1.84 ddd (13.3, 4.5, 2.2) 1.53 td (13.3, 4.5)	1.55 m 1.35 m	2.27 m 1.39 m	1.66 m 1.49 td (13.5, 4.5)	1.87 m 1.75 m	1.63 m 1.49 m	1.80 m 1.38 m	2.28 m 1.45 m	1.83 m 1.66 m
10	2.87 dd (12.6, 5.3) 1.76 ddd (12.6, 10.8, 7.3)	2.21 m 1.63 m	2.52 m 1.93 m	2.34 m 1.51 m	2.16 m 1.59 m	2.38 td (12.6, 3.5) 1.64 m	2.15 td (12.6,3.4) 1.60 m	2.37 m 1.87 m	1.79 m 1.41 m	2.38 m 1.56 m
11	4.24 m	4.67 m	4.17 m	4.11 m	4.68 m	4.74 m	4.68 s	4.26 m	4.22 m	4.23 m
12									2.34 m	2.33 m
13	6.11 s	5.75 d (6.2)	6.12 s	6.12 d (1.6)	5.99 s	6.07 s	5.99	3.66 s	4.28 d (7.7)	4.49 d (7.8)
14		4.08 d (6.2)								
15	4.42 d (15.6) 4.26 d (15.6)	4.17 s	4.38 d (15.6) 4.27 d (15.6)	4.33 m 4.25 d (15.9)	4.23 2 H m	4.30 d (15.4) 4.23 d (15.4)	4.27 d (16.5) 4.21 d (16.5)	4.10 d (13.3) 3.85 d (13.3)	3.82 dd (10.9, 5.8) 3.57 dd (10.9, 8.8)	3.84 dd (10.9, 5.9) 3.60 dd (10.9, 8.7)
16	1.15 s	0.88 s	1.09	1.42 s	0.99 s	1.05 s	1.06 s	0.98 s	1.01 s	1.06 s
17	1.09 s	1.26 s	1.13	0.98 s	0.90 s	1.08 s	0.93 s	1.17 s	1.13 s	1.27 s
18	2.80 m	3.05 m	2.85 m	2.14 m	2.95 m	3.10 m	2.99 m	2.93 m	2.91 m	3.05 m
19	1.26 d (6.7)	1.21 d (6.9)	1.23 d (6.6)	1.18 d (6.9)	0.89 d (6.8)	1.18 d (6.9)	1.10 d (7.1)	1.18 d (6.5)	1.26 d (6.5)	1.17 d (7.0)
20	1.37 d (6.7)	1.20 d (6.9)	1.16 d (6.6)	1.32 d (6.9)	1.01 d (6.8)	1.17 d (6.9)	1.20 d (7.1)	1.27 d (6.5)	1.16 d (7.0)	1.20 d (7.0)

^*a*^800 MHz. ^*b*^500 MHz.

**Table 2:** ^13^C NMR Spectroscopic Data for Compounds 1–10 in MeOH-*d*_4_.

No.	1^*a*^	2^*b*^	3^*b*^	4^*b*^	5^*b*^	6^*b*^	7^*b*^	8^*b*^	9^*b*^	10^*b*^
1	213.7	53.1	88.5	47.0	82.8	84.5	90.5	215.4	216.0	53.3
2	127.0	211.1	83.5	72.8	37.2	209.5	83.3	125.8	126.4	210.9
3	188.5	144.2	140.2	77.8	137.2	141.6	139.6	192.3	193.2	144.9
4	84.0	181.7	141.4	78.2	137.8	174.0	140.1	53.3	53.5	177.0
5	43.7	43.2	39.0	37.1	41.6	43.3	41.3	38.9	37.5	40.5
6	52.9	45.8	56.6	54.6	42.6	44.9	42.8	42.7	41.9	46.0
7	33.1	32.0	34.8	34.7	31.0	30.5	30.9	29.7	29.9	29.7
8	34.5	39.3	30.2	33.7	36.2	35.3	36.1	30.3	30.8	37.1
9	54.0	43.5	48.7	42.6	49.9	47.3	47.2	50.2	50.4	42.9
10	34.8	36.6	37.0	32.3	28.3	27.6	28.2	29.9	33.3	31.5
11	73.0	71.6	72.4	72.3	80.0	79.9	79.9	74.6	76.9	76.3
12	157.5	145.9	157.0	157.3	149.0	149.2	149.2	64.0	49.7	49.8
13	122.6	127.3	123.2	123.2	126.6	126.9	126.6	57.0	70.6	70.2
14	209.7	76.2	210.6	210.1	111.2	110.8	111.1	104.8	108.2	107.4
15	64.4	65.0	64.5	64.4	58.9	58.9	58.9	59.1	62.1	62.0
16	17.4	17.1	15.6	17.9	12.0	12.2	12.2	14.6	16.1	13.2
17	14.5	24.3	23.6	20.7	17.3	22.4	19.6	20.5	22.4	26.0
18	31.2	26.7	28.2	29.8	27.5	26.3	27.2	33.0	33.0	26.2
19	22.2	21.0	24.3	20.5	21.2	20.2	19.3	23.4	21.0	20.5
20	25.3	20.1	19.6	20.4	22.6	21.2	24.6	21.0	23.3	20.6

^*a*^200 MHz. ^*b*^125 MHz.

**Figure 2:**
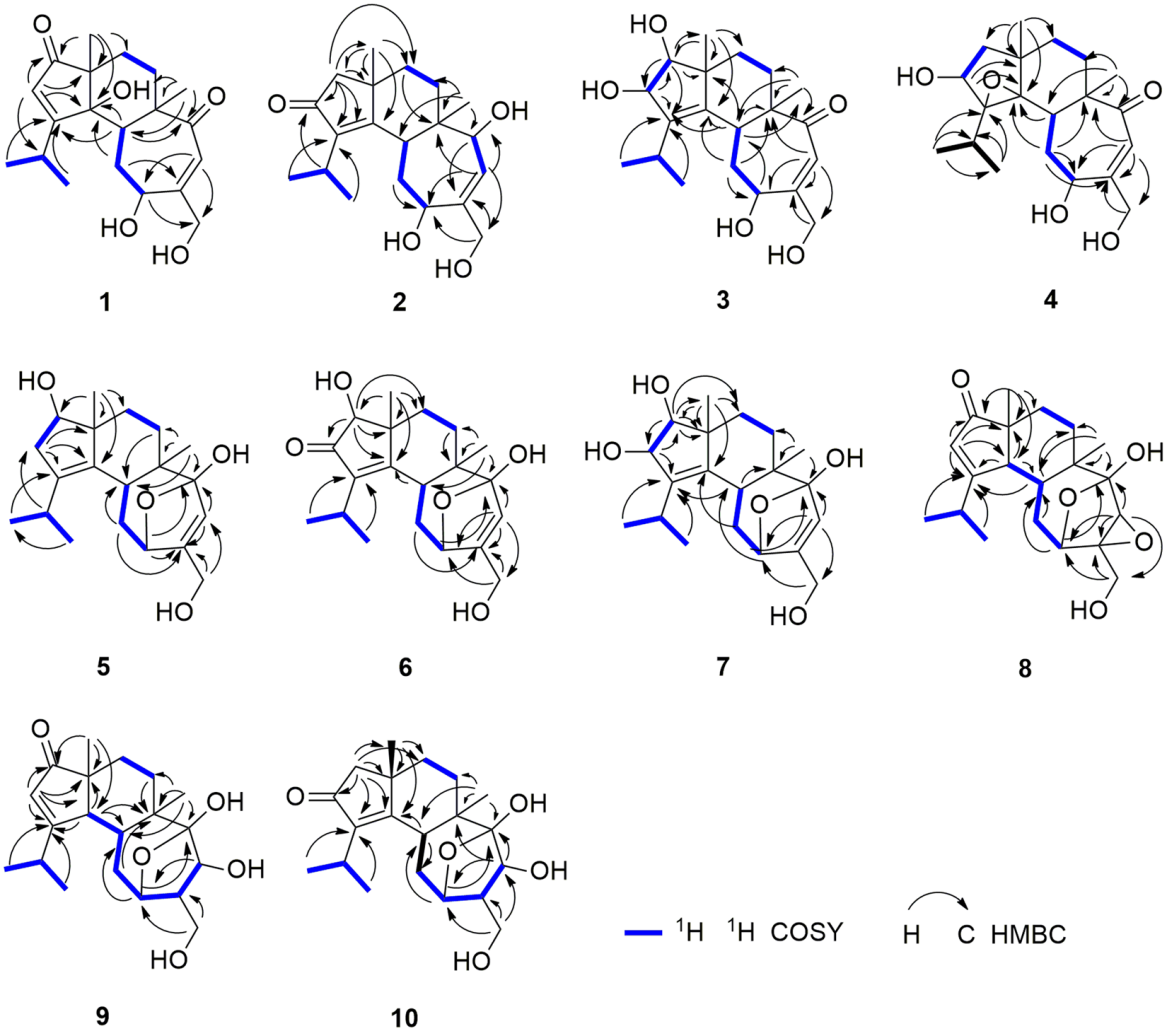
Key COSY and HMBC correlations of compounds **1**–**10**.

Compound **1** showed similar NMR spectroscopic profile to that of allocyafrin B_4_ (**14**). The key difference between compound **1** and allocyafrin B_4_ (**14**) was the replacement of the methine at C-4 (δ_C_ 56.2) by an oxygenated quaternary carbon (δ_C_ 84.0), suggesting a hydroxy group at C-4. Such prediction was supported by the HMBC correlations from H-17 to C-1, C-4, from H-2 to C-1, C-3, C-4, C-9, and from H-5, H-10 to C-4 (Fig. 2). These two compounds exhibited different chemical shifts of C-17 and C-5. The γ-effect of the hydroxy groups drove C-4 and C-17 in **1** to resonate at higher fields (14.5 ppm vs21.4 ppm), whereas the *β*-effect downshielded C-5 from 43.7 ppm to 37.8 ppm. As previously observed in cyrneine C and cyrneine D, two homologous cyathane diterpene derivatives from the fungus *Sarcodon cyrneus* ^24^ the methyl group at C-16 was upshifted by 3.6 ppm with respect to **14** (17.4 ppm vs 21.0 ppm).

**Figure 3:**
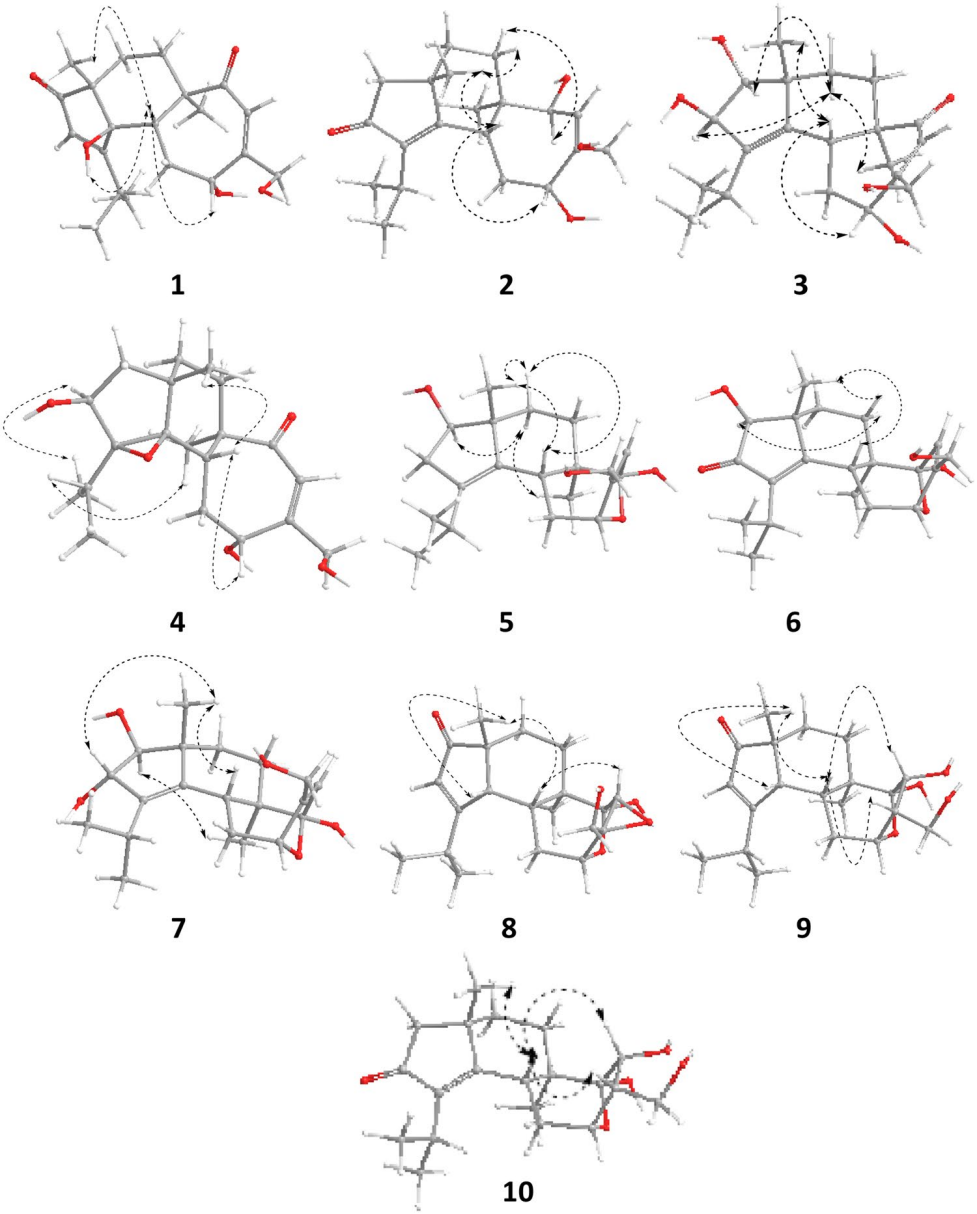
The conformation of **1**–**10** with minimized energy. The arrows show the key NOESY correlations.

The relative configuration of the hydroxy group at C-4 of **1**was determined through a NOESY experiment in DMSO-*d*_6_. The key cross-peak of H-5/4-OH in the NOESY spectrum indicated the relative *β*-configuration for 4-OH in **1** (Fig. 3). Thus, all these data allowed us to establish compound **1** as 4*β*-hydroxyallocyafrin B_4_, and named neocyathin A.

Compound **2** was isolated as a white solid. The molecular formula of **2** was determined to be C_20_H_30_O_4_ on the basis of HRESIMS at *m/z* 357.2034 [M + Na]^+^, with six degrees of unsaturation. The IR strong absorptions at 1684 cm^−1^ and UV absorption maxima at 243 nm of **2** indicated the presence of hydroxyl and *α*, *β-*unsaturated ketone functionality. The ^1^H NMR spectrum (Table 1) revealed the presence of four methyls [δ_H_ 0.88 (3H, s, H_3_-16), 1.20 (3H, d, *J* = 6.9 Hz, H_3_-20), 1.21 (3H, d, *J* = 6.9 Hz, H_3_-19), 1.26 (3H, s, H_3_-17)], one olefinic proton [δ_H_ 5.75 (1H, d, *J* = 6.2 Hz, H-13)], two oxymethines [δ_H_ 4.08 (1H, d, *J* = 6.2 Hz, H-14), 4.67 (1H, m, H-11)], and two oxymethylene protons [δ_H_ 4.17 (2H, s, H_2_-15)]. The ^13^C NMR data (Table 2) displayed 20 carbon resonances corresponding to four methyls, five methylenes [(one oxygenated at δ_C_ 65.0 (C-15)], five methines [two oxygenated at δ_C_ 71.6 (C-11) and 76.2 (C-14), one olefinic at δ_C_ 127.3 (C-13)], and six quaternary carbons [three olefinic and one carbonyl]. The NMR spectroscopic data of **2** were similar to those of cyathatriol^7^ except for the presence of a ketonic carbonyl group at C-2 (δ_C_ 211.1).

The structure of **2** was established through further analysis involving 1D NMR, HMBC and COSY spectra. The ^1^H-^1^H-COSY and HMBC spectra of **2** allowed the identification of four partial structures (Fig. 2), CH_3_-19/CH-18/CH_3_-20, CH-5/CH_2_-10/CH-11, CH-13/CH-14 and CH_2_-7/CH_2_-8. The planar structure for **2** was determined based on the key HMBC correlations from H-19 and H-20 to C-3, from H-18 to C-2, from H-17 to C-1, C-4, C-8, C-9, from H-1 to C-2, C-3, C-4, C-8, C-9, C-17, from H-16 to C-5, C-6, C-7, C-14, and from H-13 to C-6, C-11, C-14, C-15 as shown in Fig. 2.

The NOESY experiment was performed to determine the relative configuration for compound **2**. The NOESY spectrum revealed the key cross-peaks of H-5/H-11, H-5/17-Me, H-7a/17-Me and H-7b/H-14, indicating the 5R*, 6R*, 9S*, 11R*, 14S* configuration for compound **2** (Fig. 3).

**Figure 4:**
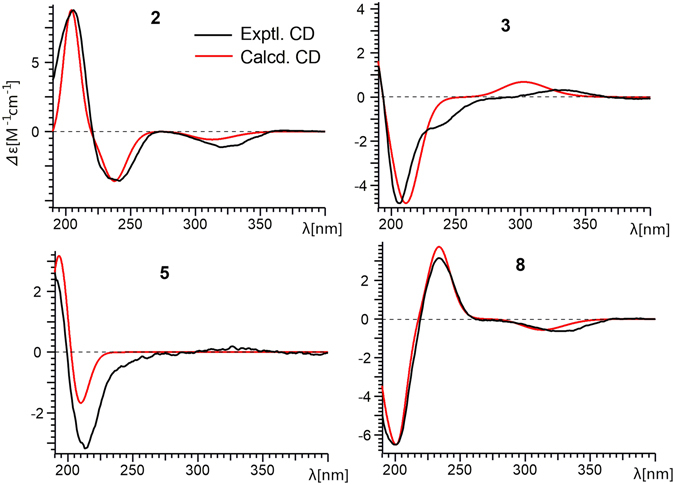
Experimental and theoretical ECD spectra of **2**, **3**, **5** and **8**.

To confirm the absolute configuration of **2**, we measured its electronic CD spectrum in acetonitrile (Fig. 4). The experimental curve of **2** showed the positive Cotton effects (CEs) at 206 nm (*Δε* + 43.4) and the negative CEs at 242 (*Δε* − 17.7) and 320 nm (*Δε* − 5.61). Consistently, the time-dependent density functional theory (TDDFT) method (cam-B3LYP/TZVP) provided similar calculated ECD values, thereby implying its absolute configuration to be 5*R*, 6*R*, 9*S*, 11*R*, 14*S*. Accordingly, the structure of **2** was established as shown, and named neocyathin B.

Compound **3** was isolated as a yellow oil. The molecular formula of **3** was determined to be C_20_H_30_O_5_ based on the HRESIMS at m/z 373.1980 [M + Na]^+^. The ^13^C NMR data (Table 2) revealed 20 carbon resonances, corresponding to four methyls (two singlets and two doublets), four methylenes (one oxygenated), six methines (three oxygenated and one olefinic), and six quaternary carbons (three olefinic and one carbonyl). The NMR spectroscopic data of **3** were similar to those of allocyafrin B_4_ (**14**). The main differences between **3** and **14** were the presence of two hydroxy groups at C-1 (δ_C_ 88.5) and C-2 (δ_C_ 83.5) and an olefinic bond at C-3 (δ_C_ 140.2) and C-4 (δ_C_ 141.4) in **3**. These observations were supported by the HMBC correlations from H-17 to C-1, C-4, from H-19 and H-20 to C-3, from H-10 to C-4, from H-5 and H-10 to C-4, C-3, from H-1 to C-2, C-3, and from H-2 to C-3 (Fig. 2). Further analysis of the HMBC and COSY spectra allowed the structural establishment of **3**. From the COSY spectrum, a CH-5/CH_2_-10/CH-11 and CH_2_-7/CH_2_-8 were established. The key HMBC correlations from H-17 to C-1, C-4, C-8, C-9, from H-1 to C-2, C-3, C-8, C-9, C-17, from H-16 to C-5, C-6, C-7, C-14, and from H-13 to C-6, C-11, C-12, C-15 (Fig. 2). Hence, the planar structure of **3** was elucidated as shown.

The relative configuration of **3** was determined through a NOESY experiment. The key cross-peaks of H-5/H-11, H-5/17-Me, H-8a/16-Me, H-8a/H-1 and H-8a/H-2 in the NOESY spectrum indicated the relative configuration of **3** was (1S*, 2R*, 5R*, 6R*, 9R*, 11R*) (Fig. 3). The experimental ECD spectrum of compound **3** matched well with calculated ECD spectrum of (1*S*, 2*R*, 5*R*, 6*R*, 9*R*, 11*R*)-**3** (Fig. 4). Thus, the structure and absolute configuration of **3** were established as shown, and named neocyathin C.

Compound **4** was isolated as a white powder. The molecular formula of **4** was determined to be C_20_H_30_O_5_, with six degrees of unsaturation, based on the HRESIMS at *m/z* 373.1979 [M + Na]^+^. The ^1^H NMR spectrum (Table 1) revealed the presence of four methyls [δ_H_ 0.98 (3H, s, H_3_-17), 1.18 (3H, d, *J* = 6.9 Hz, H_3_-19), 1.32 (3H, d, *J* = 6.9 Hz, H_3_-20), 1.42 (3H, s, H_3_-16)], one olefinic proton [δ_H_ 6.12 (1H, d, *J* = 1.6 Hz, H-13)], two oxymethines [δ_H_ 4.11 (1H, m, H-11), 4.37 (1H, m, H-2)], and two oxymethylene protons [δ_H_ 4.25 (1H, d, *J* = 15.9 Hz, H-15), 4.33 (1H, m, H-15)]. The ^13^C NMR data (Table 2) revealed 20 carbon resonances, corresponding to four methyls (two singlets and two doublets), five methylenes (one oxygenated), five methines (two oxygenated at δ_C_ 72.3 and 72.8 and one olefinic at δ_C_ 123.2), and six quaternary carbons (two oxygenated at δ_C_ 77.8 and 78.2, one olefinic δ_C_ 157.3, and one carbonyl at δ_C_ 210.1). The ^1^H-^1^H COSY spectrum of **4** showed the spin system of CH_3_-19/CH-18/CH_3_-20, CH_2_-1/CH-2, CH-5/CH_2_-10/CH-11 and CH_2_-7/CH_2_-8. In the HMBC spectrum of **4**, the HMBC correlations from H-2 to C-4, C-9, from H-5 to C-4, from H-10 to C-4, C-6, C-11 and C-12, from H-13 to C-6, C-11, C-12 and C-15, from H_3_-16 to C-5, C-6, C-7, and C-14, from H_3_-17 to C-1, C-4, C-8, and C-9, and from H_3_-19(20) to C-3, C-18 completed the structure of the cyathane diterpenoid skeleton (Fig. 2). The chemical shift for C-3 (δ_C_ 77.8) and C-4 (δ_C_ 78.2), together with the degree of unsaturation requirement, suggested the epoxy bridge between C-3 and C-4. Detailed analysis of the 1D NMR together with the HMBC and COSY data revealed the main differences between compounds **4** and **3** were the presence of an additional epoxy bridge between C-3 and C-4 instead of a double bond, and the absence of the hydroxy group at C-1 (δ_C_ 47.0) in **4**. This deduction was supported by the HMBC correlations from H-17 to C-1, C-4, from H-19 and H-20 to C-3, from H-5 and H-10 to C-4, and from H-2 to C-4, C-9 (Fig. 2).

The relative configurations of the epoxide group and the hydroxy group at C-2 of **4** were assigned by NOESY experiments. The cross-peaks of H-2/19-Me and H-18/16-Me in the NOESY spectrum confirmed all *β* orientation of the epoxy bridge and the hydroxy group at C-2 (Fig. 3). Thus, the structure of **4** was established as shown, and named neocyathin D.

Compound **5** was isolated as a white powder. The molecular formula of **5** was determined to be C_20_H_30_O_4_, with six degrees of unsaturation, based on the HRESIMS at m/z 357.2032 [M + Na]^+^. The ^13^C NMR data (Table 2) revealed 20 carbon resonances, inculding four methyls, five methylenes (one oxygenated), five methines (two oxygenated and one olefinic at δ_C_ 126.6), and six quaternary carbons (three olefinic at δ_C_ 137.2, 137.8, 149.0 and a ketal carbon at δ_C_ 111.2). The chemical shifts for C-11 (δ_C_ 80.0) and C-14 (δ_C_ 111.2) together with the degree of unsaturation requirement supported the existence of an epoxy bridge between C-11 and C-14, which was implied by HMBC correlation of H-11 (δ_H_ 4.68) with C-14.

The ^1^H-^1^H-COSY and HMBC spectra of **5** allowed the identification of four partial structures, CH_3_-19/CH-18/CH_3_-20, CH-1/CH_2_-2, CH-5/CH_2_-10/CH-11 and CH_2_-7/CH_2_-8 (Fig. 2). The connectivity of each partial structure was clarified by the HMBC spectrum as shown in Fig. 2. The key HMBC correlations observed from H-20 to C-3, from H-18 to C-2, from H-17 to C-1, C-4, C-8, C-9, from H-2 to C-3, C-4, C-9, from H-16 to C-5, C-6, C-7, C-14, from H-10 to C-5, C-12, from H-11 and H-13 to C-12, C-14, and from H-15 to C-12, C-13 indicated that the planar cyathane structure of **5** was elucidated as shown.

The relative configuration of **5** was determined by NOESY experiments. The key NOE correlations of H-5/17-Me, H-8a/17-Me and H-8b/H-1 in the NOESY spectrum indicated the hydroxy group at C-1 of **5** was *β*-oriented (Fig. 3). The *α* stereochemistry of the epoxy bridge at C-11 and C-14 could not be clarified by the NOESY data. Compound **5** had a similar structure to the tautomeric internal hemiacetal **5a** of cyathin A_3_, a metabolite of the bird’s nest fungus *Cyathus helenae* Brodie, except for the presence of the OH group at C-1 (δ_C_ 82.8), whose absolute configuration had been unambiguously determined^25, 26^. Furthermore, the experimental ECD spectrum of compound **5** matched well with calculated ECD spectrum of (1*R*, 5*R*, 6*R*, 9*R*, 11*R*, 14*R*)-**5** (Fig. 4). Thus, the structure including absolute configuration of **5** was established as shown, and named neocyathin E.

The molecular formula of **6** was determined to be C_20_H_28_O_5_, with seven degrees of unsaturation, based on the HRESIMS at *m/z* 371.1825 [M + Na]^+^. The IR and UV spectra showed the presence of one *α*, *β-*unsaturated ketone moiety (IR *ν* 1696 cm^−1^; UV *λ*_max_ 243 nm). The NMR spectroscopic data of **6** resembled those of **5** except for the presence of a ketonic carbonyl group at C-2 (δ_C_ 209.5), suggesting the *α*,*β-*unsaturated ketone unit [(δ_C_ 209.5, 174.0 (C-4), 141.6 (C-3)] present in the molecule. This was supported by HMBC correlations of H-1 to C-2 and C-4, H-19 and H-20 to C-3, and H-5 and H-17 to C-4 (Fig. 2). The unambiguous assignments of the signals in the ^1^H and ^13^C NMR spectra were based on the 2D-NMR (HMBC, HSQC, and COSY) experiments. The *β-*configuration of the hydroxy group at C-1 of **6** was determined by NOESY cross-peaks of H-1/H-7a, H-5/H-17, and H-7b/H-17 (Fig. 3). Thus, the structure of **6** was established as shown, and named neocyathin F.

The molecular formula of **7** was determined to be C_20_H_30_O_5_, with six degrees of unsaturation, based on the HRESIMS at m/z 373.1982 [M + Na]^+^. The ^13^C NMR/HSQC spectra (Table 1) of compound **1** indicated the presence of 20 carbon atoms and also revealed its close structural similarity to **5**. Detailed analysis of the 1D NMR together with the HMBC and COSY data revealed the small differences between **7** and **5** were an additional hydroxy group at C-2 (δ_C_ 83.3), which were supported by the HMBC correlations from H-1 (δ_H_ 3.59) to C-2, and from H-2 (δ_H_ 4.46) to C-1 (δ_C_ 90.5), C-3 (δ_C_ 139.6) (Fig. 2).

The relative configuration of the hydroxy group at C-2 of **7** was determined through a NOESY experiment, in which the cross-peak of H-2/Me-17 indicated the OH group with *α* orientation (Fig. 3). The assignments of all proton and carbon signals were fully made by 2D (^1^H-^1^H COSY, HSQC, and HMBC) NMR data. Thus, the structure of **7** was established as shown, and named neocyathin G.

The molecular formula of **8** was determined to be C_20_H_28_O_5_, based on the HRESIMS at m/z 371.1825 [M + Na]^+^, with seven degrees of unsaturation. The ^1^H NMR spectrum (Table 1) revealed the presence of four methyls [δ_H_ 0.98 (3H, s, H_3_-16), 1.17(3H, s, H_3_-17)], 1.18 (3H, d, *J* = 6.5 Hz, H_3_-19), 1.27 (3H, d, *J* = 6.5 Hz, H_3_-20), one olefinic proton [δ_H_ 5.89 (1H, s, H-2)], two oxymethines [δ_H_ 3.66 (1H, s, H-13), 4.26 (1H, m, H-11)], and two oxymethylene protons [δ_H_ 3.85 (1H, d, *J* = 13.3 Hz, H-15), 4.10 (1H, d, *J* = 13.3 Hz, H-15)]. A comparison of the ^13^C NMR data between **8** and **14** (Table 2) suggested the presence of one *α*,*β-*unsaturated ketone moiety (δ_C_ 125.8, 192.3, and 215.4; IR 1689 cm^−1^; UV 237 nm) in the molecule. This was supported by the HMBC correlations of H-17/C-1, C-4, H-2/C-1, C-3, C-4, and C-9 (Fig. 2). Further comparison of the ^13^C NMR data between **8** and **7** (Table 2) disclosed that the olefinic bond at C-12 (δ_C_ 149.2) and C-13 (δ_C_ 126.6) in **7** were replaced by an epoxy group at C-12 (δ_C_ 64.0) and C-13 (δ_C_ 57.0) in **8**. This deduction was confirmed by the HMBC correlations of H-13/C-12, C-14, and H-15/C-12 (Fig. 2). The unambiguous assignments of the signals in the ^1^H and ^13^C NMR spectra were based on the 2D-NMR (HMBC, HSQC, and COSY) experiments (Fig. 2). Hence, the planar structure of **8** was elucidated as shown. Compound **8** was an analogue of cyafrin A_5_ with the epoxy group at C-12 and C-13, a metabolite of *Cyathus africanus* ^27^.

The relative configuration of **8** was determined through a NOESY experiment. The key cross-peaks of H-5/17-Me, H-5/H-13 and H-4/17-Me in the NOESY spectrum indicated that the epoxy group at C-12 and C-13 was *α*-oriented and that the relative configuration of **8** was (4R*, 5R*, 6R*, 9R*, 11R*, 12R*, 13R*, 14S*) (Fig. 3). Furthermore, the experimental ECD spectrum of **8** matched well with calculated ECD spectrum of (4*R*, 5*R*, 6*R*, 9*R*, 11*R*, 12*R*, 13*R*, 14*S*)-**8** (Fig. 4). Thus, the structure and absolute configuration of **8** was therefore determined as shown, and named neocyathin H.

The molecular formula of **9** was determined to be C_20_H_30_O_5_ based on the HRESIMS at *m/z* 373.1985 [M + Na]^+^, with six degrees of unsaturation. The ^13^C NMR and HSQC spectra (Table 2) of **9** indicated the presence of 20 carbon atoms and also revealed its close structural similarity to **8**. The major differences included the absence of an epoxide ring group between C-12 and C-13, and the appearance of an additional hydroxy group at C-13 (δ_C_ 70.6) in **9**. The location of the OH group was supported by the HMBC correlations from H-15 (δ_H_ 3.82, 3.57) to C-11 (δ_C_ 76.9), C-12 (δ_C_ 49.7), and from H-13 (δ_H_ 4.28) to C-11 (Fig. 2). The configuration of the hydroxymethyl at C-12 and the hydroxy group at C-13 (δ_C_ 70.6) in **9** was both assigned as *α-*form by NOESY experiments, in which the key correlations of H-5/H-12 and H-5/H-13 (Fig. 3). This deduction suggested that **9** had the same relative configuration as that of cyathin I (**11**). Thus, the structure of **9** was established as shown, and named neocyathin I.

The molecular formula of **10** was determined to be C_20_H_30_O_5_ on the basis of the HRESIMS at *m/z* 373.1985 [M + Na]^+^, identical to that of cyathin O (**13**)^21^. The ^13^C NMR data of **10** (Table 2) were very similar to those of **13** (Table S2). Detailed analysis of the 1D NMR together with the HMBC and COSY data revealed the main differences in chemical shifts of C-10 to C-15 between **10** and **13** were attributed to the *α-*configuration of a hydroxymethyl group at C-12 (δ_C_ 49.8) in **10** instead of the *β-*orientation of the corresponding hydroxymethyl at C-12 (δ_C_ 55.8) in **13** (Table S1, Supporting Information). The *α-*configuration was confirmed by NOESY experiments, in which the key cross-peaks of H-5*β* (δ_H_ 2.77)/H-12 (δ_H_ 2.33) and H-5*β*/H-13 (δ_H_ 4.49) (Fig. 3). In addition, the chemical shifts of C-10 to C-15 in **10** closely resembled those of cyathin I (**11**) (Table S1, Supporting Information). Thus, the structure of **10** was established as shown, and named neocyathin J.

### Evaluation of the isolated compounds for regulating the expression and activity of iNOS and COX-2 in LPS-stimulated or Aβ_1–42_-treated BV2 cells

Microglial cells are inflammatory cells in the regulation of neurodegeneration^28^. To explore the biological activities of these cyathane diterpenoids, we firstly examined the cytotoxicity of the isolated compounds in BV2 microglial cells. As shown in Figure S63 (Supporting Information), all compounds at the concentrations from 5 μM to 50 μM for 24 h did not show any toxicity. Following incubation of all compounds for 72 h, most of compounds exhibited no toxicity under indicated concentrations, except for **4** and **6** at 50 μM with somewhat toxicity.

**Figure 5:**
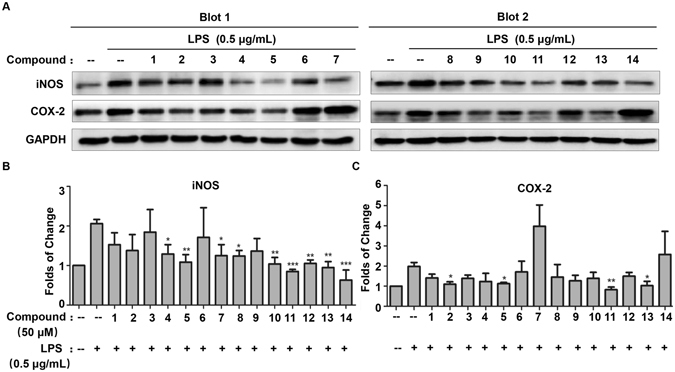
Effects of isolated compounds on LPS-induced iNOS and COX-2 expression in BV2 microglia cells. (**A**) Western blot analysis of iNOS and COX-2 expression. The cells were pre-treated with all compounds with the concentration of 50 μM for 1 h, and subsequently incubated with 0.5 μg/mL of LPS for 24 h. The expression of iNOS and COX-2 was detected by Western blotting and quantified by Image Lab software. (**B** and **C**) Quantification of iNOS and COX-2 induction (Panel A). Data were expressed as mean ± SD (n = 3) and analyzed by one-way ANOVA with Dunnett’s test for iNOS expression and LSD test for COX-2 expression. *p < 0.05; **p < 0.01; ***p < 0.001. (Drug plus LPS vs LPS).

**Figure 6:**
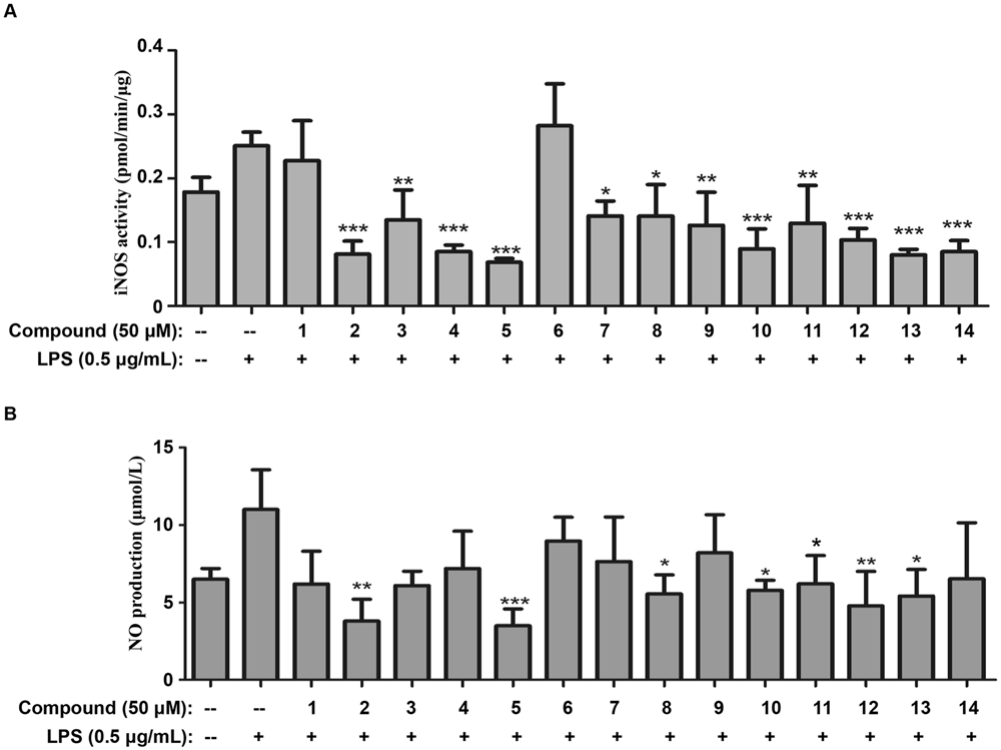
Effects of isolated compounds on iNOS enzyme activities and NO production in LPS-stimulated BV2 microglia cells. (**A**) Assays of iNOS enzyme activities. BV2 cells were pre-treated with all compounds (50 μM) for 1 h, and stimulated with 0.5 μg/mL LPS for 24 h. The activities of iNOS enzyme were determined by using nitric oxide synthase activity assay kit (Abcam). The results were presented as mean ± SD (n = 3) and analyzed by one-way ANOVA with Dunnett’s test. *p < 0.05; **p < 0.01; ***p < 0.001. (Drug treatment plus LPS vs LPS). (**B**) Determination of NO levels in culture medium. After the drug treatment as previously described, the culture medium was collected for measurement of NO production by using Griess reagent kit for nitrite determination. Data were expressed as mean ± SD (n = 3) and analyzed by one-way ANOVA with Dunnett’s test. *p < 0.05; **p < 0.01; ***p < 0.001. (Drug plus LPS vs LPS).

COX-2 and iNOS are two major inflammatory mediators in brain neurodegeneration^29, 30^. We subsequently examined the effects of all isolated compounds at the concentration of 50 μM on the expression of iNOS and COX-2 in LPS-stimulated BV2 microglial cells. As shown in Fig. 5, Western blot analysis demonstrated that compounds **2**, **5**, **11** and **13** significantly suppressed LPS-induced COX-2 expression, whereas compounds **4**, **5**, **7**, **8**, **10**, **11**, **12**, **13** and **14** markedly inhibited LPS-induced iNOS expression. Among these compounds, **5**, **11** and **13** showed strong inhibitory effects on both COX-2 and iNOS. Interestingly, 7, **12** and **14** abolished LPS-induced iNOS expression, but did not affect LPS-induced COX-2 expression. In addition, we also assayed the activities of iNOS enzyme. As shown in Fig. 6A, compounds **2**, **3**, **4**, **5**, **7**, **8**, **9**, **10**, **11**, **12**, **13 and 14** dramatically attenuated LPS-induced iNOS enzyme activities. The enzyme iNOS catalyzes the generation of nitric oxide (NO) from L-arginine oxidized upon stimulation^31^. Indeed, compounds **2**, **5**, **8**, **12 and 13** significantly suppressed LPS-induced NO production in culture medium.

**Figure 7:**
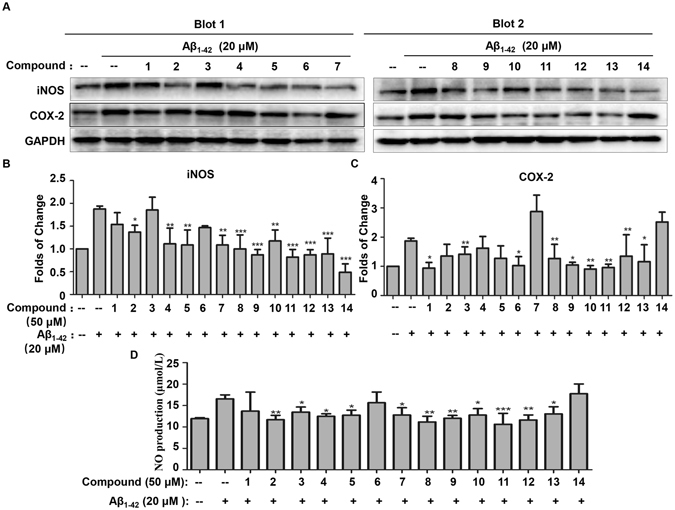
Effects of isolated compounds on iNOS, COX-2 expression and NO production in Aβ_1–42_-treated BV2 microglia cells. (**A**) Western blot analysis of iNOS and COX-2 expression. The cells were pre-treated with all compounds (50 μM) for 1 h, and subsequently incubated with 20 μM of Aβ_1–42_ for 24 h. The expression of iNOS and COX-2 was detected by Western blotting and quantified by Image Lab software. (**B** and **C**) Quantification of iNOS and COX-2 induction (Panel A). Data were expressed as mean ± SD (n = 3) and analyzed by one-way ANOVA with Dunnett’s test for iNOS expression and LSD test for COX-2 expression. *p < 0.05; **p < 0.01; ***p < 0.001. (Drug plus Aβ_1–42_ vs Aβ_1–42_). (**D**) Determination of NO production in Aβ_1–42_-treated BV2 microglia cells. BV2 cells were pretreated with all compounds (50 μM) for 1 h, and then stimulated with Aβ_1–42_ (20 μM) for 6 h, the culture medium was collected for measurement of NO production by using Griess reagent kit for nitrite determination. Data were expressed as mean ± SD (n = 3) and analyzed by one-way ANOVA with LSD test. *p < 0.05; **p < 0.01; ***p < 0.001. (Drug plus Aβ_1–42_ vs Aβ_1–42_).

Neurotoxic peptide Aβ is well-known to induce iNOS and COX-2 expression and stimulate NO production in microglial cells^4^. Thus, we investigated all compounds for the anti-inflammatory effects on not only iNOS, COX-2 expression but also NO production in Aβ_1–42_-treated BV2 cells. As shown in Fig. 7, compounds **1**, **3**, **6**, **8**, **9**, **10**, **11**, **12** and **13** inhibited COX-2 expression whereas compounds **2**, **4**, **5**, **7**, **8**, **9**, **10**, **11**, **12**, **13** and **14** significantly suppressed iNOS expression in Aβ_1–42_-stimulated BV2 cells. In addition, we examined the effects of all compounds on the production of NO. Consistent with previous study, compounds **2**, **3**, **4**, **5**, **7**, **8**, **9**, **11**, **12** and **13** markedly suppressed Aβ_1–42_-induced NO production. These results indicated that the bioactive compounds might exhibit anti-neuroinflammatory activities through inhibiting the expression and activity of pro-inflammatory enzymes (e.g., iNOS and COX-2).

**Figure 8:**
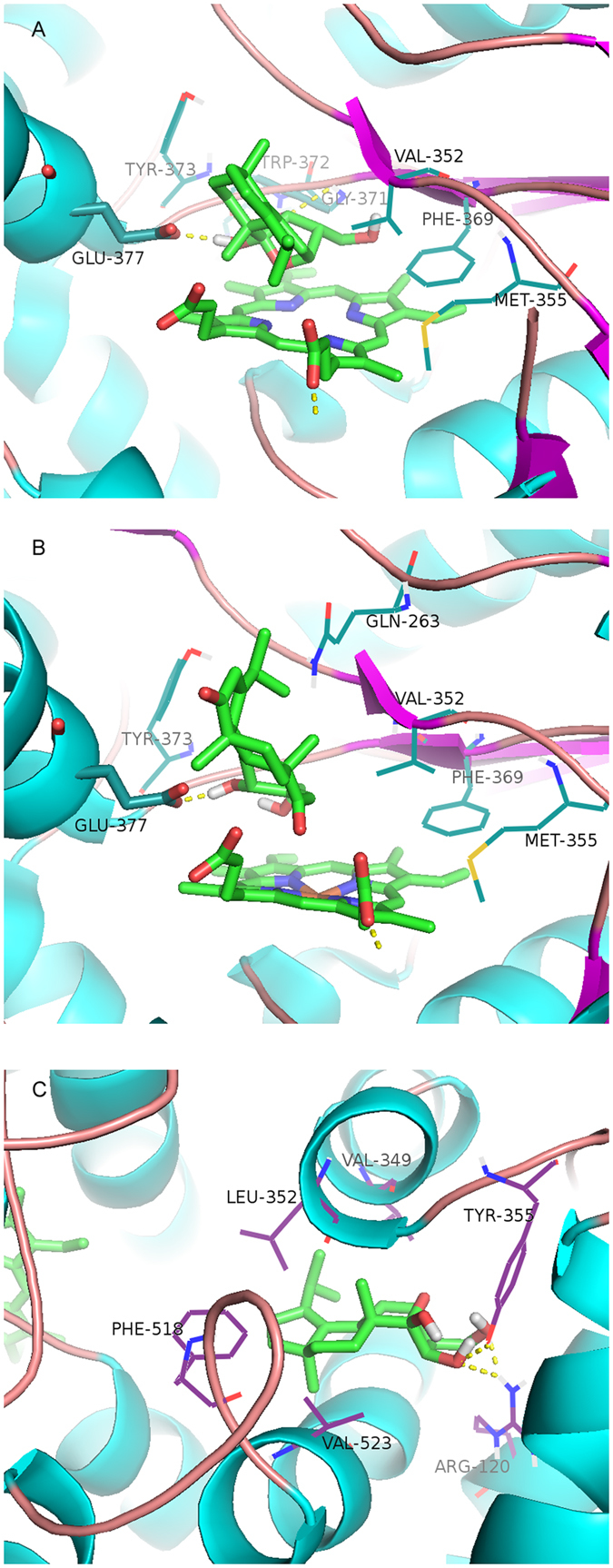
Molecular docking simulations of the binding of compounds 11 and 14 to iNOS and COX-2. Virtual drug-protein complexes including compounds **11** (**A**) and **14** (**B**) for iNOS, and **11** (**C**) for COX-2 were obtained at lowest energy conformation, while potential hydrogen contacts between compounds and proteins were highlighted by dashed lines. Atoms were colored as follows: carbon, cyan; nitrogen, blue; oxygen, red; hydrogen, gray; sulfur, orange.

To further elucidate the inhibitory effects of the bioactive diterpenoids on iNOS and COX-2, we performed molecular docking to understand the interaction of the active compounds **11** and **14** with iNOS and COX-2 proteins as previously described^32^. Surprisingly, molecular docking studies revealed strong interactions between compounds **11** and **14** with iNOS protein (Fig. 8). Consistently, compounds **11** and **14** strongly inhibited iNOS enzyme activity. On the other hand, we also discovered that compound **11** could strongly interact with the COX-2 protein. The logarithm of free binding energy and the binding residues were summarized in Table 3. Based on the results of molecular docking and activity assays, we postulated that this class of bioactive terpenoids (e.g., compound **11**) might exhibit anti-inflammatory activities by binding to the active cavities of iNOS and COX-2.

**Table 3:** Logarithms of Free Binding Energies (FBE, kcal/mol) of the Inhibitors to the Active Cavities of iNOS (PDBcode: 3E7G) and COX-2 (PDB code: 1CX2), and Targeting Residues with Hydrogen Bonding to the Inhibitors in the Binding Site Located on the Mobile Flap.

compound	iNOS		COX-2	
	log(FBE)	targeting residues	log(FBE)	targeting residues
**11**	−7.84	GLY371, TRP372,GLU377	−7.82	ARG120, TYR355
**14**	−8.24	GLU377	−9.31	ARG120

## Conclusion

In this study, we have isolated and fully characterized through extensive NMR spectroscopic analysis ten new cyathane-type diterpenoids (**1–10**) from the liquid culture of *C*. *africanus*. Interestingly, our results indicated that bioactive compounds might have anti-neuroinflammatory activities through the inhibition of pro-inflammatory enzymes expression and activities. Especially, compounds **11** and **14** strongly interact with the iNOS and COX-2 proteins by targeting the residues near the active centers of iNOS and COX-2. Thus, we anticipate that the bioactive components in *C*. *africanus* may be utilized for the development of anti-inflammatory agents against AD and related neurodegenerative disorders.

## Methods

### General experimental procedures

Optical rotations were recorded on an Autopol III automatic polarimeter (Rudolph Research Analytical). UV and IR spectra were obtained on a Thermo Scientific Evolution-300 UV–visible spectrophotometer and a Bruker Tensor 27 FT-IR spectrometer with KBr pellets. ECD spectra were obtained on a Chirascan spectrometer. NMR spectra were obtained on Bruker Avance 800 MHz and Bruker Avance III 500 spectrometers with tetramethylsilane as an internal standard at room temperature. High-resolution (HR) ESIMS were recorded on a Thermo Fisher Scientific Q-TOF mass spectrometer. Electrospray ionization mass spectrometry ESIMS were recorded on a Thermo Fisher LTQ Fleet instrument spectrometer. Silica gel (300–400 mesh, Qingdao Marine Chemical Ltd., People’s Republic of China) and RP- 18 gel (20–45 μm, Fuji Silysia Chemical Ltd., Japan) were used for column chromatography (CC). Semipreparative HPLC was performed on a Waters 1525EF liquid chromatography system equipped with a Hypersil BDS C18 column (4.6 mm × 250 mm; 10.0 mm × 250 mm). Fractions were monitored by TLC. Compounds were visualized by heating silica gel plates immersed in 10% H_2_SO_4_ in ethanol.

### Fungal material

The fungus *Cyathus africanus* was purchased from China General Microbiological Culture Collection Center (CGMCC) with the accession number CGMCC 5.1163. A specimen (No. CA20150728) was deposited at the College of Science, Northwest A&F University, Shaanxi, China. The culture medium consisted of glucose 2%, yeast extract 0.2%, peptone 0.5%, MgSO_4_ 0.05%, KH_2_PO_4_ 0.1%, with pH 6.5. Fermentation was carried out on a shaker at 130 rpm for 28 days at 28 °C.

### Extraction and isolation

The culture broth (40 L) was filtered, and the filtrate was concentrated to 5 L, then extracted with ethyl acetate (5 L × 3), while the mycelium was extracted three times with CHCl_3_–MeOH (1:1). The EtOAc layer together with the mycelium extract was concentrated under reduced pressure to give a crude extract (30.2 g), and the latter was applied to a RP-18 column eluted with a gradient of MeOH–H_2_O (10–100%) to obtain six fractions, A–F. Fraction E was further purified by silica gel CC eluting with a gradient of CHCl_3_–MeOH (50:1–10:1), to yield five fractions (E1–E5). Fraction E2 was subjected to Sephadex LH-20 (MeOH) and further purified by semi-preparative HPLC (55%, MeOH–H_2_O, 2 mL/min) to yield **5** (t_R_ = 13 min, 200 mg). Fraction E3 was subjected to Sephadex LH-20 (MeOH) and further purified by semi-preparative HPLC (90%, MeOH–H_2_O, 2 mL/min) to yield **11** (t_R_ = 9.0 min, 8 mg). Fraction E4 was subjected to Sephadex LH-20 (MeOH) and silica gel CC (CHCl_3_–MeOH, 20:1) to afford **12** (5.5 mg) and **10** (7.2 mg). Fraction D was further purified by silica gel CC eluting with a gradient of CHCl_3_–MeOH (50:1–10:1), to yield five fractions (D1–D5). Fraction D3 was subjected to Sephadex LH-20 (MeOH) and silica gel CC (CHCl_3_–MeOH, 20:1) and further purified by semi-preparative HPLC (39%, MeOH–H_2_O, 2 mL/min) to yield **14** (t_R_ = 30.0 min, 6.0 mg). Fraction D4 was subjected to Sephadex LH-20 (MeOH) and further purified by semi-preparative HPLC (60%, MeOH–H_2_O, 2 mL/min) to yield **2** (t_R_ = 12.5 min, 30.3 mg). Fraction D5 was subjected to Sephadex LH-20 (MeOH) and silica gel CC (CHCl_3_–MeOH, 30:1) and further purified by semi-preparative HPLC (40%, MeOH–H_2_O, 2 mL/min) to yield **4** (t_R_ = 11.0 min, 5.5 mg). Fraction C was further purified by silica gel CC eluting with a gradient of CHCl_3_–MeOH (50:1–10:1), to yield five fractions (C1–C5). Fraction C2 was subjected to Sephadex LH-20 (MeOH) to obtain two mixtures (C2.1 and C2.2). C2.1 was purified by semi-preparative HPLC (40%, MeOH–H_2_O, 2 mL/min) to yield **3** (t_R_ = 12.0 min, 50.0 mg) and **7** (t_R_ = 13.5 min, 4.2 mg). C2.2 was applied to silica gel CC (CHCl_3_–MeOH, 40:1) to afford **6** (7.3 mg) and **9** (6.0 mg). Fraction B was further purified by silica gel CC eluting with a gradient of CHCl_3_–MeOH (50:1–10:1), to yield five fractions (B1–B5). Fraction B3 was subjected to Sephadex LH-20 (MeOH) followed by purification with PTLC (petroleum–acetone 3:1, CHCl_3_–MeOH, 20:1) to yield compounds **1** (6.0 mg), **13** (8.3 mg). Fraction B4 was subjected to Sephadex LH-20 (MeOH) and silica gel CC (CHCl_3_–MeOH, 25:1) to afford **8** (5.4 mg).

Neocyathin A (**1**). White powder; [*α*]_D_^25^ = −49.3 (*c* = 0.24, MeOH); UV (MeOH) *λ*_max_ (log *ε*): 230 (3.01), 300 (2.23) nm; IR (KBr): *ν*_max_ = 3436, 2966, 2103, 1690, 1456, 1380 cm^−1^; for ^1^H- and ^13^C- NMR data see Tables 1 and 2, respectively; HR-ESI-MS: *m/z* 371.1824 [M + Na]^+^ (calcd. for C_20_H_28_O_5_Na, 371.1834).

Neocyathin B (**2**). White solid; [*α*]_D_^25^ = −34.3 (*c* = 0.08, MeOH); UV (MeOH) *λ*_max_ (log *ε*): 204 (2.75), 243 (2.92) nm; ECD: λ (*c* = 1.0 mg/mL, MeCN) (nm) (Δ*ε*) = 320 (−5.61), 242 (−17.7), 206 (+43.4); IR (KBr): *ν*_max_ = 3401, 2926, 2863, 1684, 1374, 1215, 1031 cm^−1^; for ^1^H- and ^13^C- NMR data see Tables 1 and 2, respectively; HR-ESI-MS: *m/z* 357.2034 [M + Na]^+^ (calcd. for C_20_H_30_O_4_Na, 357.2042).

Neocyathin C (**3**). Yellow oil; [*α*]_D_^25^ = −107.0 (*c* = 0.45, MeOH); UV (MeOH) *λ*_max_ (log *ε*): 230 (2.74), 314 (1.88) nm; ECD: λ (*c* = 1.0 mg/mL, MeCN) (nm) (*Δε*) = 391 (−0.39), 326 (+1.54), 206 (−22.7); IR (KBr): *ν*_max_ = 3371, 2942, 2876, 1644, 1455, 1034 cm^−1^; for ^1^H- and ^13^C- NMR data see Tables 1 and 2, respectively; HR-ESI-MS: *m/z* 373.1980 [M + Na]^+^ (calcd. for C_20_H_30_O_5_Na, 373.1991).

Neocyathin D (**4**). White powder; [*α*]_D_^25^ = −23.8 (*c* = 0.08, MeOH); UV (MeOH) *λ*_max_ (log *ε*): 230 (3.20) nm; IR (KBr): *ν*_max_ = 3416, 2938, 1641, 1446, 1381, 1028 cm^−1^; for ^1^H- and ^13^C- NMR data see Tables 1 and 2, respectively; HR-ESI-MS: *m/z* 373.1979 [M + Na]^+^ (calcd. for C_20_H_30_O_5_Na, 373.1991).

Neocyathin E (**5**). White powder; [*α*]_D_^25^ = −82.0 (*c* = 0.17, MeOH); UV (MeOH) *λ*_max_ (log *ε*): 207 (2.68) nm; ECD: λ (*c* = 0.3 mg/mL, MeCN) (nm) (*Δε*) = 393 (−0.15), 327 (+0.32), 213 (−5.22); IR (KBr): *ν*_max_ = 3451, 2958, 2866, 2082, 1640, 1050 cm^−1^; for ^1^H- and ^13^C- NMR data see Tables 1 and 2, respectively; HR-ESI-MS: *m/z* 357.2032 [M + Na]^+^ (calcd. for C_20_H_30_O_4_Na, 357.2042).

Neocyathin F (**6**). White powder; [*α*]_D_^25^ = −77.9 (*c* = 0.19, MeOH); UV (MeOH) *λ*_max_ (log *ε*): 206 (2.99), 243 (3.07) nm; IR (KBr): *ν*_max_ = 3374, 2939, 2872, 1696, 1384, 1031 cm^−1^; for ^1^H- and ^13^C- NMR data see Tables 1 and 2, respectively; HR-ESI-MS: *m/z* 371.1825 [M + Na]^+^ (calcd. for C_20_H_28_O_5_Na, 371.1834).

Neocyathin G (**7**). White powder; [*α*]_D_^25^ = −66.5 (*c* = 0.18, MeOH); UV (MeOH) *λ*_max_ (log *ε*): 207 (3.49) nm; IR (KBr): *ν*_max_ = 3369, 2933, 2870, 1648, 1046, 982 cm^−1^; for ^1^H- and ^13^C- NMR data see Tables 1 and 2, respectively; HR-ESI-MS: *m/z* 373.1982 [M + Na]^+^ (calcd. for C_20_H_30_O_5_Na, 373.1991).

Neocyathin H (**8**). Yellow oil; [*α*]_D_^25^ = −105.7 (*c* = 0.09, MeOH); UV (MeOH) *λ*_max_ (log *ε*): 237 (3.05) nm; ECD: λ (*c* = 0.7 mg/mL, MeCN) (nm) (*Δε*) = 329 (−2.10), 234 (+10.4), 199 (−21.6); IR (KBr): *ν*_max_ = 3417, 2962, 2874, 1689, 1593, 1383 cm^−1^; for ^1^H- and ^13^C- NMR data see Tables 1 and 2, respectively; HR-ESI-MS: *m/z* 371.1825 [M + Na]^+^, (calcd. for C_20_H_28_O_5_Na, 371.1834).

Neocyathin I (**9**). White solid; [*α*]_D_^25^ = −47.1 (*c* = 0.09, MeOH); UV (MeOH) *λ*_max_ (log *ε*): 231 (3.30) nm; IR (KBr): *ν*_max_ = 2961, 2384, 2310, 1738, 1366, 1218 cm^−1^; for ^1^H- and ^13^C- NMR data see Tables 1 and 2, respectively; HR-ESI-MS: *m/z* 373.1985 [M + Na]^+^ (calcd. for C_20_H_30_O_5_Na, 373.1991).

Neocyathin J (**10**). White powder; [*α*]_D_^25^ = −68.4 (*c* = 0.07, MeOH); UV (MeOH) *λ*_max_ (log *ε*): 230 (3.46) nm; IR (KBr): *ν*_max_ = 2935, 1735, 1687, 1606, 1369, 1217 cm^−1^; for ^1^H- and ^13^C- NMR data see Tables 1 and 2, respectively; HR-ESI-MS: *m/z* 373.1985 [M + Na]^+^ (calcd. for C_20_H_30_O_5_Na, 373.1991).

### Computational section

A preliminary conformational search was performed in Conflex6.7 using MMFF94s force field, according to previously reported methods^33, 34^. Conformers were saved and further optimized using the density functional theory (DFT) method and CPCM solvent model at B3LYP/6–31 + G(d,p) level in Gaussian 09 software package. Frequency was calculated at the same level of theory to check optimized results. The four low-energy conformers with populations greater than 1% and without imaginary frequencies were submitted to ECD calculation by the TDDFT (cam-B3LYP/TZVP) method associated with CPCM solvent model in MeCN. The excitation energies (E), oscillator strength (f), rotatory strength in velocity form (R_vel_), and rotatory strength in length form (R_len_) of the lowest 32 excited states were calculated. ECD spectra of different conformers were summated in SpecDis 1.62 according to their Boltzmann-calculated distributions. The summated curve was adjusted +5 nm UV-shift and 0.3 ev.

### Cell culture and treatment

Murine microglia cell line BV2 cells was collected from ATCC and cultured in DMEM including 10% fetal bovine serum (FBS). Lipopolysaccharide (LPS) (Sigma, USA) were dissolved in PBS at a concentration of 1 mg/mL, while Aβ_1–42_ (GL Biochem (Shanghai) Ltd, China) was dissolved in 1% NH_3_·H_2_O at the concentration of 2 mM and incubated at 37 °C for 7 days to allow fibril formation. For biological assays, BV2 cells were pretreated with all compounds at 50 μM for 1 h, and then stimulated with 0.5 μg/mL LPS and 20 μM Aβ_1–42_ for 24 h in DMEM with 3% FBS.

### Cell viability assay

Cell viability was measured by MTT assay. Briefly, BV2 microglia cells were seeded in 96-well plates overnight and treated with different concentrations of isolated compounds for 24 h or 72 h. After incubation and removal of culture medium, the BV2 cells were incubated with 0.5 mg/ml MTT in phosphate-buffered saline (PBS) for 4 h. Then the MTT was removed and resolved with 150 μL of DMSO. The production of purple formazan was detected for the absorbance at 570 nm on a Bio-Rad microplate reader (Hercules, CA, USA). The cell viability was presented as a percentage to that of controls.

### Western blot analysis

After drug treatment, the cellular proteins were extracted and analyzed by Western blotting analysis for protein expression as previously described^35^. Briefly, the cellular proteins were isolated and subjected to electrophoresis on 10% SDS-polyacrylamide gels and transferred onto a polyvinylidenedifluoride (PVDF) membrane. After overnight incubation in 5% BSA in the mixture of Tris-buffered saline and Tween-20 (TBS-T), the membranes were probed with primary antibodies: iNOS (Mouse monoclonal antibody, 1:1000, Abcam), COX-2 (Rabbit monoclonal antibody, 1:1000) and GAPDH (Rabbit monoclonal antibody, 1:1000) antibodies (Cell Signaling Technology, Boston, MA, USA) for 4 h and subsequently detected by goat anti-rabbit IgG-HRP conjugate secondary antibodies. The activity of peroxidase retained on the blots was measured by enhanced chemiluminescence (ECL) detection reagents from GE Healthcare (Uppsala, Sweden) according to the manufacturer’s instruction.

### Assay for iNOS enzyme activity

The activity of iNOS enzyme was measured by using nitric oxide synthase activity assay kit (Abcam, Cambridge, UK) following the manufacturer’s instruction. In brief, after drug treatment, the cells were harvested and washed by cold PBS. The cells were resuspended in cold NOS assay buffer containing protease inhibitor on ice. After pipetting up and down, the samples were centrifuged at 4 °C at 10000 × g for 10 min. The supernatants were collected and subjected to the measurement of the protein concentration using a BCA protein assay kit. Sixty microliters of 100 μg protein sample and 45 μL of reaction mix including diluted NOS cofactor 1, NOS cofactor 2 (1X), NOS substrate and nitrate reductase were added into each well, mixed well and incubated for 1 h at 37 °C. Then, 90 μL of NOS assay buffer and 5 μL of enhancer were added into each well, mixed and incubated at RT for 10 min. After that, 100 μL of Griess reagent mixture were added and incubated at RT for 10 min. The absorbance was immediately measured on a microplate reader at OD 540 nm.

### Measurement of NO production in culture medium

After drug treatment, nitrite in culture medium was determined to reflect NO production by using Griess reagent kit for nitrite determination (ThermoFisher Scientific, Waltham, MA, USA). Briefly, 150 μL culture medium, 130 μL milli-Q water and 20 μL Griess reagent (mixture of equal volume of N-(1-naphthyl)ethylenediamine dihydrochloride and sulfanilic acid) were added into 96 well plate, and incubated at room temperature for 30 min. The absorbance of samples was measured on a spectrophotometric microplate reader at 540 nm.

### Molecular docking studies

Molecular docking simulations were performed using the software Autodock 4.2 Vina along with AutoDock Tools (ADT 1.5.6) using the hybrid Lamarckian Genetic Algorithm (LGA) as our previous studies^30^. The three dimensional (3D) crystal structure of iNOS (PDB code: 3E7G) and COX-2 (PDB code: 1CX2) were obtained from the RCSB Protein Data Bank. The standard 3D structure (PDB format) of compounds **11** and **14** were constructed by using the “SKETCH” option function in SYBYL-X, whose configurations were determined by their NOESY spectra and TDDFT ECD calculations. The cubic grid box of 44 Å size (x, y, z) with a spacing of 0.375 Å and grid maps were built. The docking parameters consisted of setting the population size to 150, the number of evaluations to 2,500,000, the number of generations to 270,000, and the number of top individuals that automatically survive to 20, while the number of docking run was set to 40 with other default values during each docking run. The results of the most favorable free energy of binding were chosen as the resultant complex structures.

### Statistical analysis

The results were expressed as means ± SD. The significance was analyzed by one-way ANOVA with Dunnett’s test using Graphpad Prism 5 software and LSD test with IBM SPSS Statistics 19.0 software. A p-value of < 0.05 was considered as statistically significant.

## Electronic supplementary material

Supplementary information
