# Supplementary material for: Molecular Diversity and Potential Anti-neuroinflammatory Activities of Cyathane Diterpenoids from the Basidiomycete Cyathus africanus
Source: Sci Rep. 2017 Aug 21;7:8883. doi: 10.1038/s41598-017-09118-z (PMC5567052; doi:10.1038/s41598-017-09118-z)
Supplement: Supplementary file 1 — Supplementary information [file 41598_2017_9118_MOESM1_ESM.pdf]

Supplementary information

Molecular Diversity and Potential Anti-neuroinflammatory Activities of Cyathane Diterpenoids from the Basidiomycete *Cyathus africanus*

Jing Wei[1,§], Yuanyuan Cheng[2,§], Wan-Hui Guo[1], Da-Cheng Wang[1], Qiang Zhang[1], Ding Li[1], Jianhui Rong[2,*] & Jin-Ming Gao[2,*]

[1]Shaanxi Key Laboratory of Natural Products & Chemical Biology, Shaanxi Engineering Center of Bioresource Chemistry & Sustainable Utilization, College of Chemistry & Pharmacy, Northwest A&F University, Yangling 712100, Shaanxi, People's Republic of China.

[2]School of Chinese Medicine, Li Ka Shing Faculty of Medicine, University of Hong Kong, 10 Sassoon Road, Pokfulam, Hong Kong, People's Republic of China.

§ both authors contributed equally to this work

* Corresponding author:

Fax/Tel: +86-29-87092335.

E-mail: jinminggao@nwsuaf.edu.cn (J.M.G.); jrong@hku.hk (J. R.)

Content

Table S1. ^{1}H NMR Data for Compounds **11-14**........ 3
Table S2. ^{13}C NMR Data for Compounds **11-14**........ 4
Figure S1. ^{1}H NMR and ^{13}C spectra of compound **1** in CD_3OD. 5
Figure S2. HSQC and ^{1}H-^{1}H COSY spectra of compound **1** in CD_3OD........ 5
Figure S3. HMBC and NOESY spectra of compound **1** in CD_3OD and DMSO-d_6. 6
Figure S4. HRESIMS spectrum of compound **1**........ 7
Figure S5. IR and UV spectra of compound**1**........ 8
Figure S6. ^{1}H NMR and ^{13}C spectra of compound **2** in CD_3OD. 9
Figure S7. HSQC and ^{1}H-^{1}H COSY spectra of compound **2** in CD_3OD........ 10
Figure S8. HMBC and NOESY spectra of compound **2** in CD_3OD. 11
Figure S9. HRESIMS spectrum of compound **2**........ 12
Figure S10. IR and UV spectra of compound **2**........ 13

Figure S11. ^{1}H NMR and ^{13}C spectra of compound **3** in CD_3OD. 14
Figure S12. HSQC and ^{1}H-^{1}H COSY spectra of compound **3** in CD_3OD. 15
Figure S13. HMBC and NOESY spectra of compound **3** in CD_3OD. 16
Figure S14. HRESIMS spectrum of compound **3**. 17
Figure S15. IR and UV spectra of compound **3**. 18
Figure S16. ^{1}H NMR and ^{13}C spectra of compound **4** in CD_3OD. 19
Figure S17. HSQC and ^{1}H-^{1}H COSY spectra of compound **4** in CD_3OD. 20
Figure S18. HMBC and NOESY spectra of compound **4** in CD_3OD. 21
Figure S19. HRESIMS spectrum of compound **4**. 22
Figure S20. IR and UV spectra of compound **4**. 23
Figure S21. ^{1}H NMR and ^{13}C spectra of compound **5** in CD_3OD. 24
Figure S22. HSQC and ^{1}H-^{1}H COSY spectra of compound **5** in CD_3OD. 25
Figure S23. HMBC and NOESY spectra of compound **5** in CD_3OD. 26
Figure S24. HRESIMS spectrum of compound **5**. 27
Figure S25. IR and UV spectra of compound **5**. 28
Figure S26. ^{1}H NMR and ^{13}C spectra of compound **6** in CD_3OD. 28
Figure S27. HSQC and ^{1}H-^{1}H COSY spectra of compound **6** in CD_3OD. 29
Figure S28. HMBC and NOESY spectra of compound **6** in CD_3OD. 30
Figure S29. HRESIMS spectrum of compound **6**. 31
Figure S30. IR and UV spectra of compound **6**. 32
Figure S31. ^{1}H NMR and ^{13}C spectra of compound **7** in CD_3OD. 33
Figure S32. HSQC and ^{1}H-^{1}H COSY spectra of compound **7** in CD_3OD. 34
Figure S33. HMBC and NOESY spectra of compound **7** in CD_3OD. 35
Figure S34. HRESIMS spectrum of compound **7**. 36
Figure S35. IR and UV spectra of compound **7**. 37
Figure S36. ^{1}H NMR and ^{13}C spectra of compound **8** in CD_3OD. 38
Figure S37. HSQC and ^{1}H-^{1}H COSY spectra of compound **8** in CD_3OD. 39
Figure S38. HMBC and NOESY spectra of compound **8** in CD_3OD. 40
Figure S39. HRESIMS spectrum of compound **8**. 41
Figure S40. IR and UV spectra of compound **8**. 42
Figure S41. ^{1}H NMR and ^{13}C spectra of compound **9** in CD_3OD. 43
Figure S42. HSQC and ^{1}H-^{1}H COSY spectra of compound **9** in CD_3OD. 44
Figure S43. HMBC and NOESY spectra of compound **9** in CD_3OD. 45
Figure S44. HRESIMS spectrum of compound **9**. 46
Figure S45. IR and UV spectra of compound **9**. 47
Figure S46. ^{1}H NMR and ^{13}C spectra of compound **10** in CD_3OD. 48
Figure S47. HSQC and ^{1}H-^{1}H COSY spectra of compound **10** in CD_3OD. 49
Figure S48. HMBC and NOESY spectra of compound **10** in CD_3OD. 50
Figure S49. HRESIMS spectrum of compound **10**. 51
Figure S50. IR and UV spectra of compound **10**. 52
Figure S51. ^{1}H NMR and ^{13}C spectra of compound **11** in CD_3OD. 52
Figure S52. ESIMS spectrum of compound **11**. 53
Figure S53. UV spectra of compound **11**. 54
Figure S54. ^{1}H NMR and ^{13}C spectra of compound **12** in C_5D_5N. 54

Figure S55. ESIMS spectrum of compound **12**. 55
Figure S56. UV spectra of compound **12**. 56
Figure S57. ^{1}H NMR and ^{13}C spectra of compound **13** in CD_3OD. 56
Figure S58. ESIMS spectrum of compound **13**. 57
Figure S59. UV spectra of compound **13**. 58
Figure S60. ^{1}H NMR and ^{13}C spectra of compound **14** in CD_3OD. 58
Figure S61. ESIMS spectrum of compound **14**. 59
Figure S62. UV spectra of compound **14**. 60
Figure S63. Cytotoxicity of compounds in BV2 microglia cells. 61

No.	**11** (CD_3OD)	**12** (C_5D_5N)	**13** (CD_3OD)	**14** (CD_3OD)
1	6.24 d (5.4)	1.52 m 2H	2.17 m 2H	
2	6.32 d (5.4)	2.23 m 2H		6.02 m
4				2.95 m
5	2.42dd (13.0, 3.7)	2.60 m	2.83dd (13.0, 4.0)	2.39 m
7	1.61td (13.5, 4.0)<br>1.44ddd (13.5, 4.3, 2.4)	1.79 m<br>1.64 m	1.70 m<br>1.55 m	2.01 m<br>1.05 m
8	1.71ddd (13.5, 4.0, 2.4)<br>1.26 td (13.5, 4.3)	1.71 td (13.2, 4.4)<br>1.59 m	1.82 m<br>1.61 m	1.70 m<br>1.33 m
10	2.31 m<br>1.52ddd (12.7, 3.5, 2.0)	2.33 td (13.1, 3.8)<br>1.98dd (13.1, 3.6)	2.36 td (13.3, 4.0)<br>1.75 m	2.41 m<br>2.33 m
11	4.22 m	4.72 m	4.33 m	4.39 m
12	2.35 m	3.16 m	2.47 m	
13	4.52 d (7.7)	4.57 d (4.7)	4.13 d (5.0)	6.06 m
15	3.85dd (10.9, 5.9)<br>3.62dd (10.9, 8.7)	4.34dd (10.8, 6.8)<br>4.07 m	3.89dd (11.2, 6.1)<br>3.69dd (11.2, 9.2)	4.35 m<br>4.26 m
16	0.87 s	1.42 s	1.07 s	1.09 s
17	1.02 s	0.97 s	1.25 s	1.11 s
18	2.99 m	2.99 m	3.09 m	2.89 m
19	1.05 d (6.9)	0.85 d (6.8)	1.18 d (7.0)	1.29 d (6.6)
20	1.11 d (6.9)	1.01 d (6.8)	1.20 d (7.0)	1.27 d (6.6)

Table S1. ^{1}H NMR Data for Compounds **11-14.**

No.	**11**(CD_3OD)	**12**(C_5D_5N)	**13**(CD_3OD)	**14**(CD_3OD)
1	145.8	40.1	53.2	216.1
2	128.9	29.2	210.8	127.8
3	143.0	140.1	144.8	193.5
4	143.1	136.6	176.9	56.2
5	38.8	38.6	40.8	37.8
6	46.5	43.9	46.3	51.0
7	29.8	29.9	29.3	32.6
8	32.4	37.7	37.1	31.3
9	55.1	48.3	42.8	50.4
10	31.6	27.8	27.4	38.7
11	76.9	73.7	74.4	72.6
12	49.5	56.2	55.8	156.2
13	70.2	71.7	71.6	123.1
14	107.8	106.1	106.2	209.3
15	62.2	60.4	60.4	64.2
16	12.9	13.6	13.4	21.0
17	20.6	24.1	26.1	21.4
18	26.9	26.5	26.2	32.4
19	23.0	21.4	20.5	21.1
20	23.2	22.7	20.7	24.1

Table S2. ^{13}C NMR Data for Compounds **11-14.**

Figure S1. ^{1}H NMR and ^{13}C spectra of compound **1** in CD_3OD.

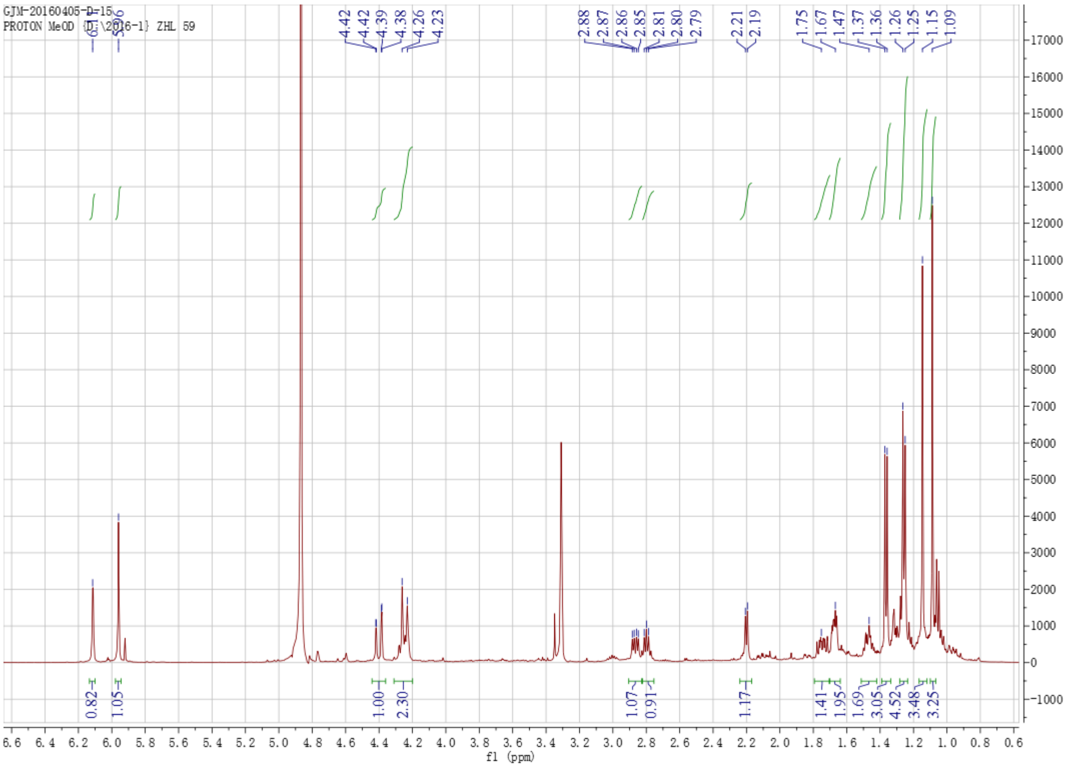

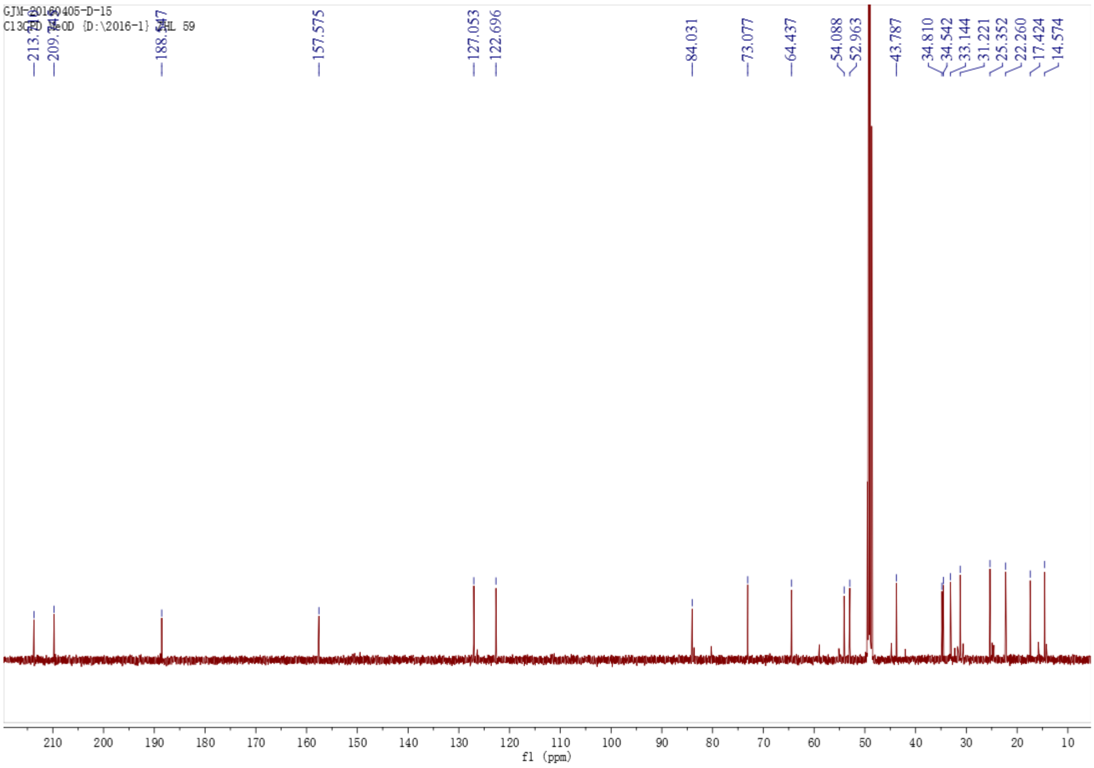

Figure S2. HSQC and ^{1}H-^{1}H COSY spectra of compound **1** in CD_3OD

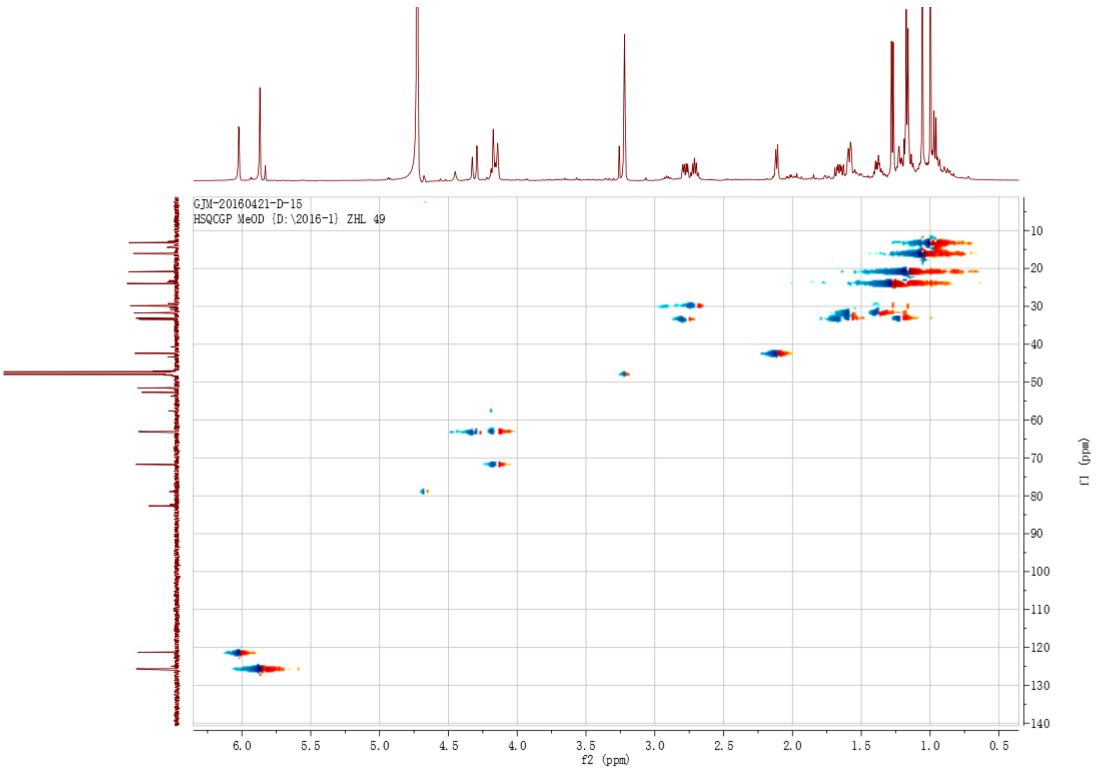

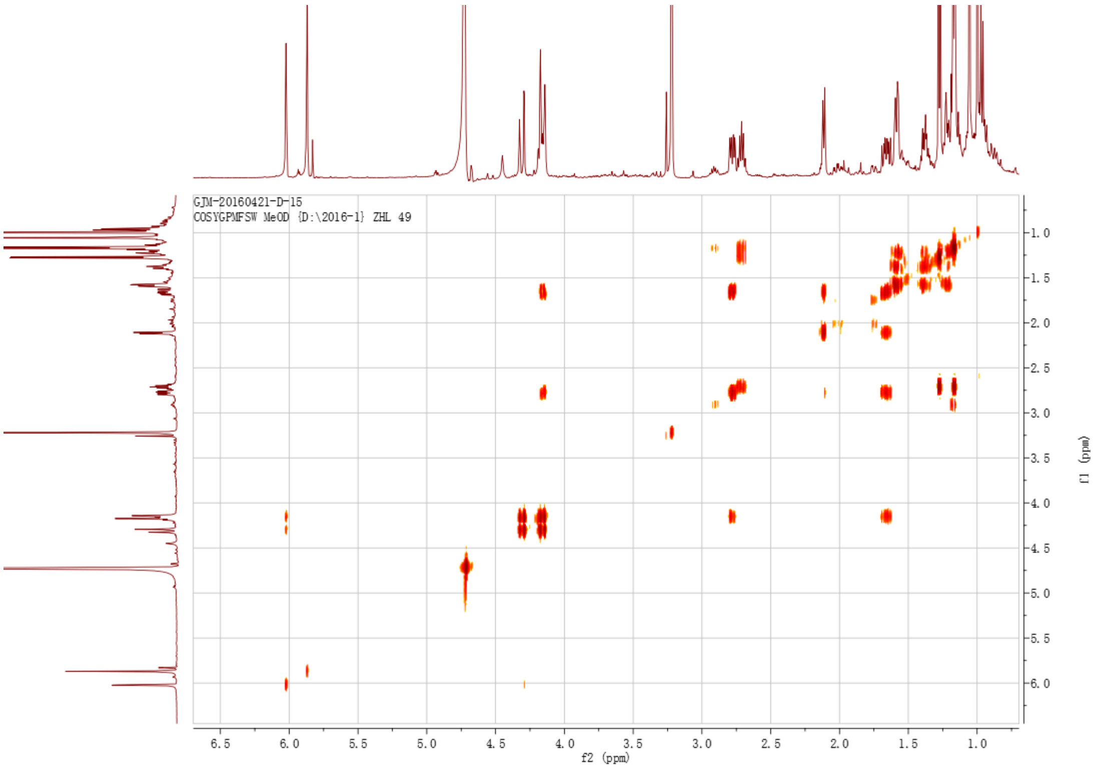

Figure S3. HMBC and NOESY spectra of compound **1** in CD_3OD and DMSO-d_6.

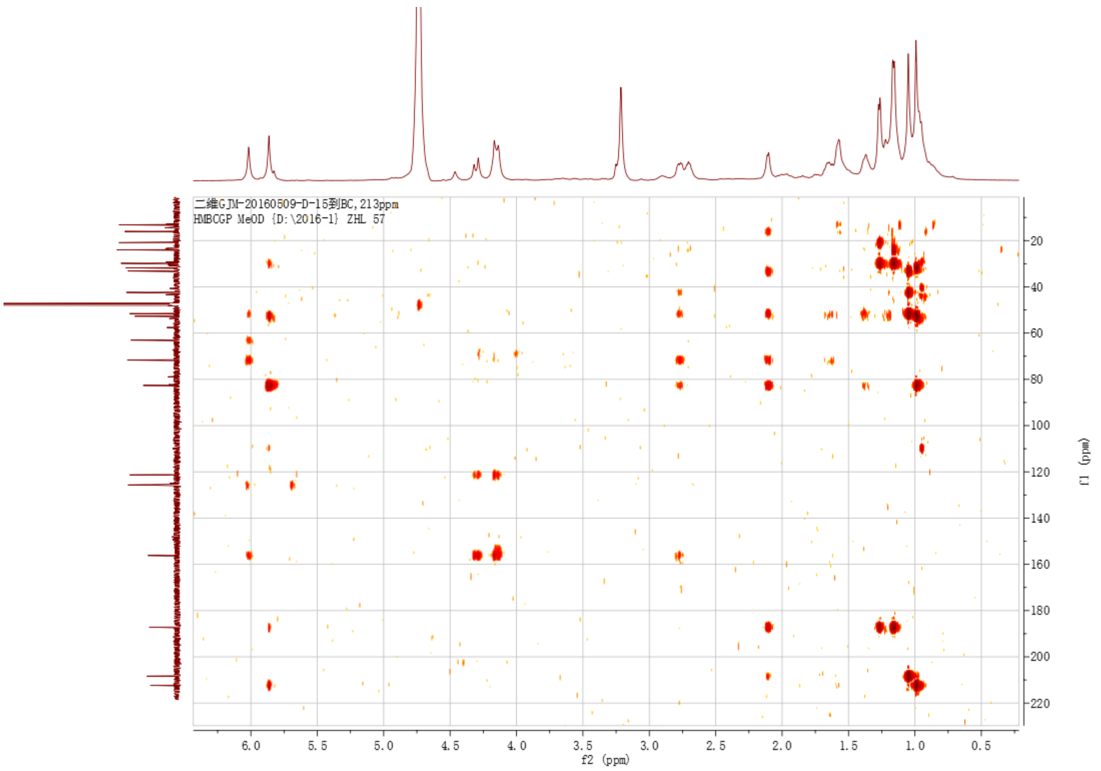

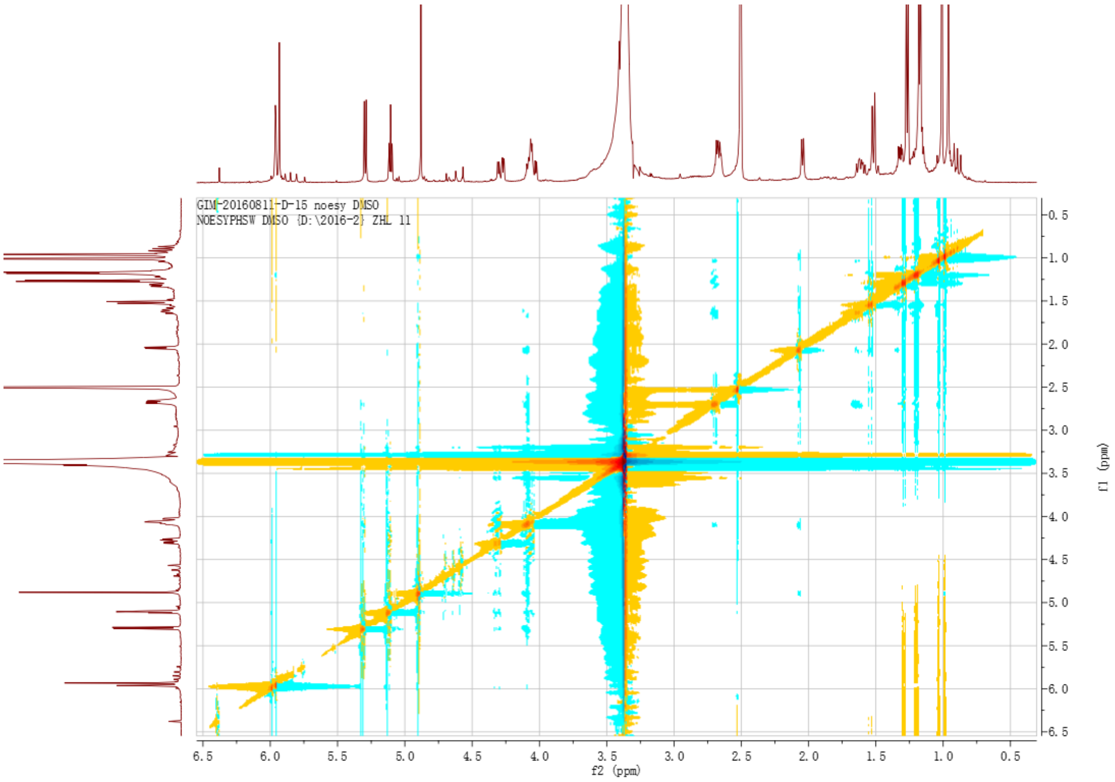

Figure S4. HRESIMS spectrum of compound **1**.

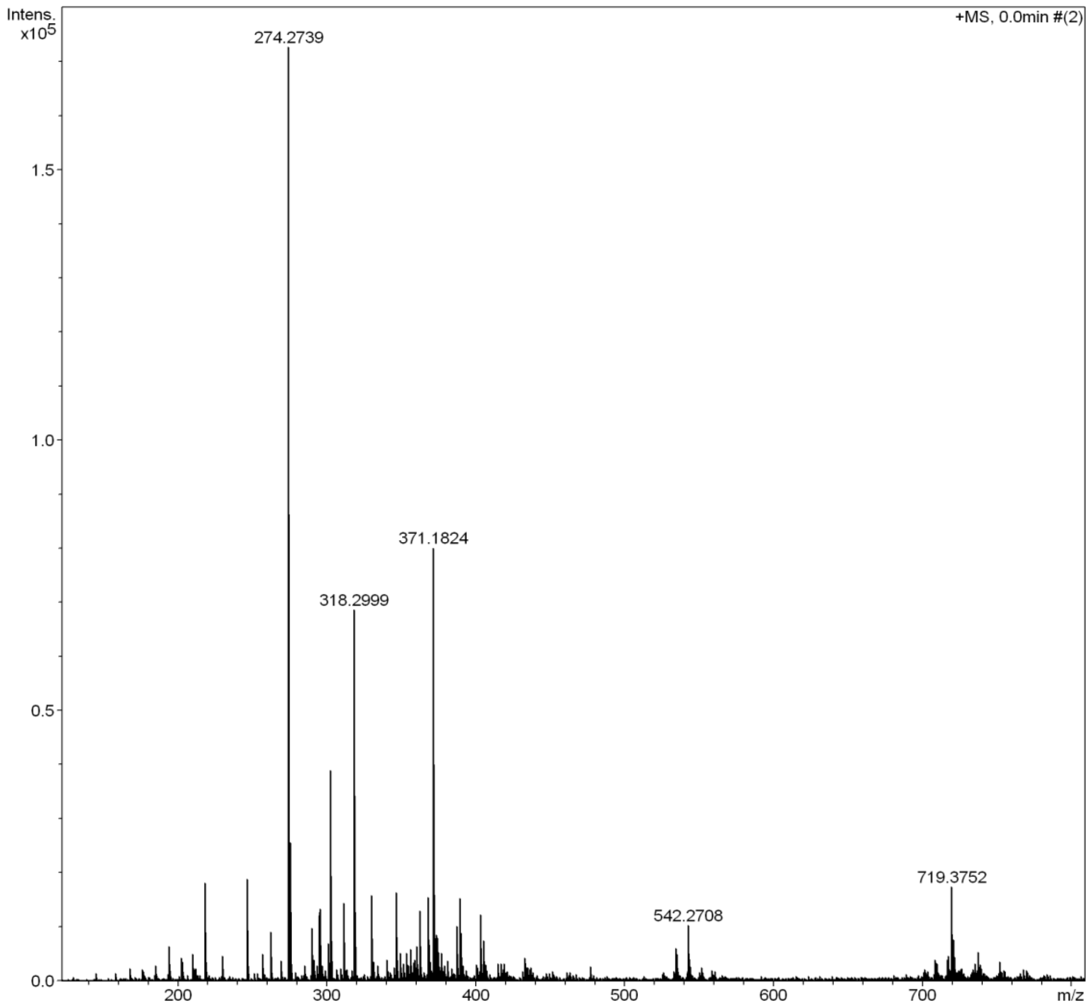

Figure S5. IR and UV spectra of compound **1**. This work is supported by Bruker (Beijing) Scientific Technology Co. Ltd.

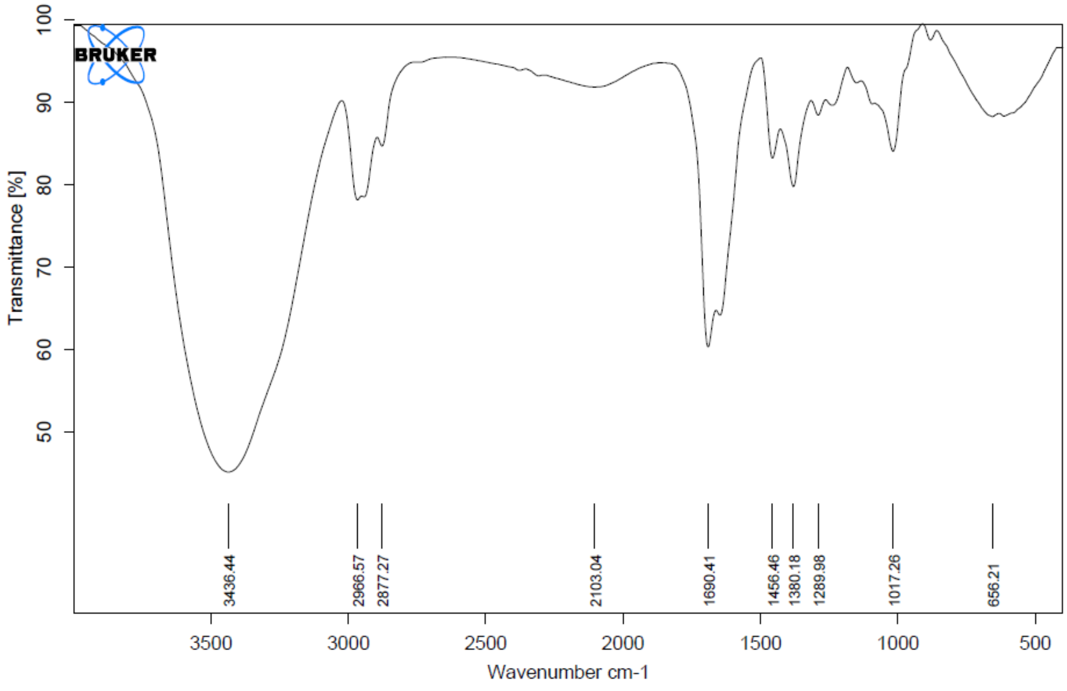

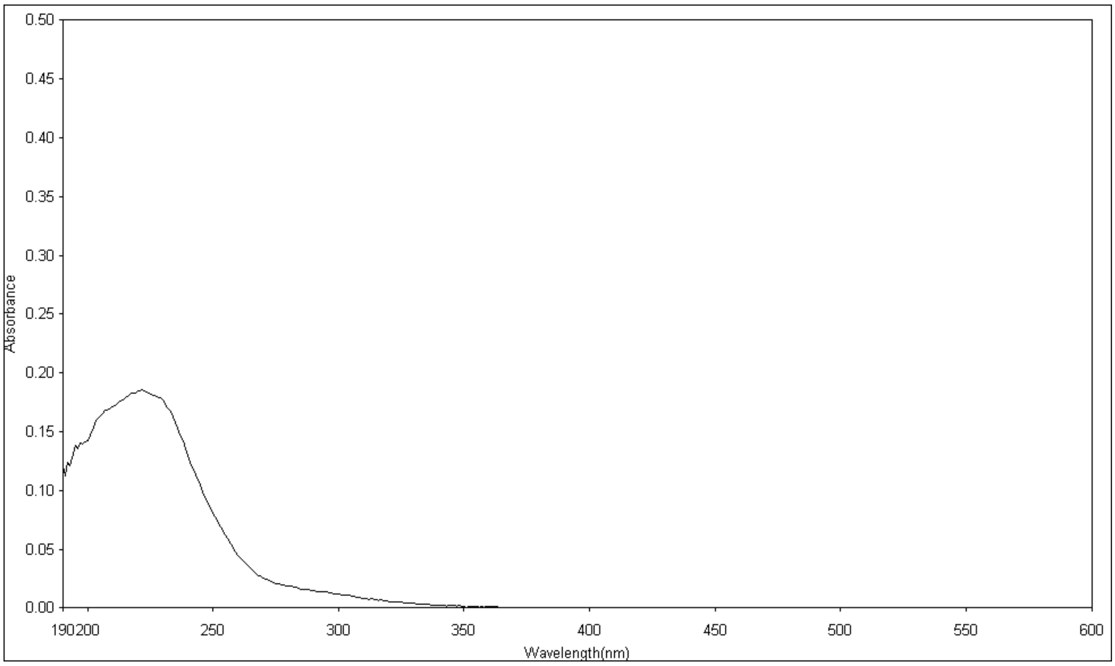

Figure S6. ^{1}H NMR and ^{13}C spectra of compound **2** in CD_3OD.

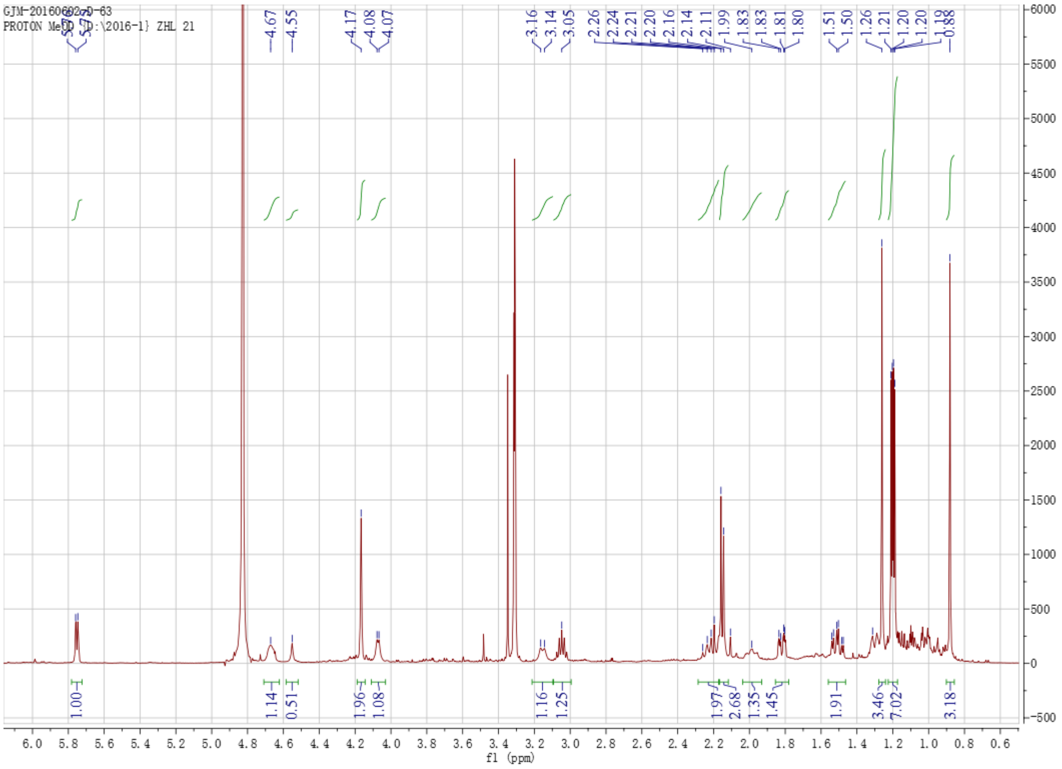

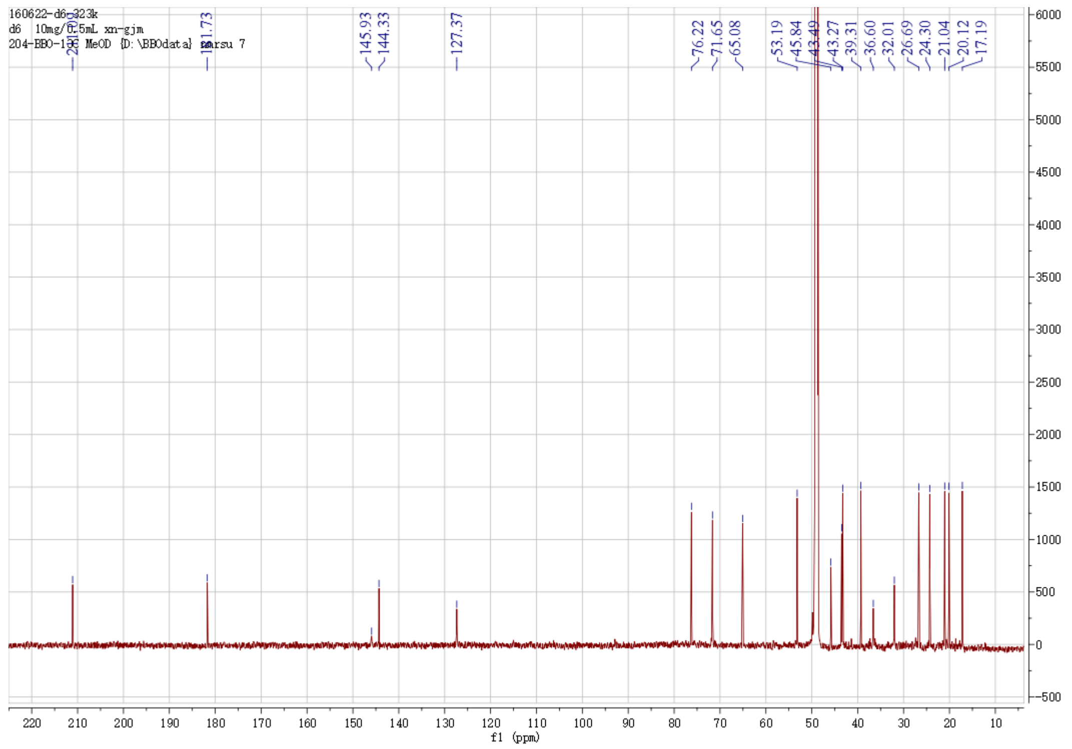

Figure S7. HSQC and ^1H-^1H COSY spectra of compound **2** in CD_3OD

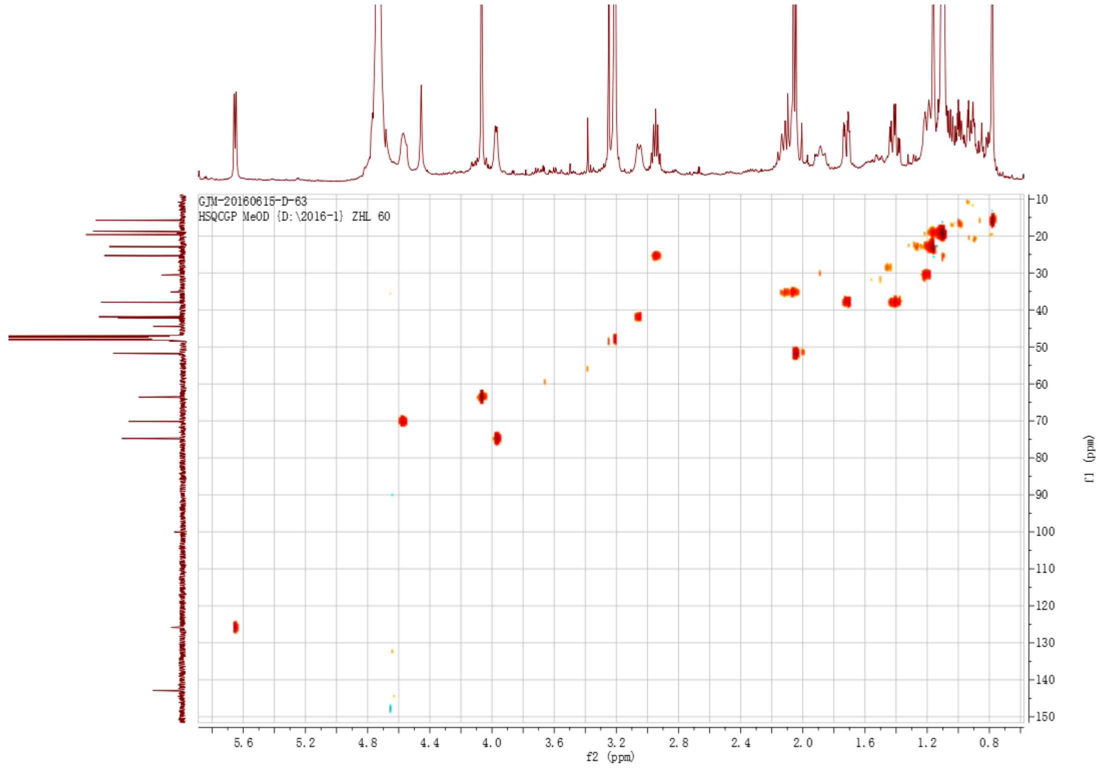

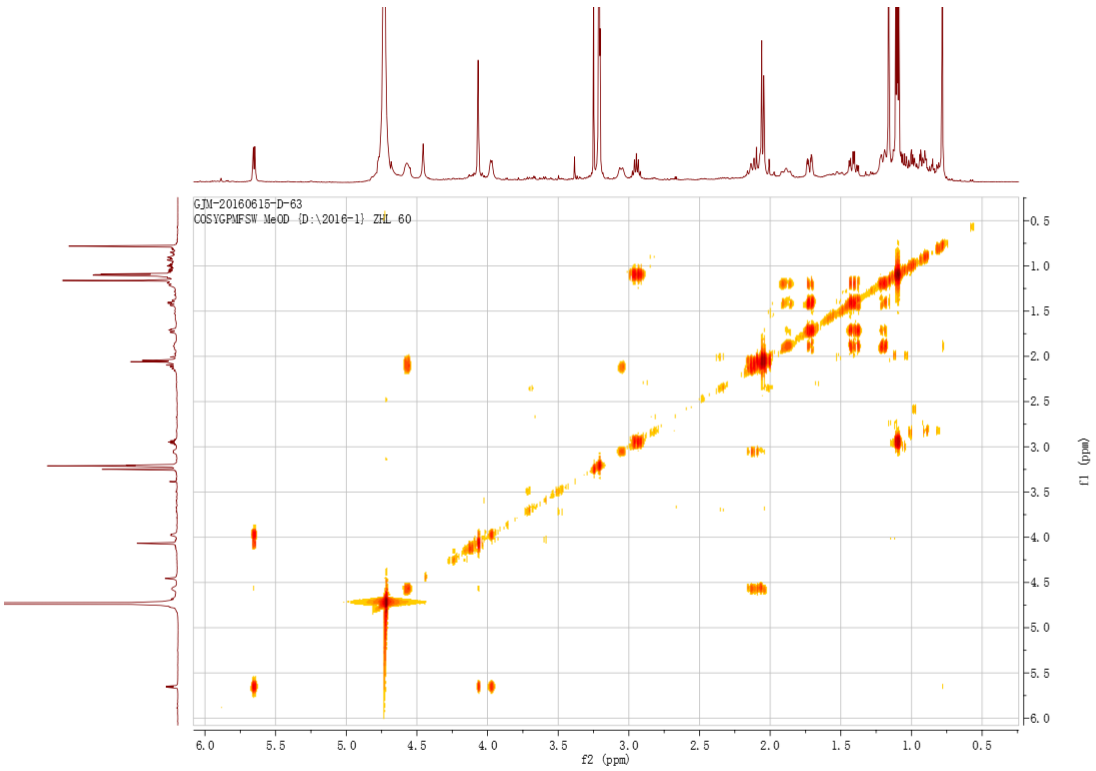

Figure S8. HMBC and NOESY spectra of compound **2** in CD_3OD.

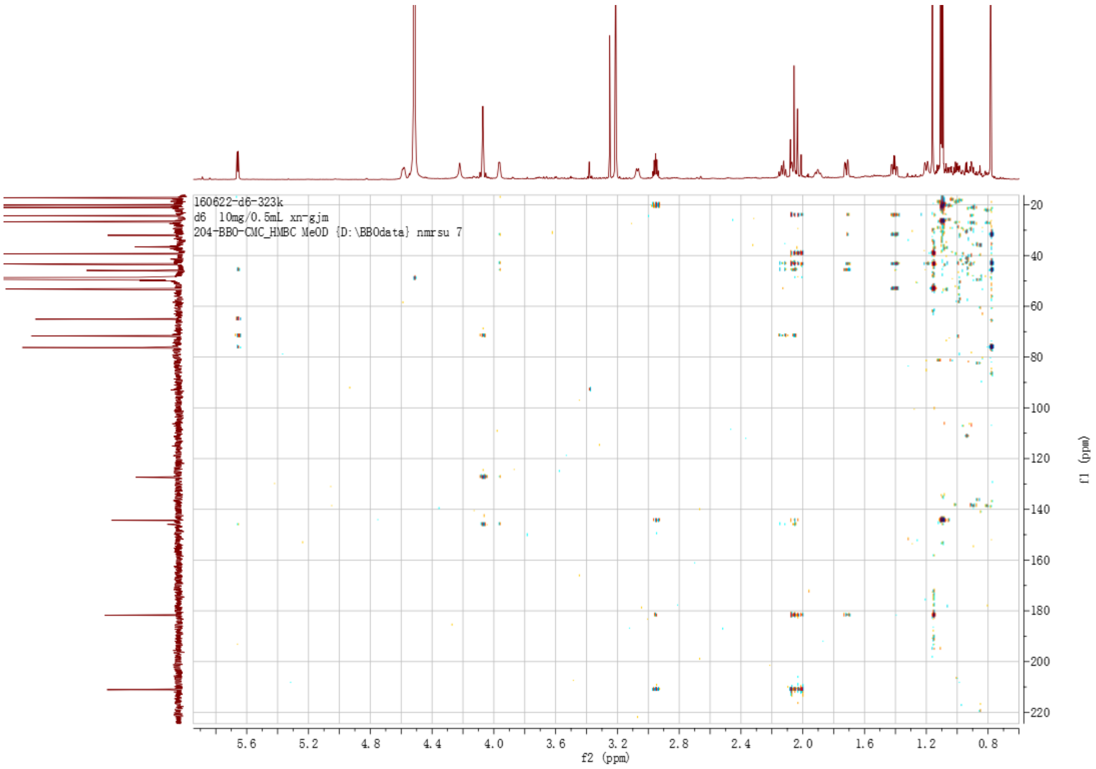

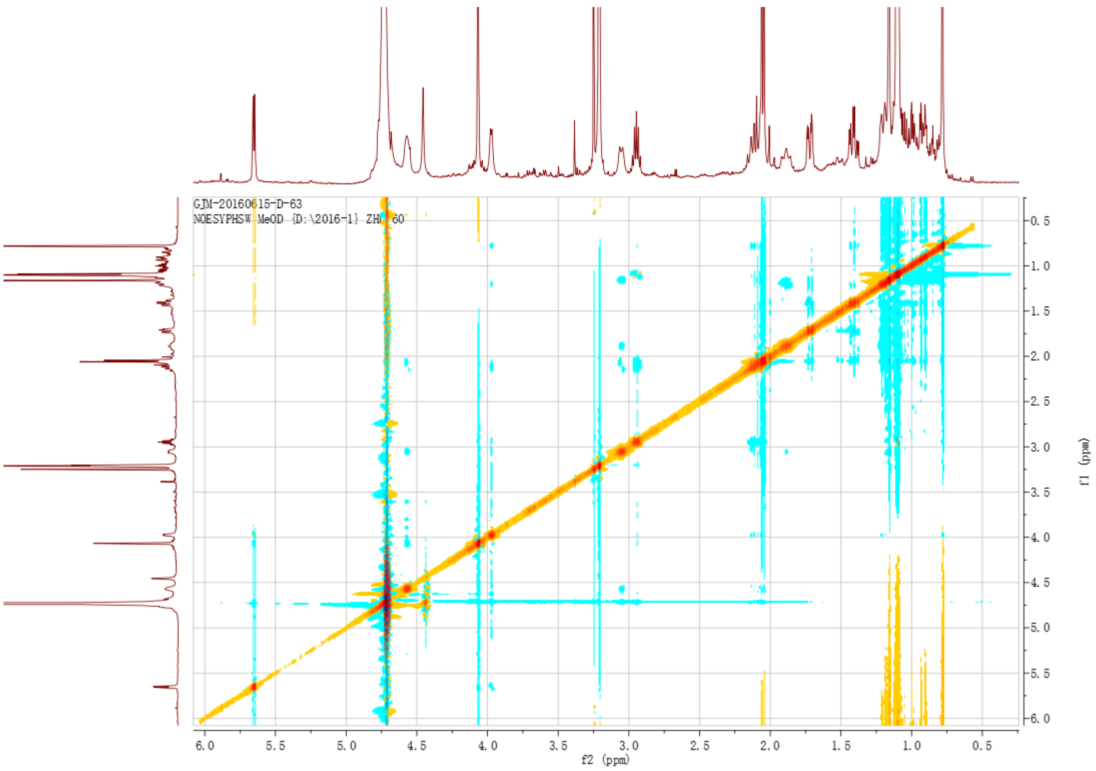

Figure S9. HRESIMS spectrum of compound **2**.

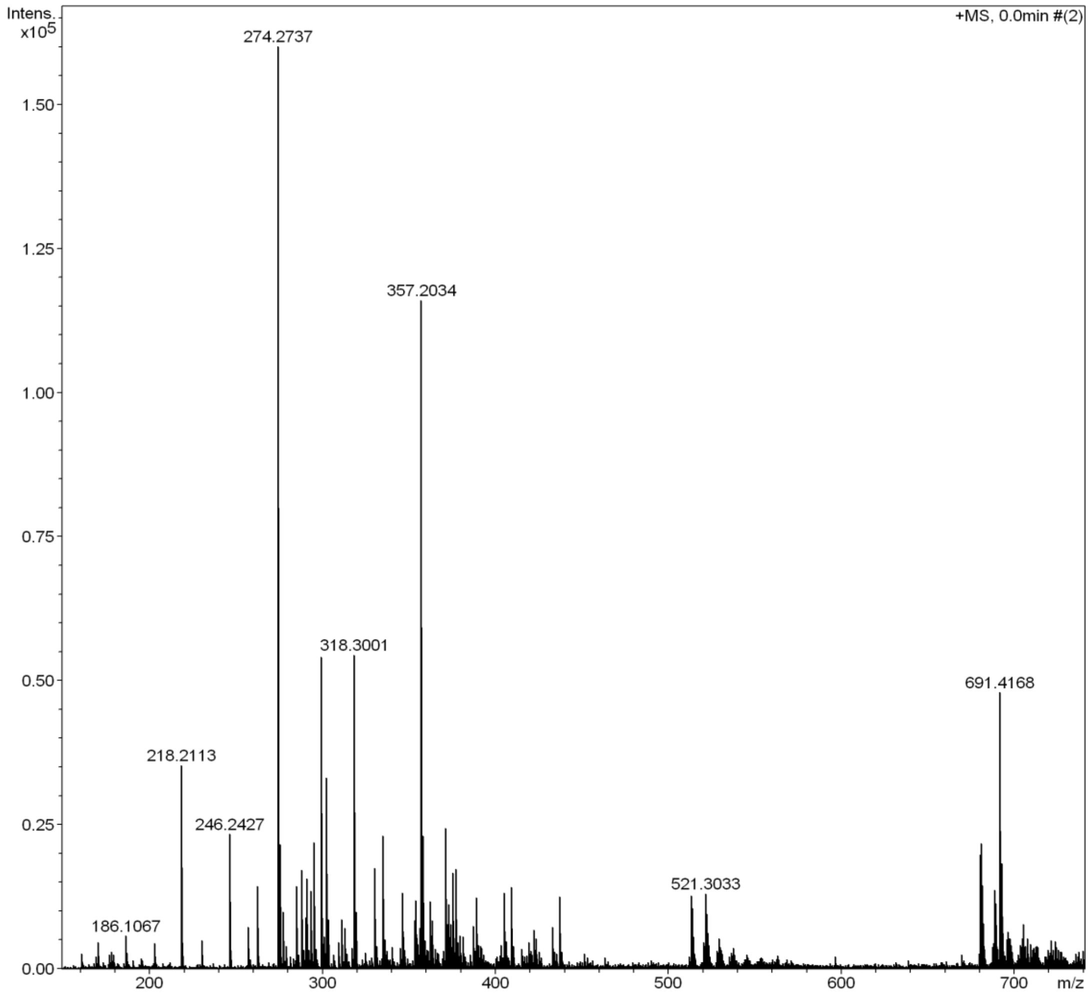

Figure S10. IR and UV spectra of compound **2**. This work is supported by Bruker (Beijing) Scientific Technology Co. Ltd.

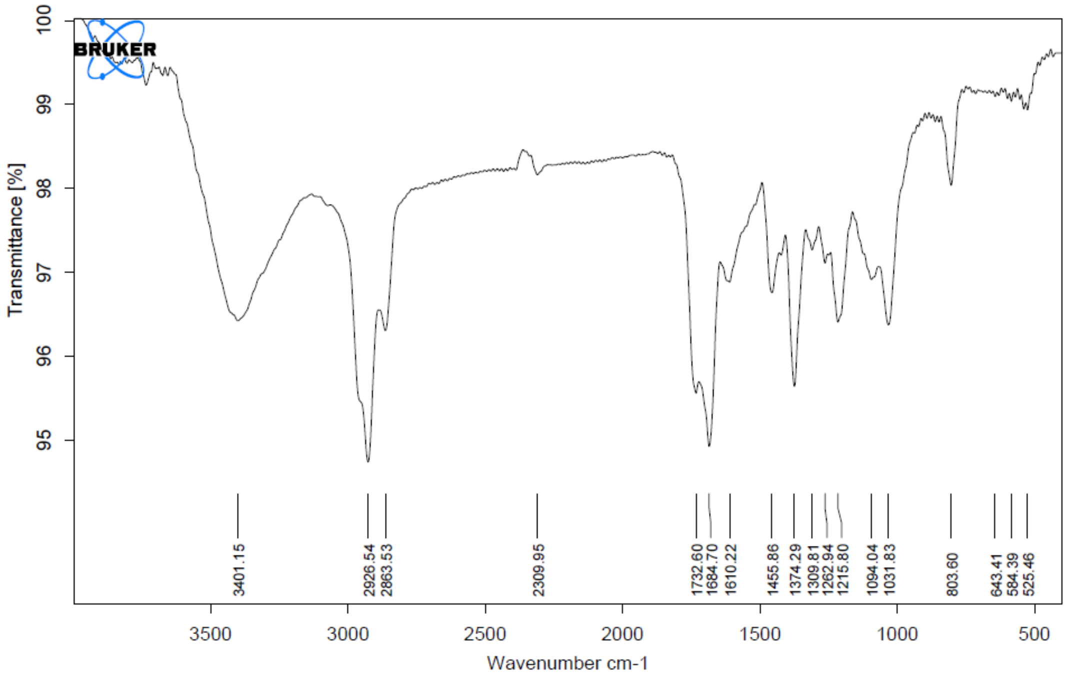

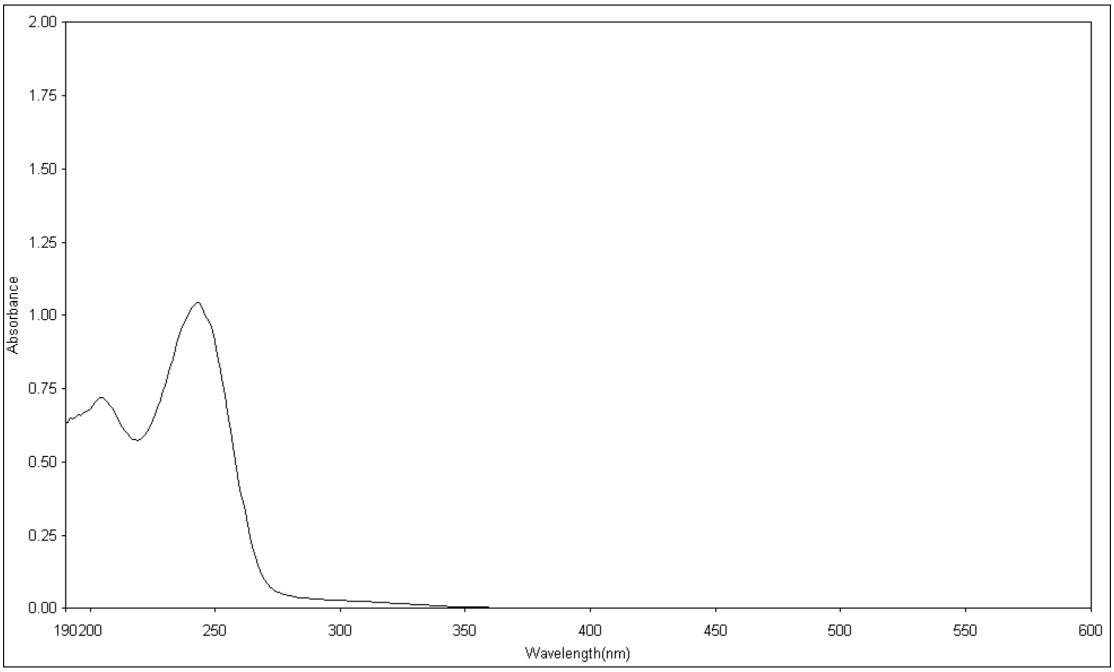

Figure S11. ^{1}H NMR and ^{13}C spectra of compound **3** in CD_3OD.

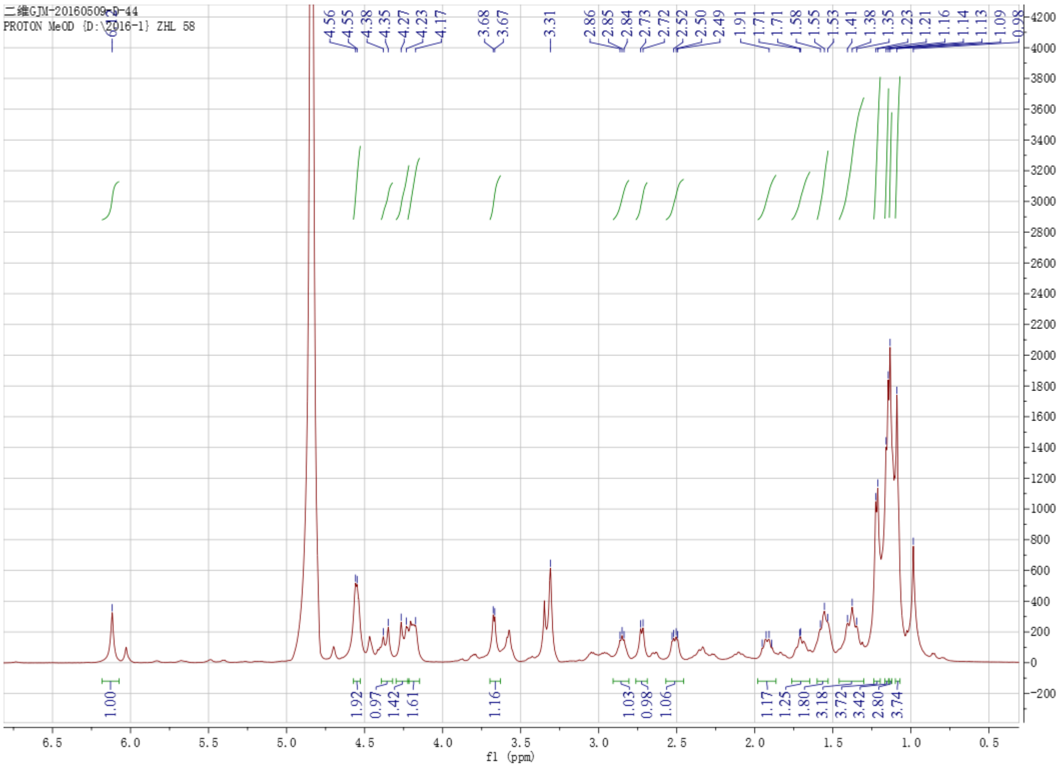

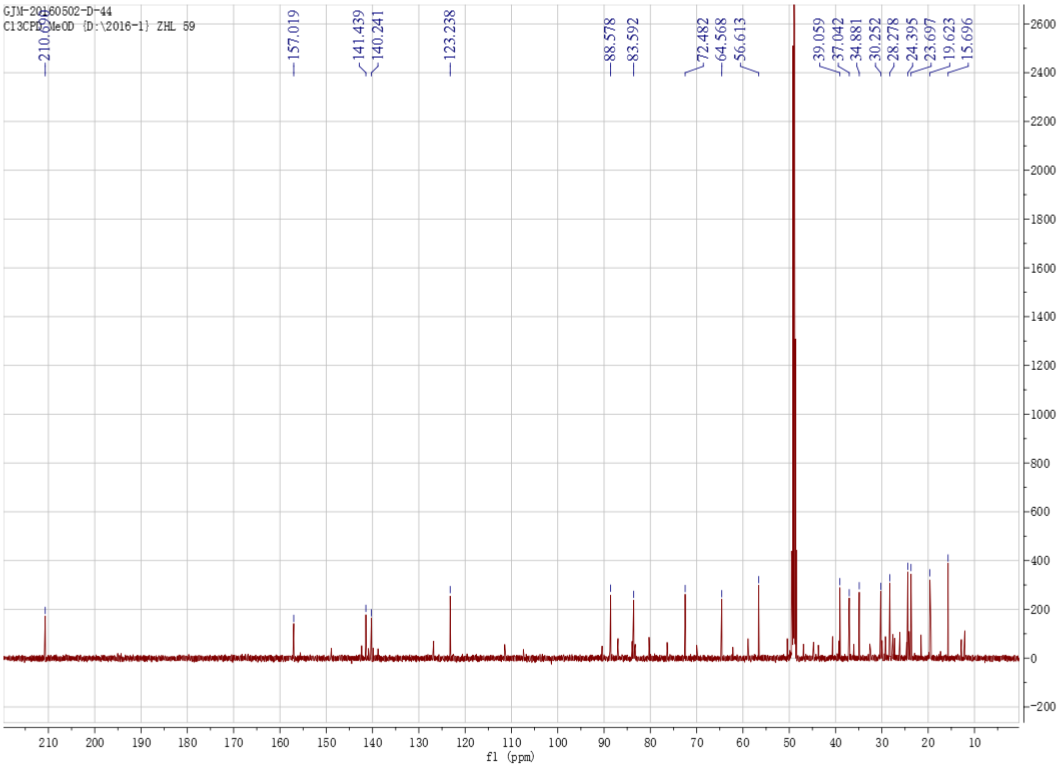

Figure S12. HSQC and ^{1}H-^{1}H COSY spectra of compound **3** in CD_3OD

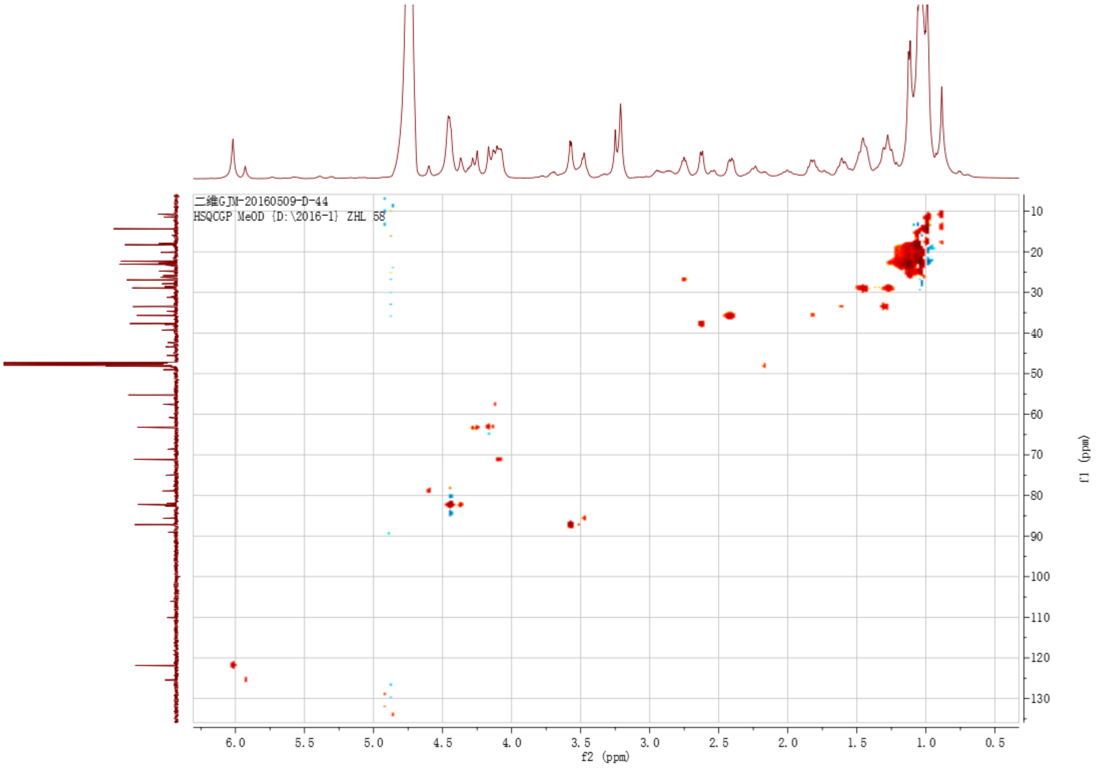

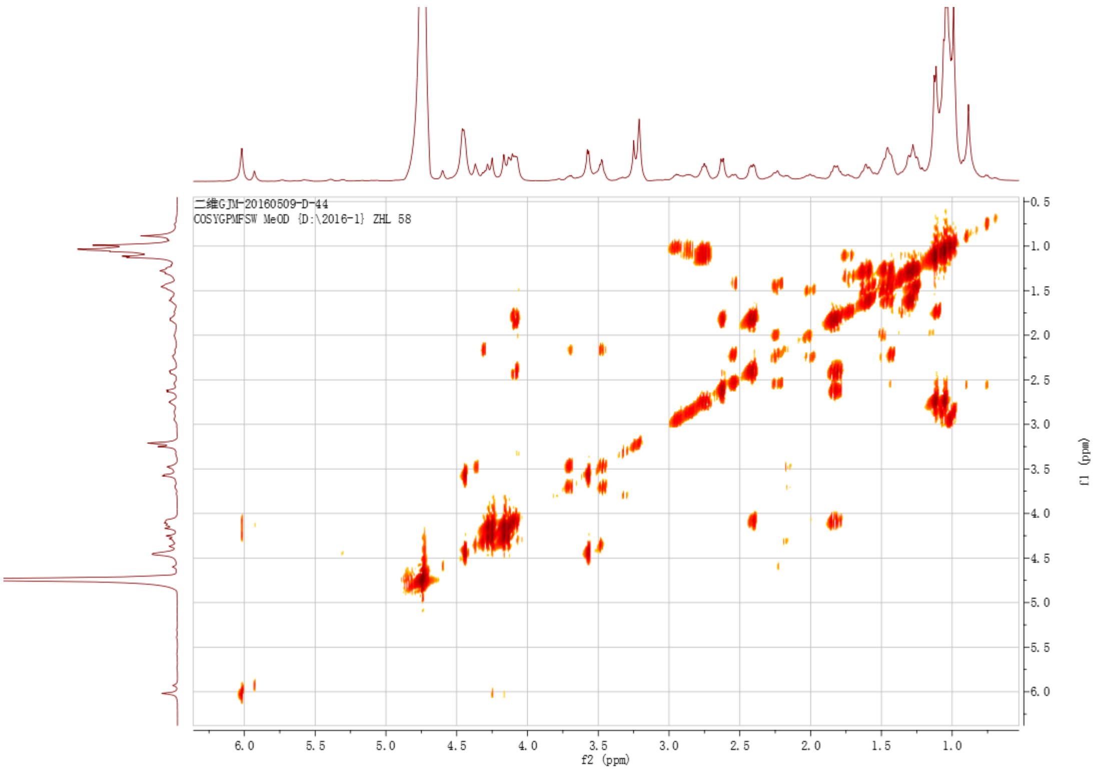

Figure S13. HMBC and NOESY spectra of compound **3** in CD_3OD.

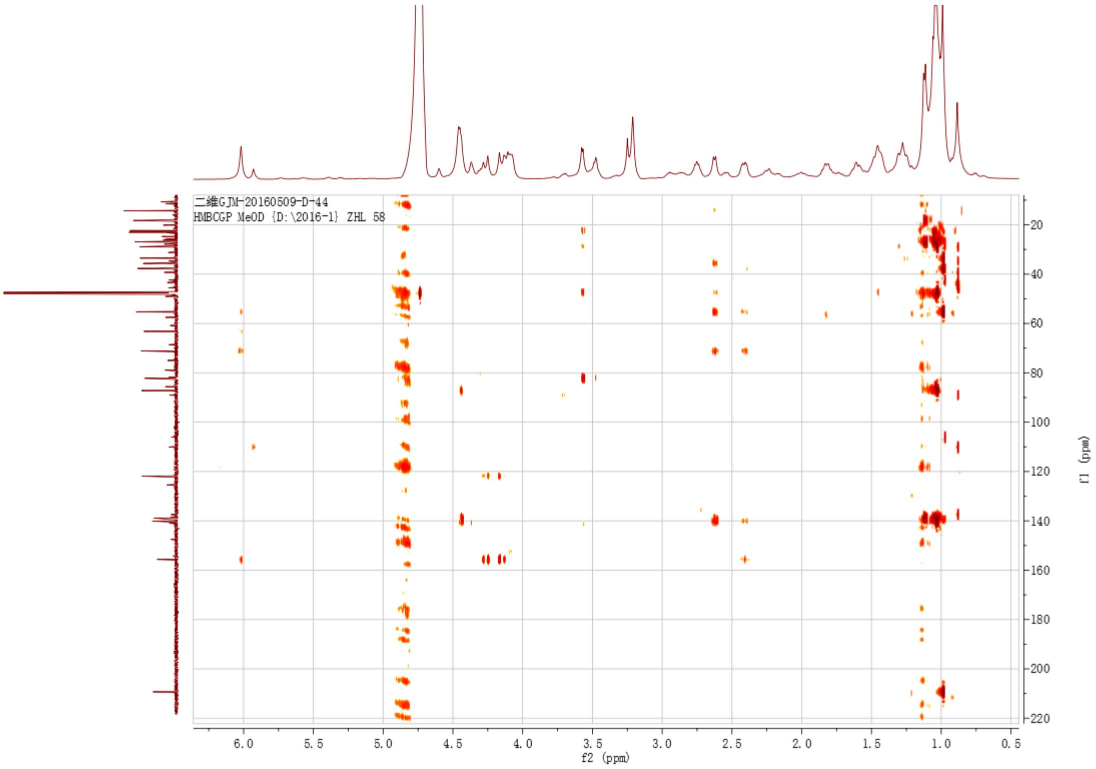

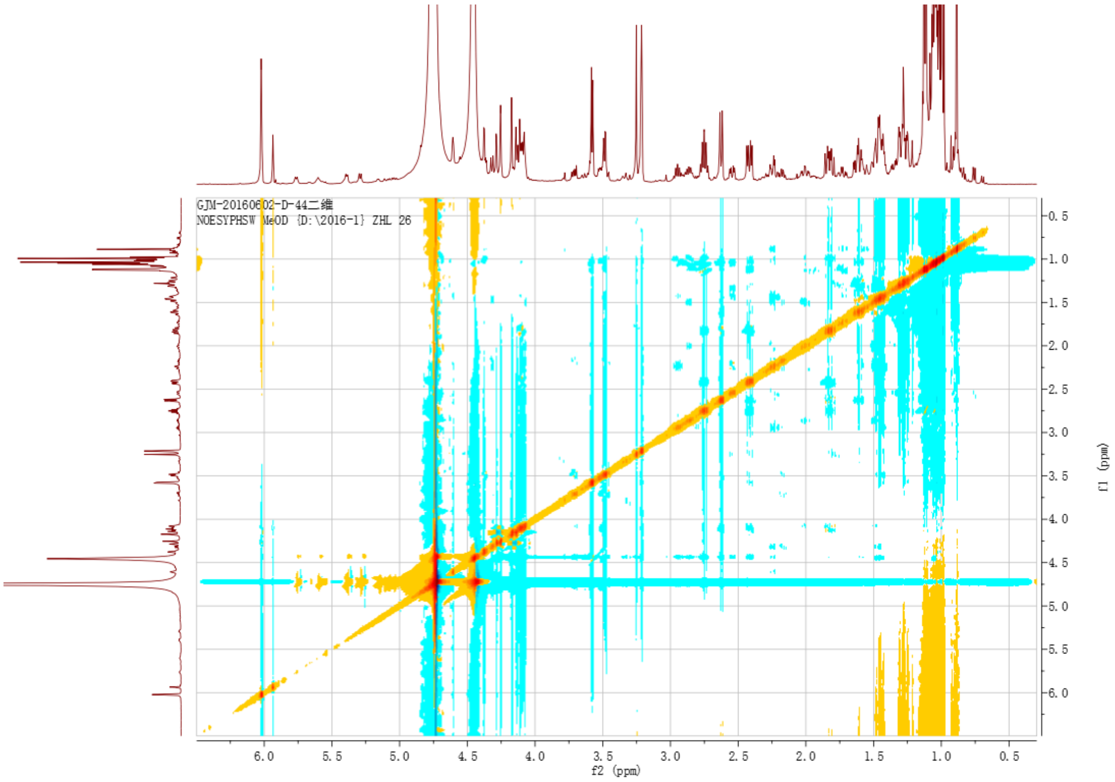

Figure S14. HRESIMS spectrum of compound **3**.

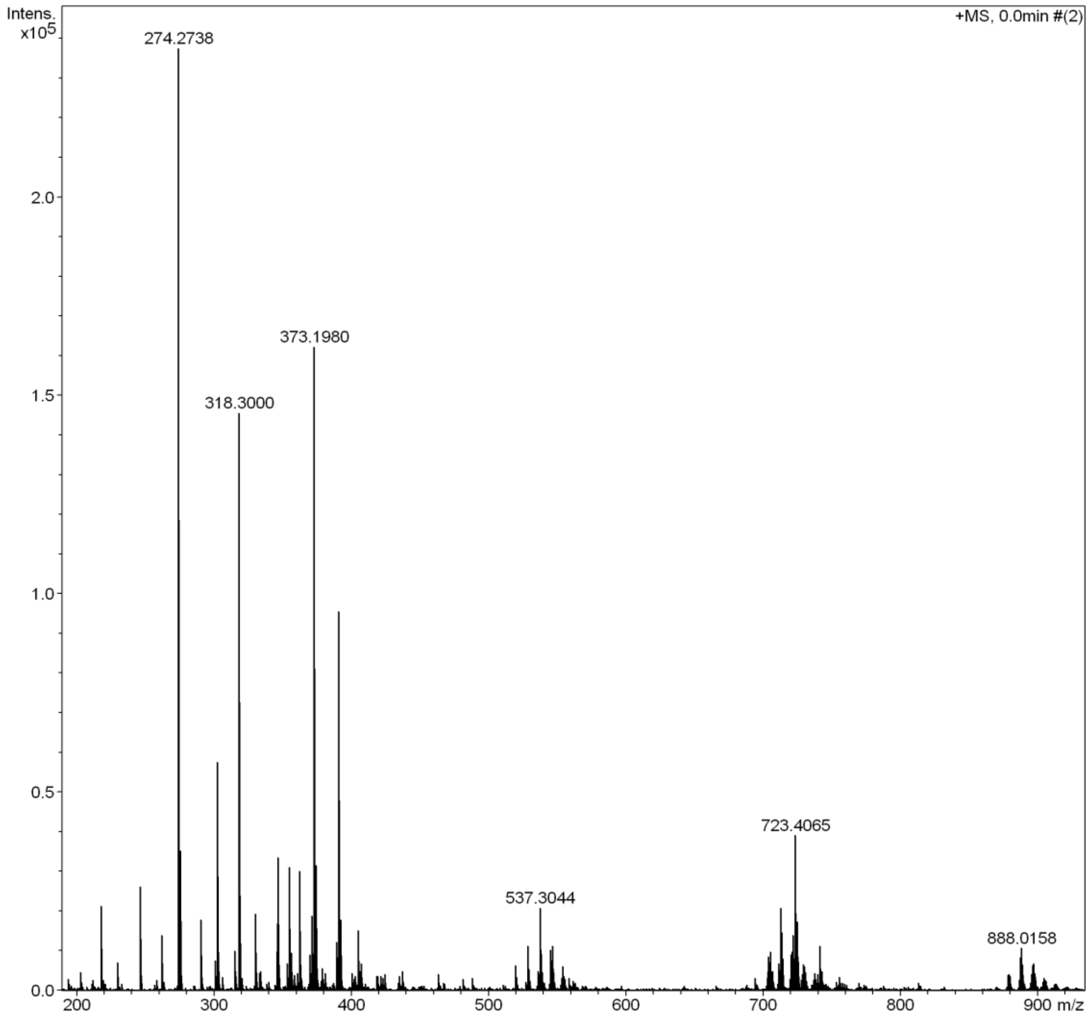

Figure S15. IR and UV spectra of compound **3**. This work is supported by Bruker (Beijing) Scientific Technology Co. Ltd.

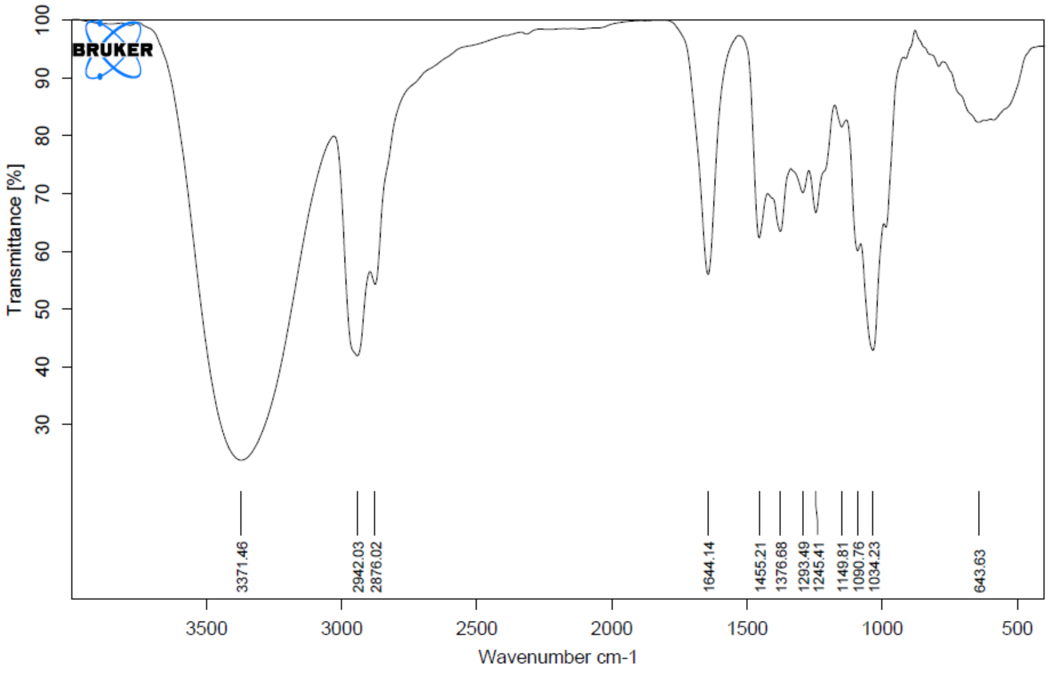

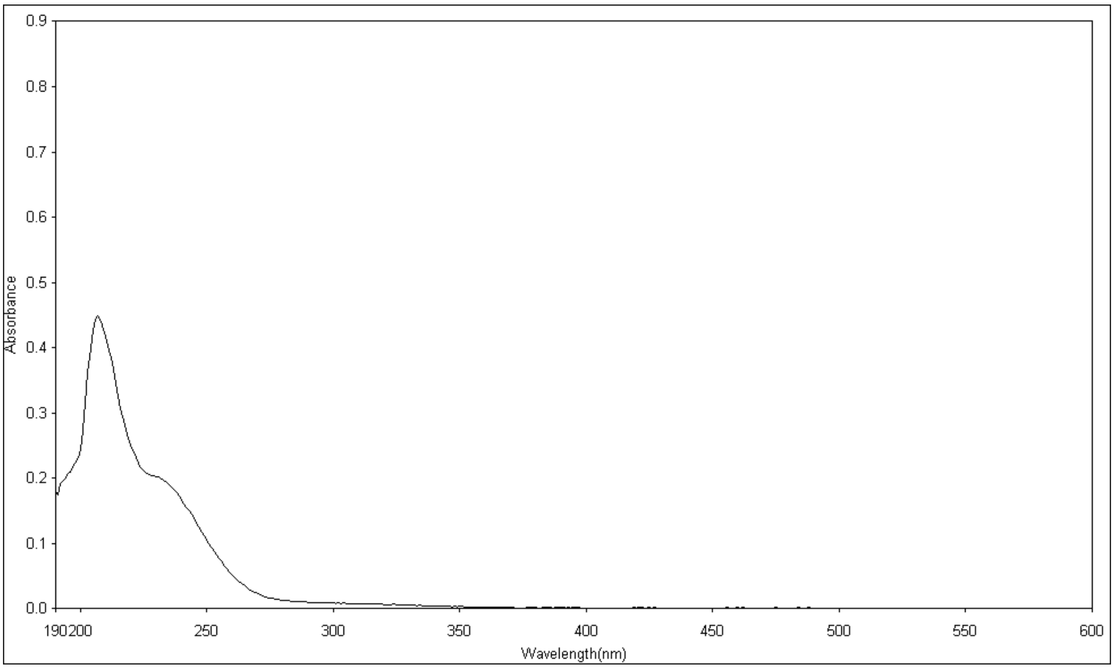

Figure S16. ^{1}H NMR and ^{13}C spectra of compound **4** in CD_3OD.

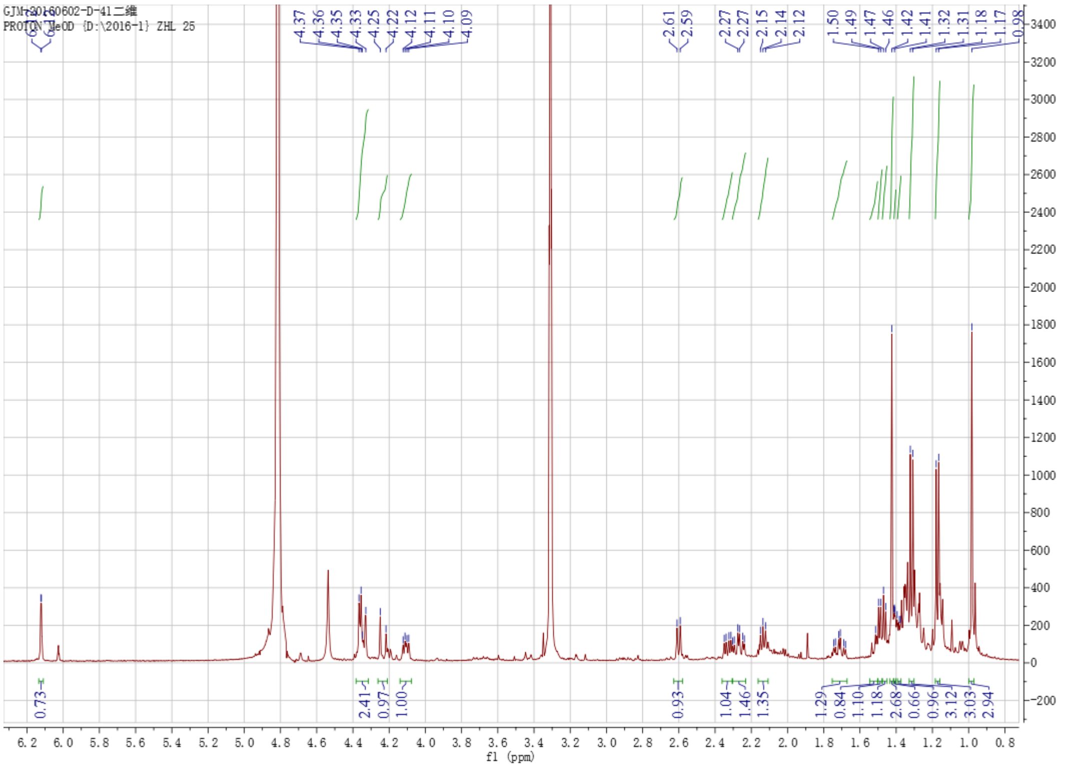

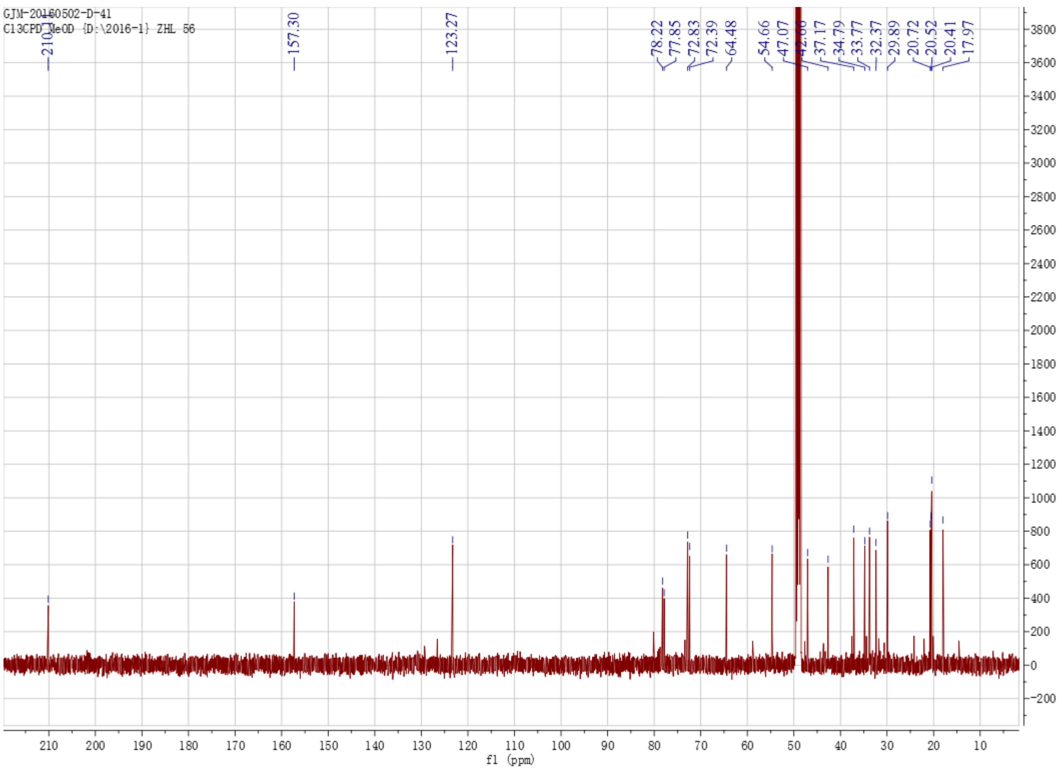

Figure S17. HSQC and ^{1}H-^{1}H COSY spectra of compound **4** in CD_3OD

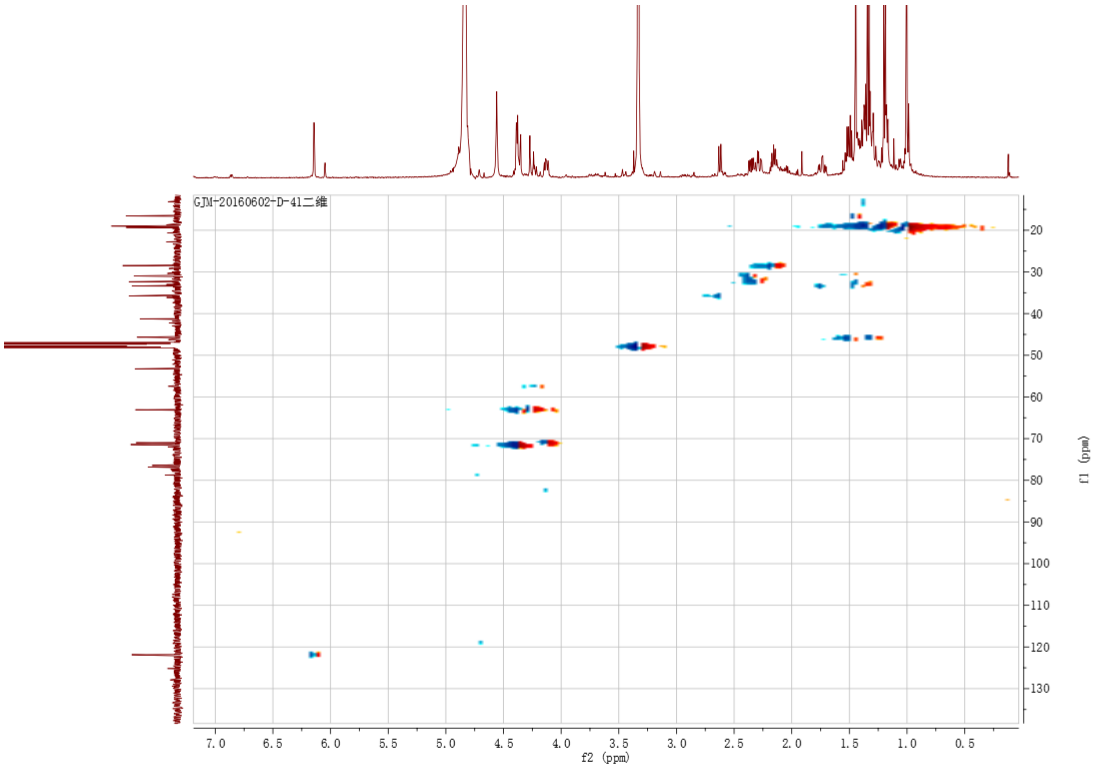

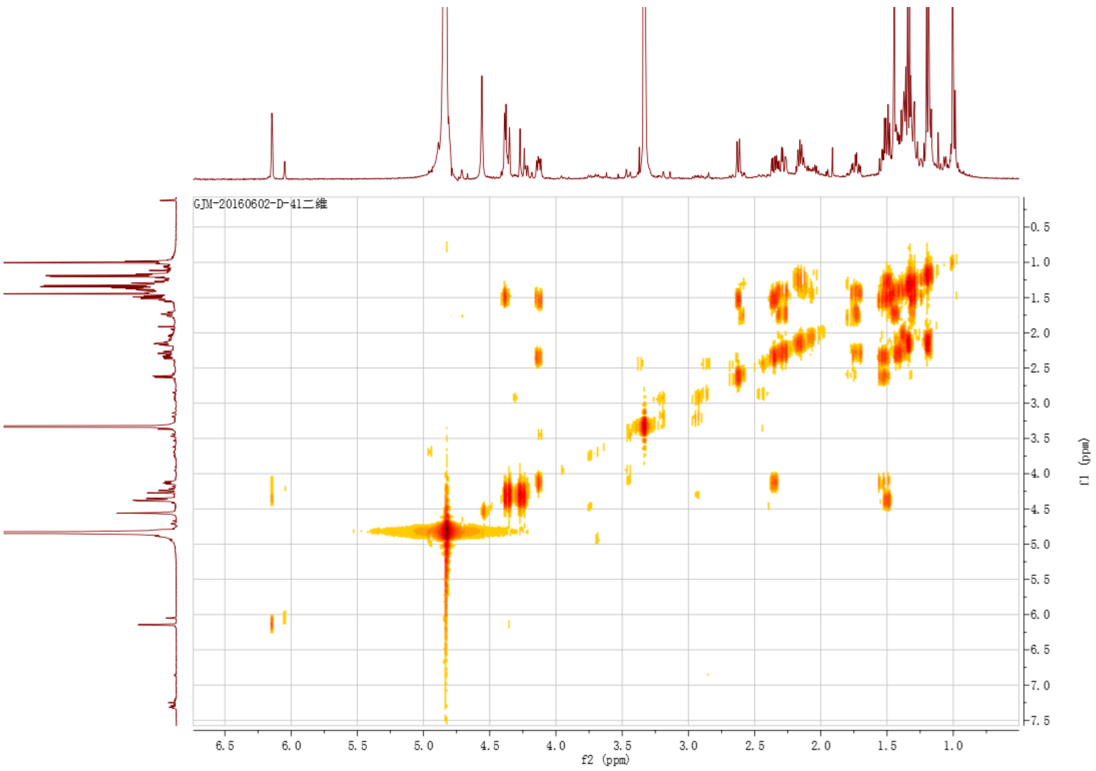

Figure S18. HMBC and NOESY spectra of compound **4** in CD_3OD.

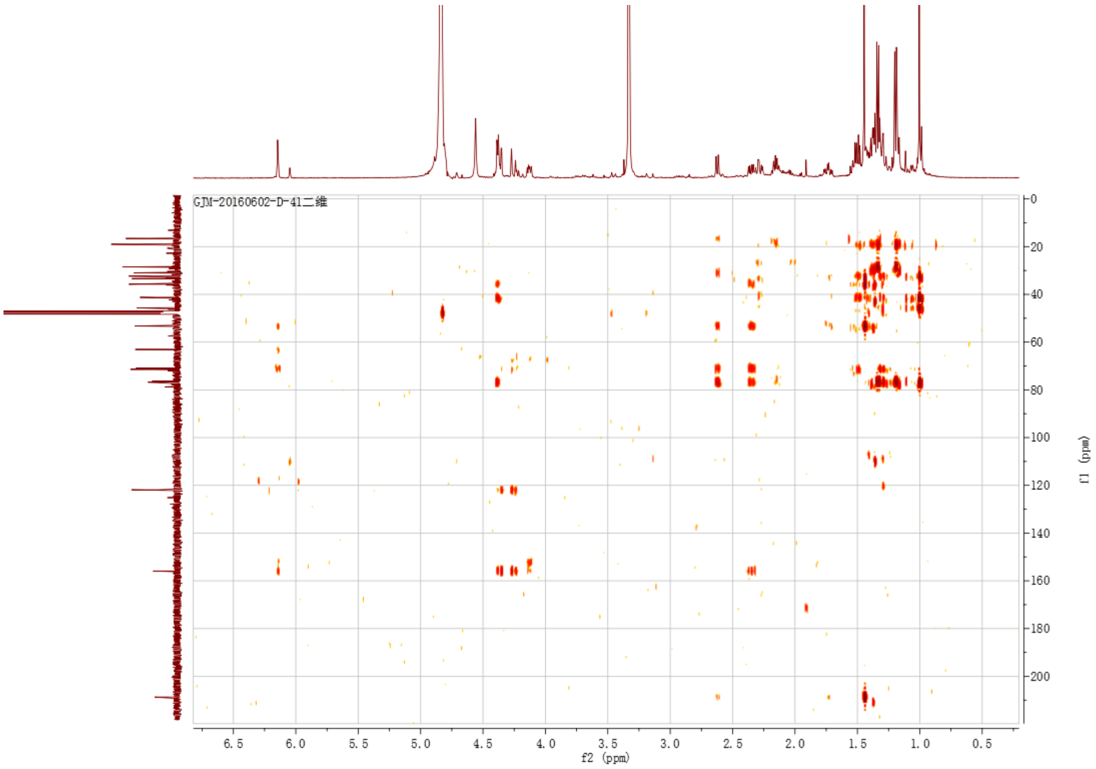

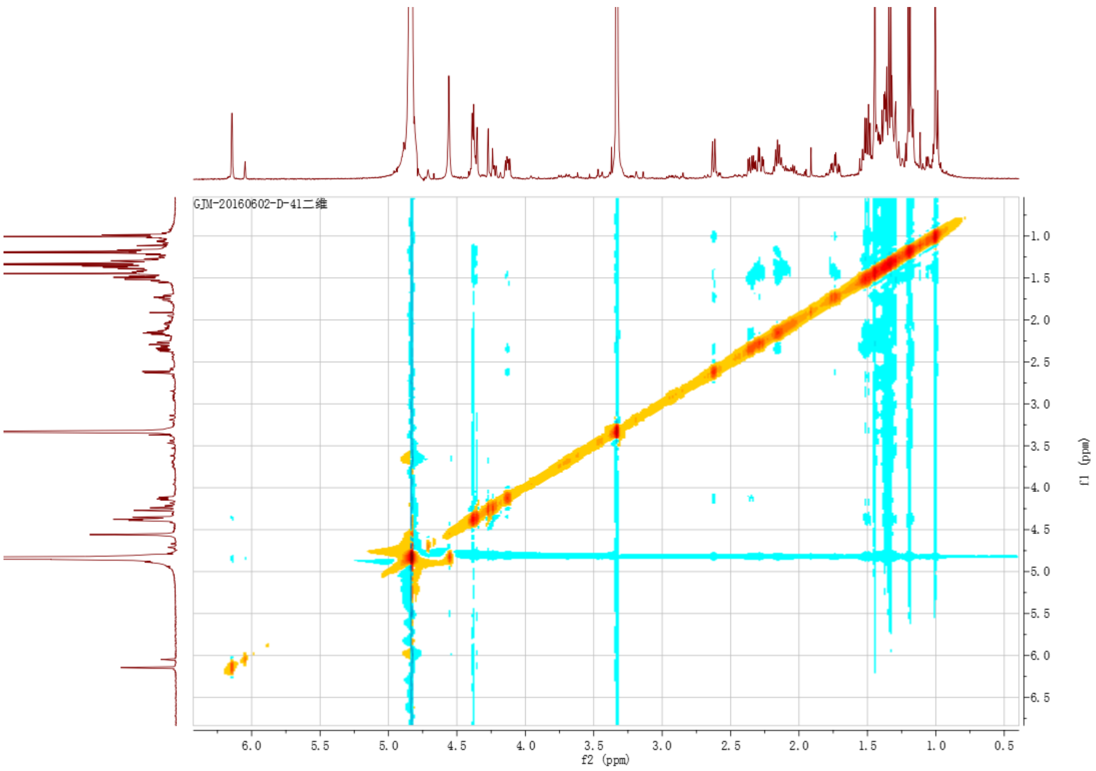

Figure S19. HRESIMS spectrum of compound **4**.

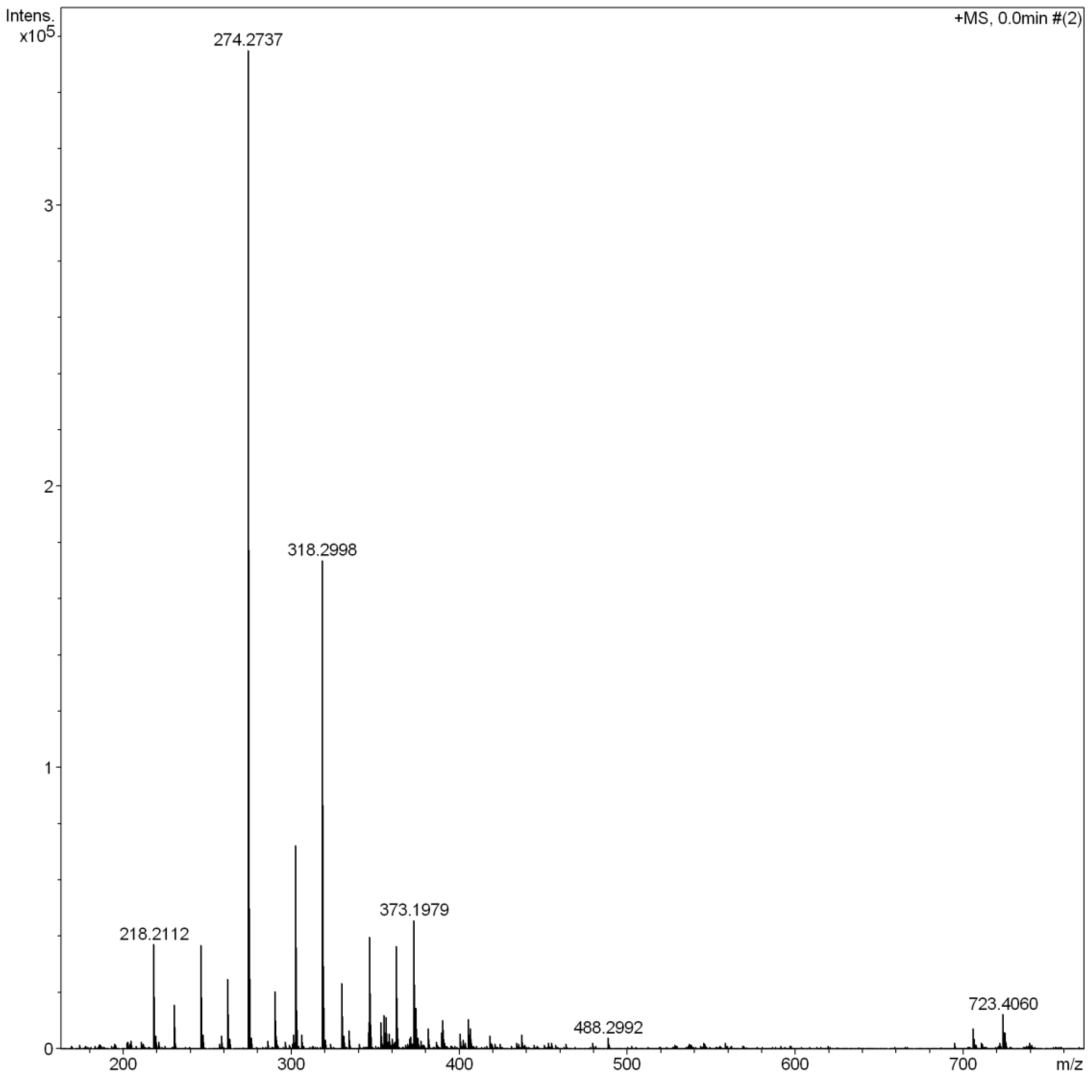

Figure S20. IR and UV spectra of compound **4**. This work is supported by Bruker (Beijing) Scientific Technology Co. Ltd.

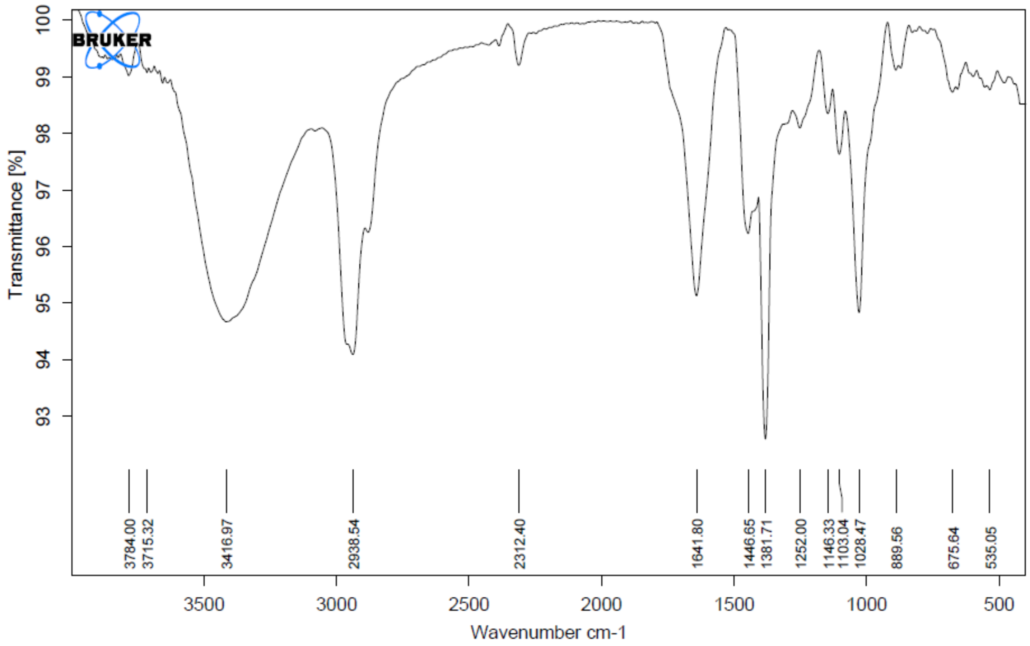

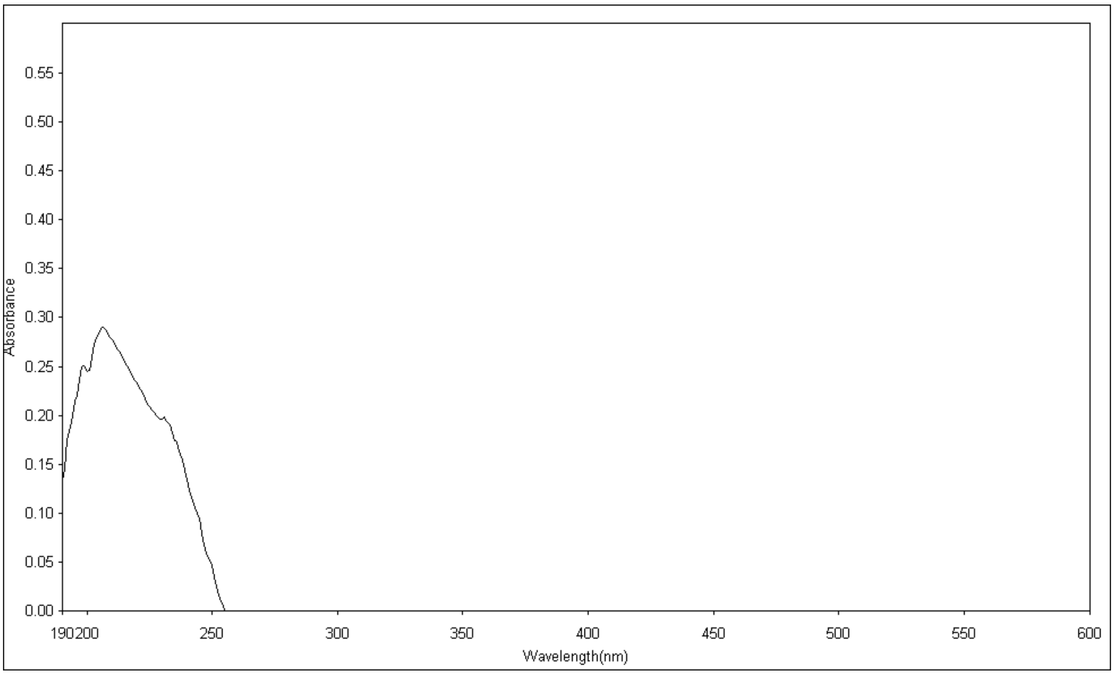

Figure S21. ^{1}H NMR and ^{13}C spectra of compound **5** in CD_3OD.

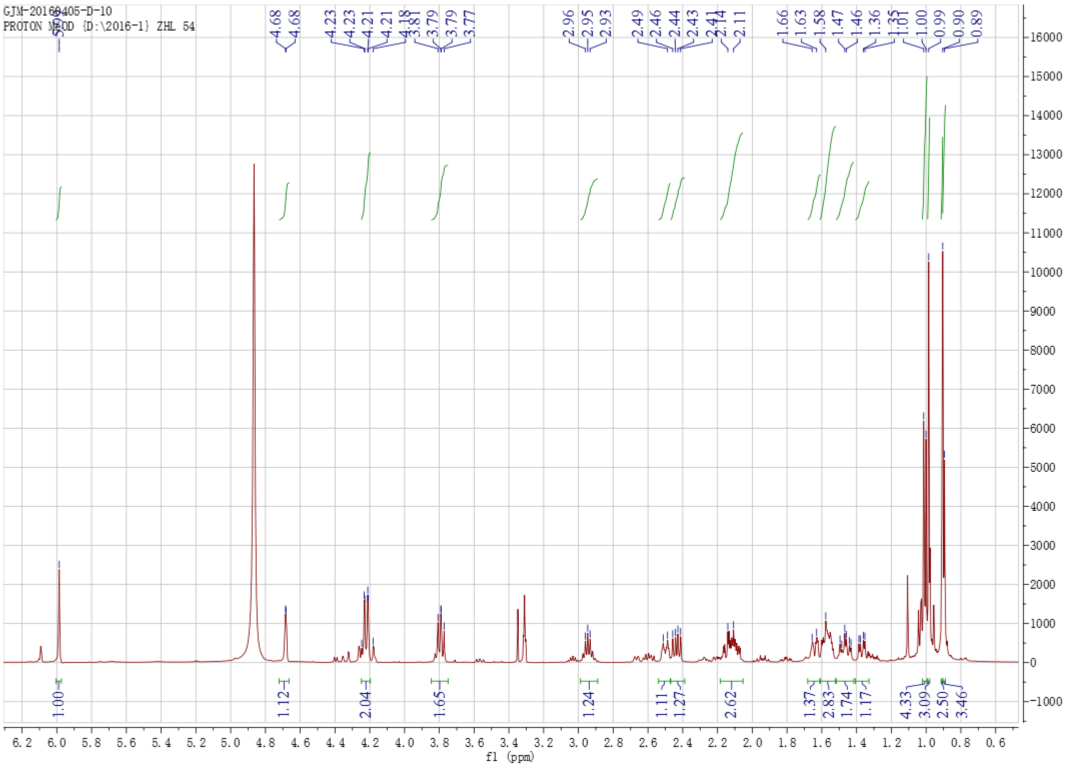

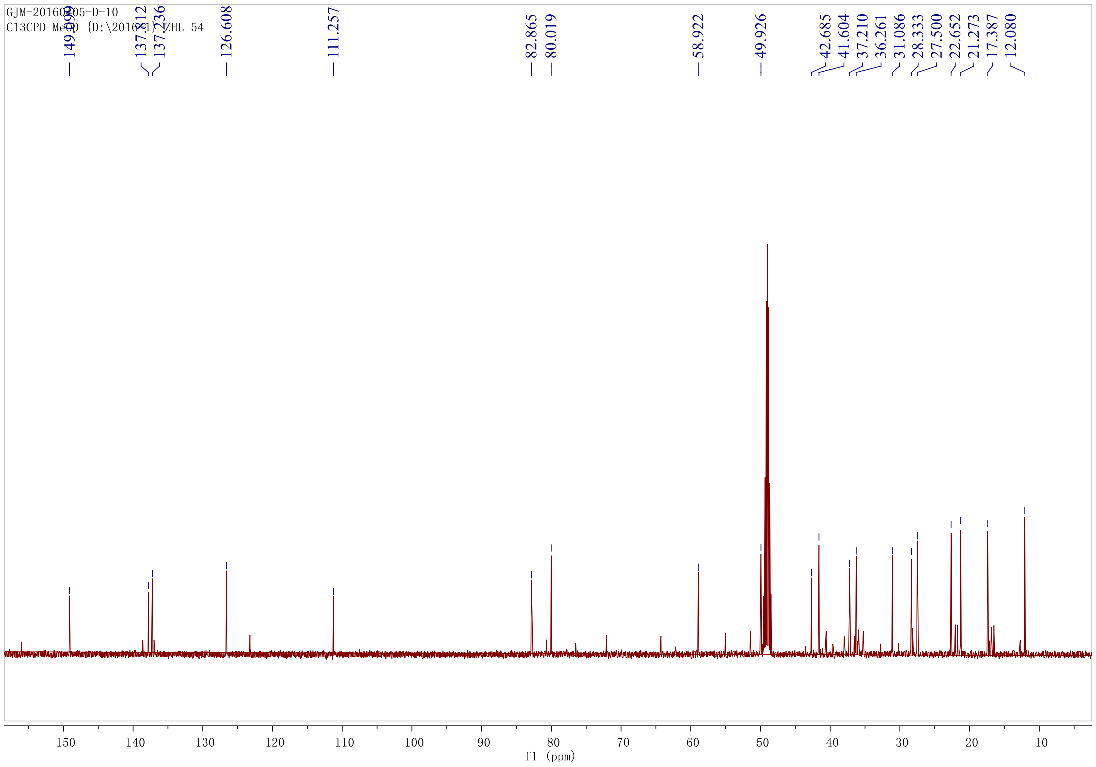

Figure S22. HSQC and ^{1}H-^{1}H COSY spectra of compound **5** in CD_3OD

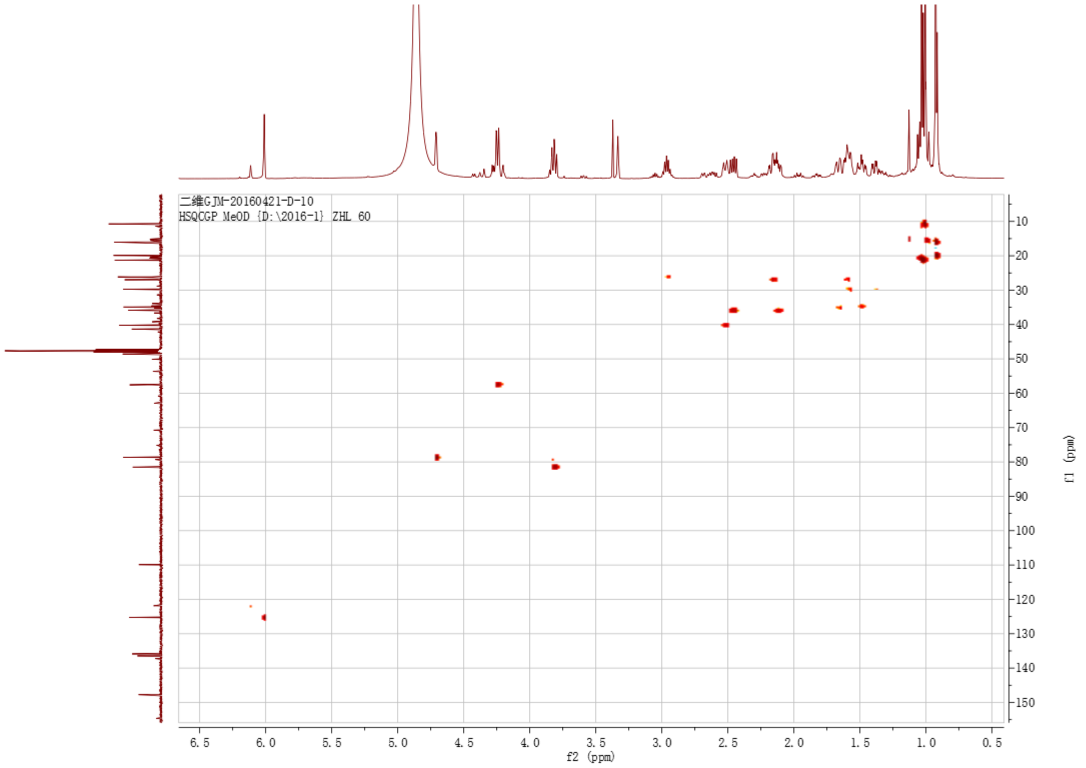

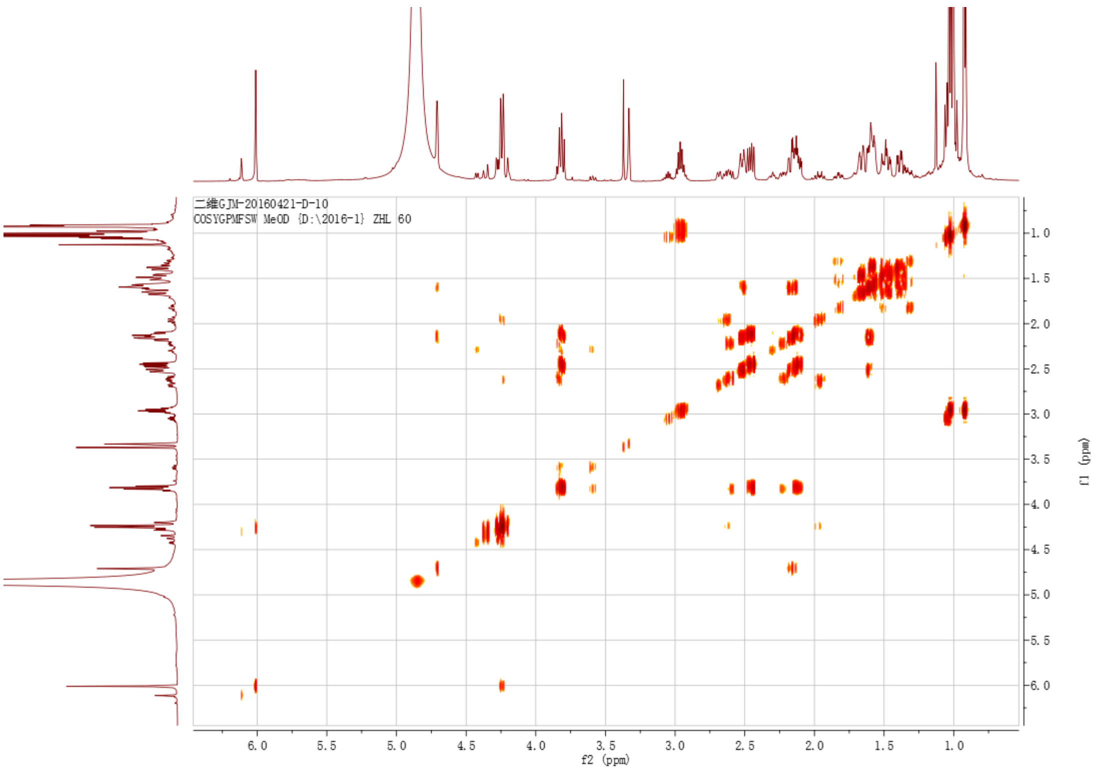

Figure S23. HMBC and NOESY spectra of compound **5** in CD_3OD.

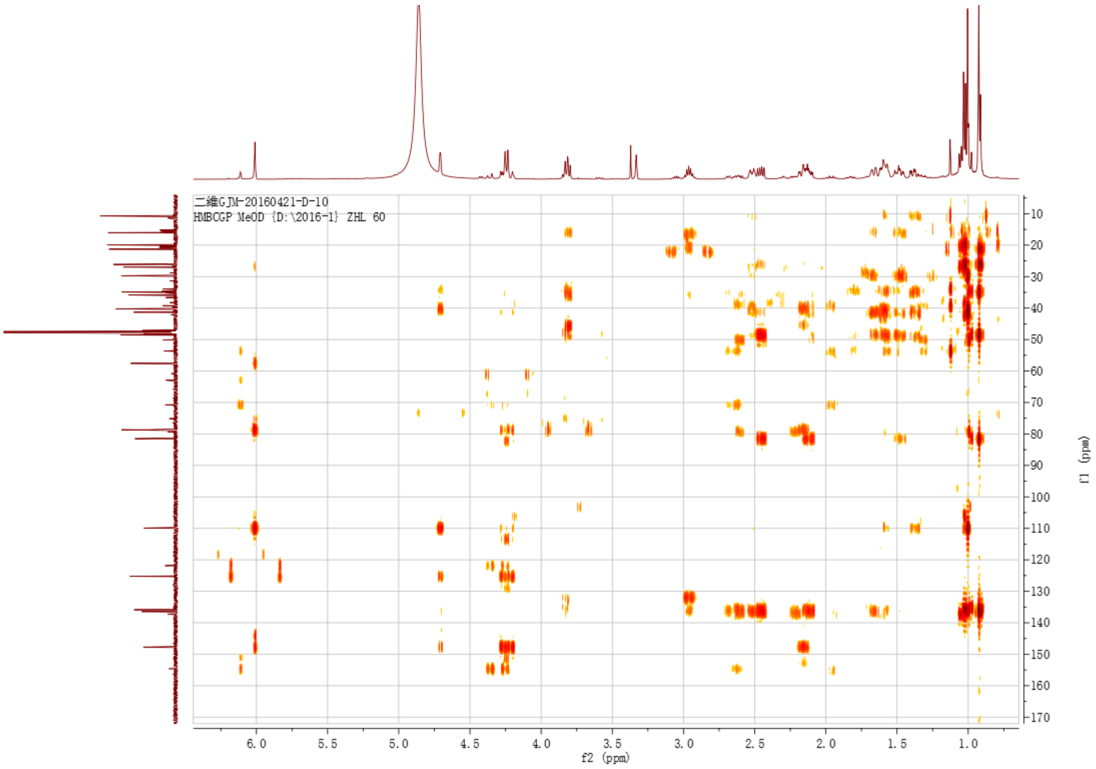

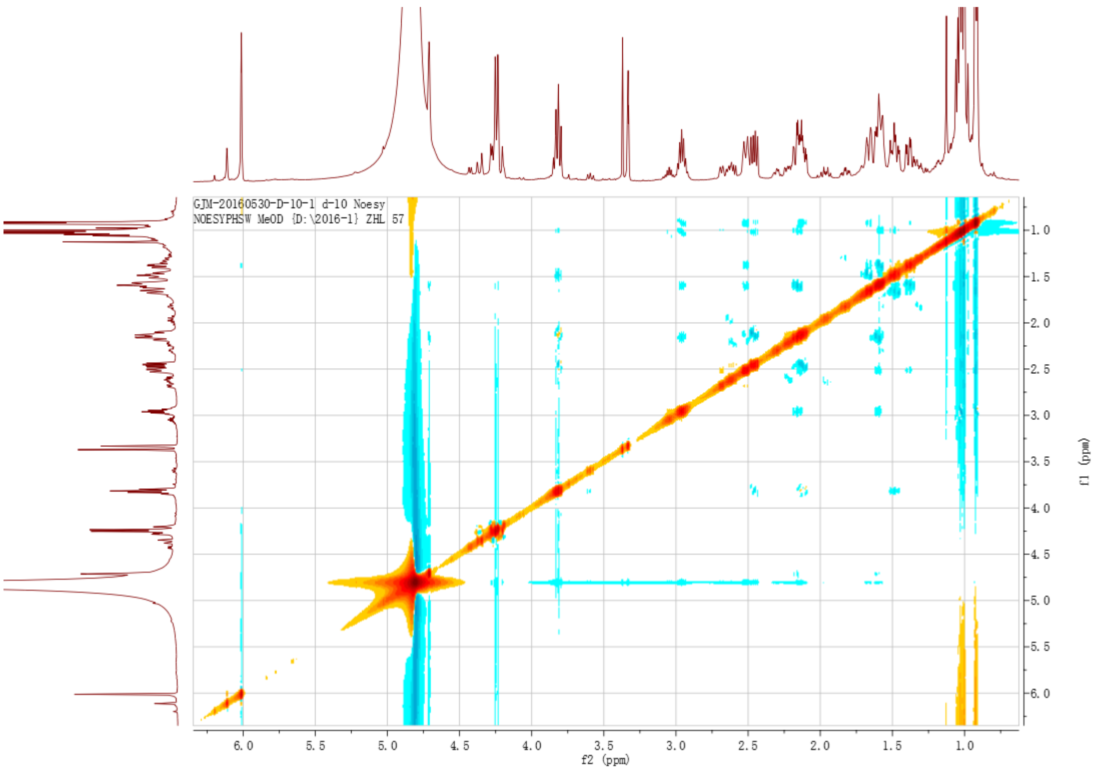

Figure S24. HRESIMS spectrum of compound **5**.

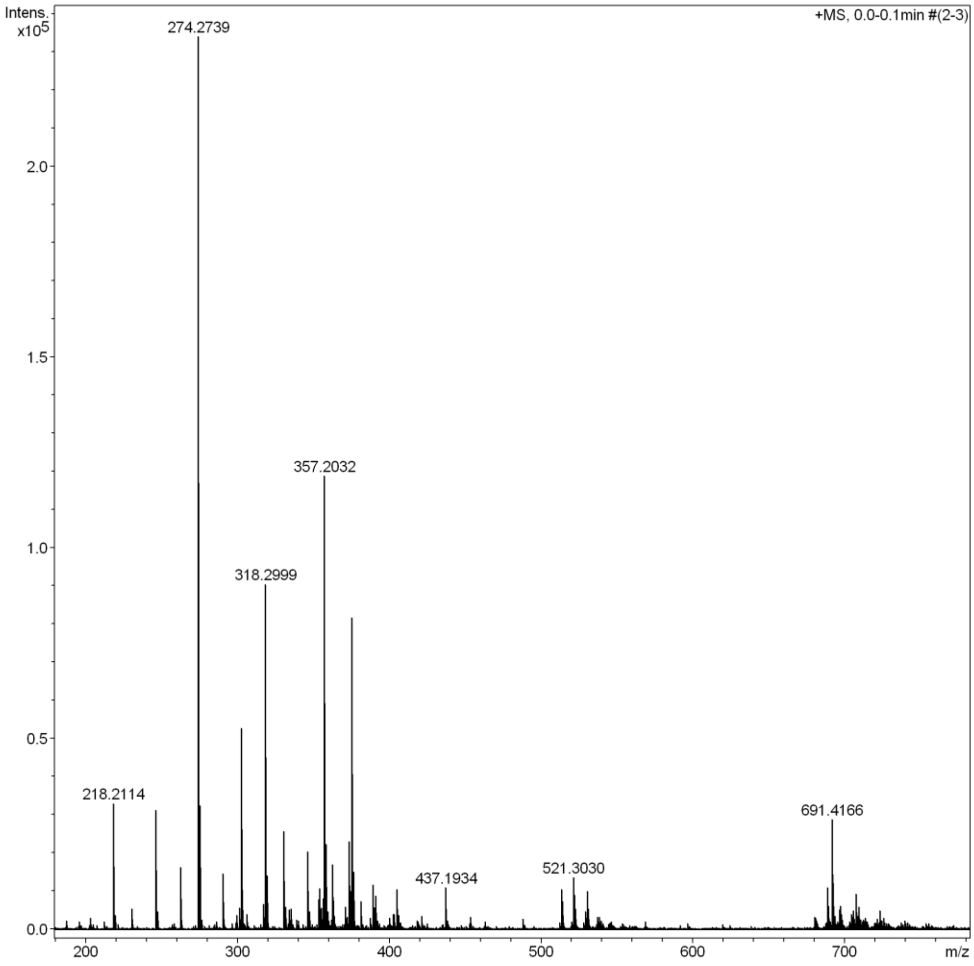

Figure S25. IR and UV spectra of compound **5**. This work is supported by Bruker (Beijing) Scientific Technology Co. Ltd.

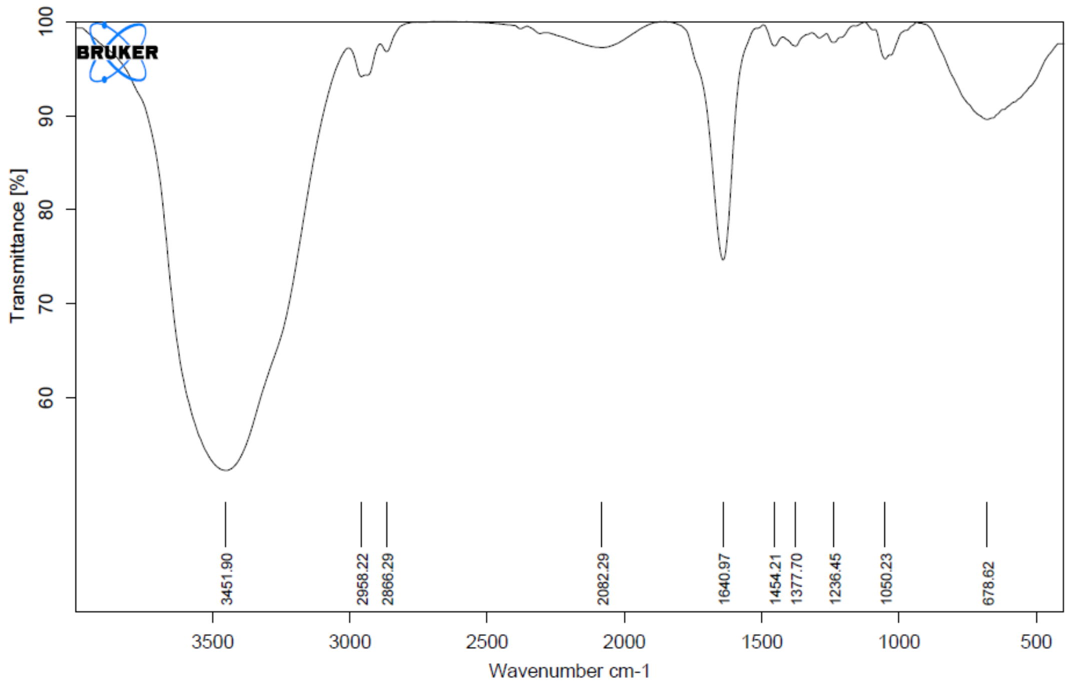

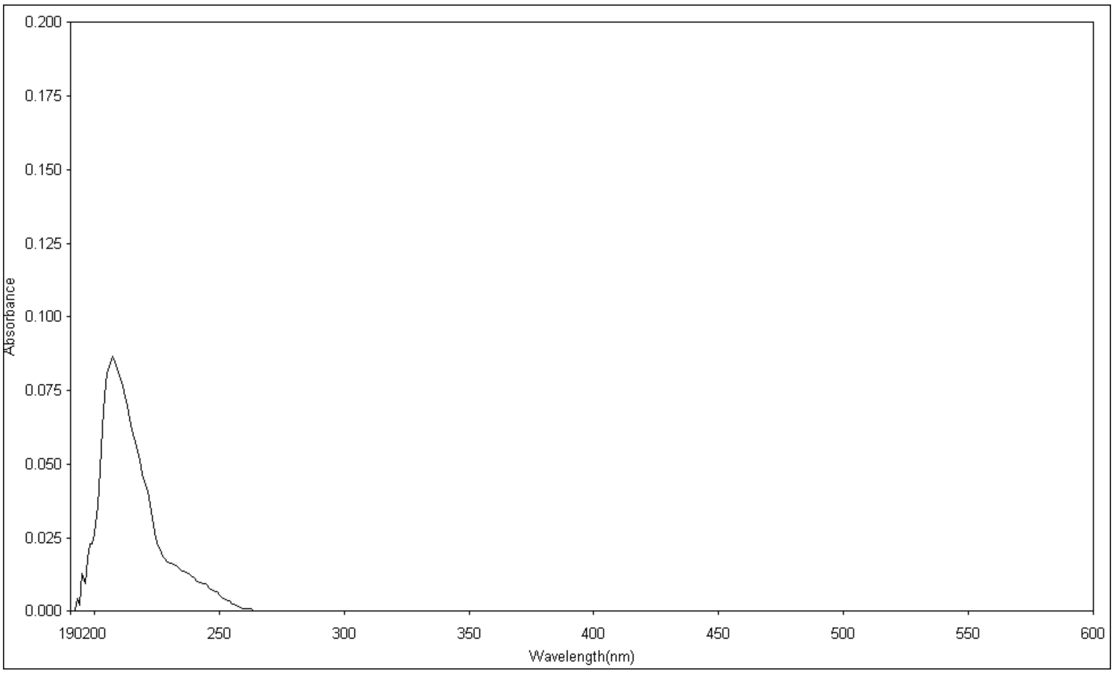

Figure S26. ^{1}H NMR and ^{13}C spectra of compound **6** in CD_3OD.

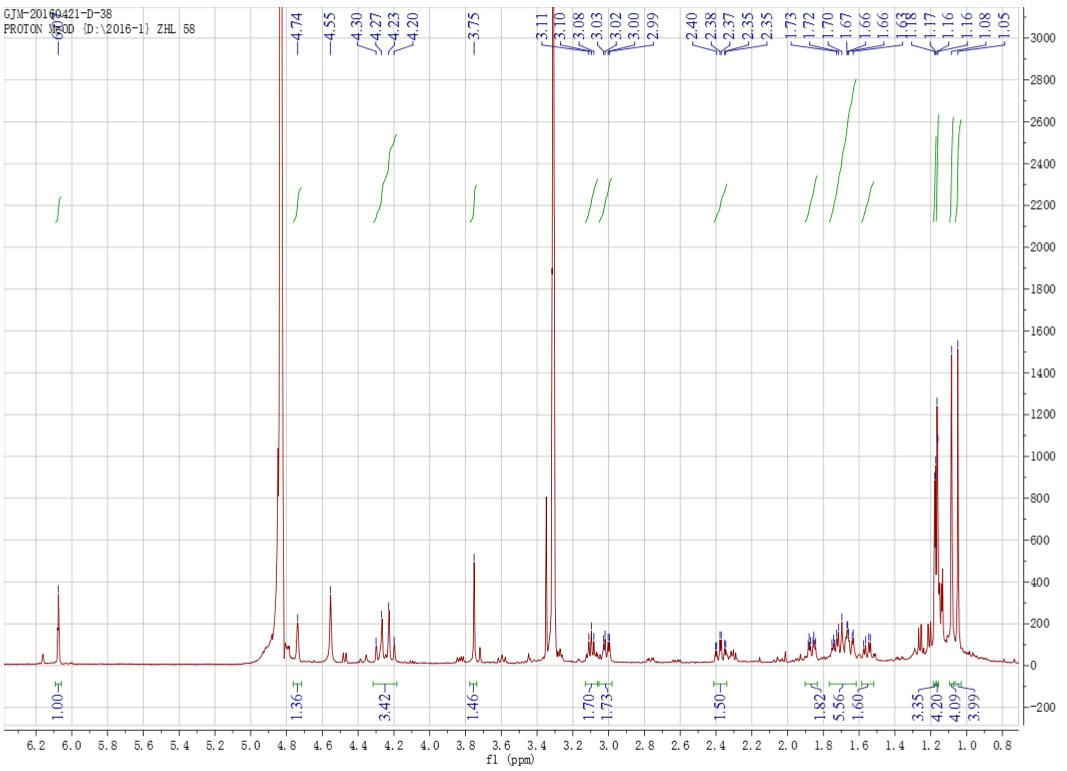

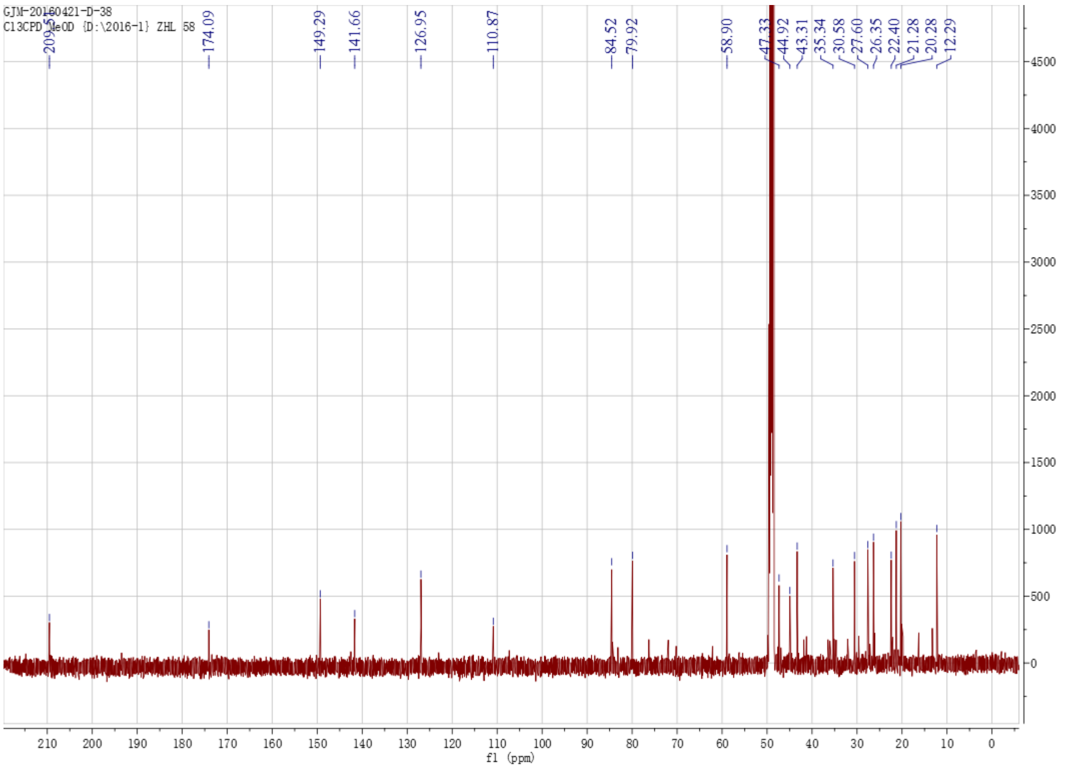

Figure S27. HSQC and ^{1}H-^{1}H COSY spectra of compound **6** in CD_3OD

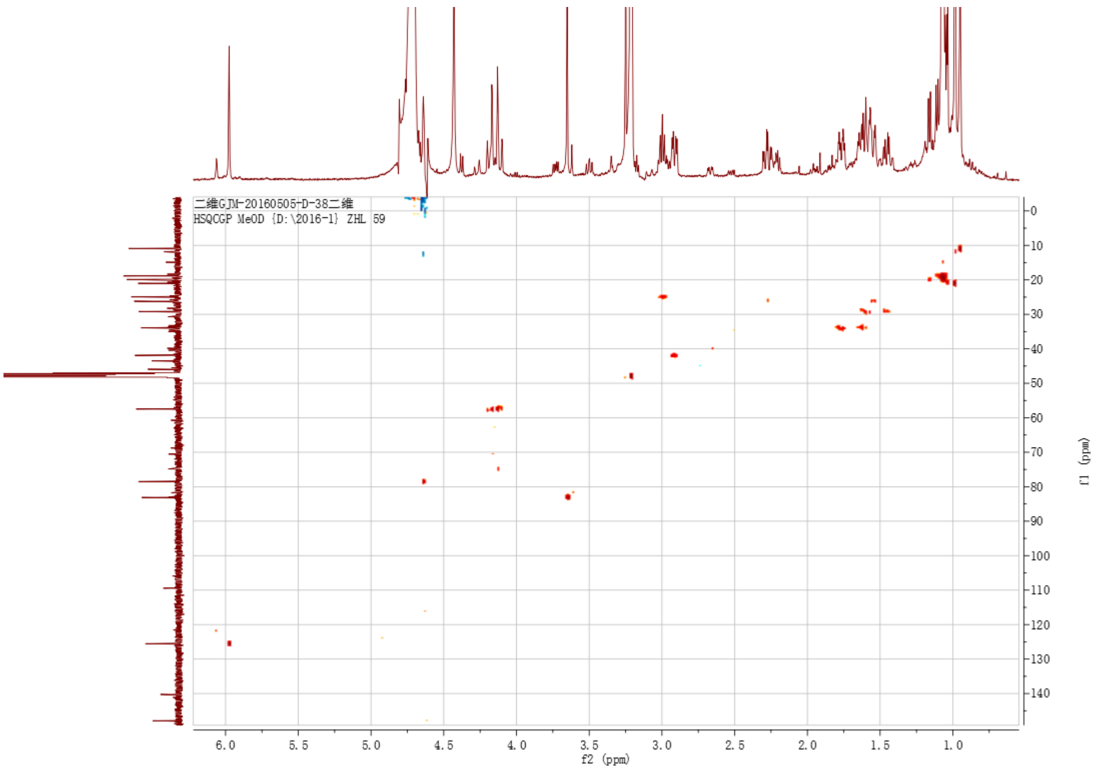

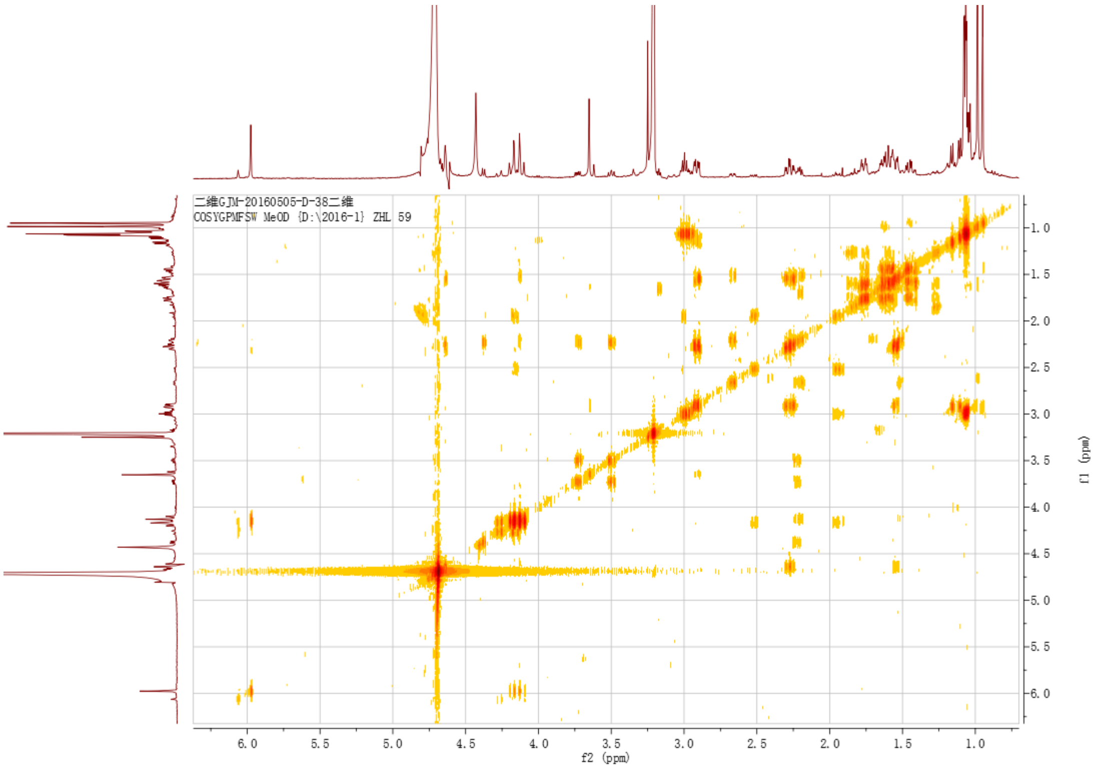

Figure S28. HMBC and NOESY spectra of compound **6** in CD_3OD.

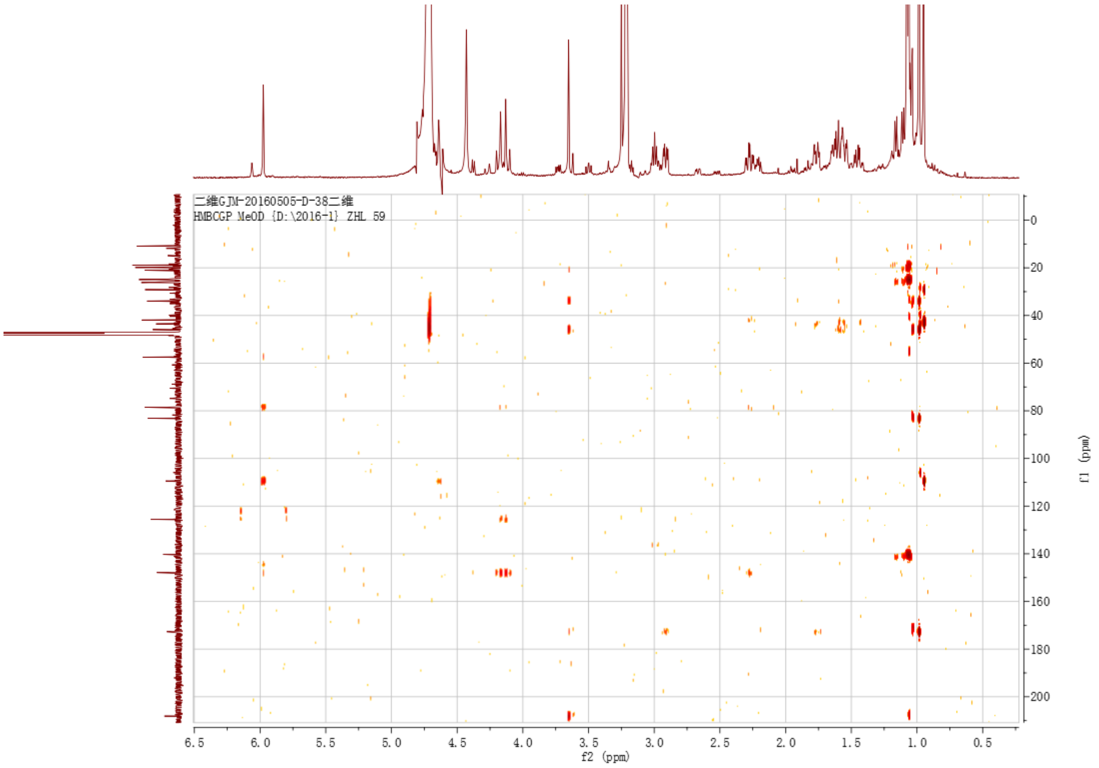

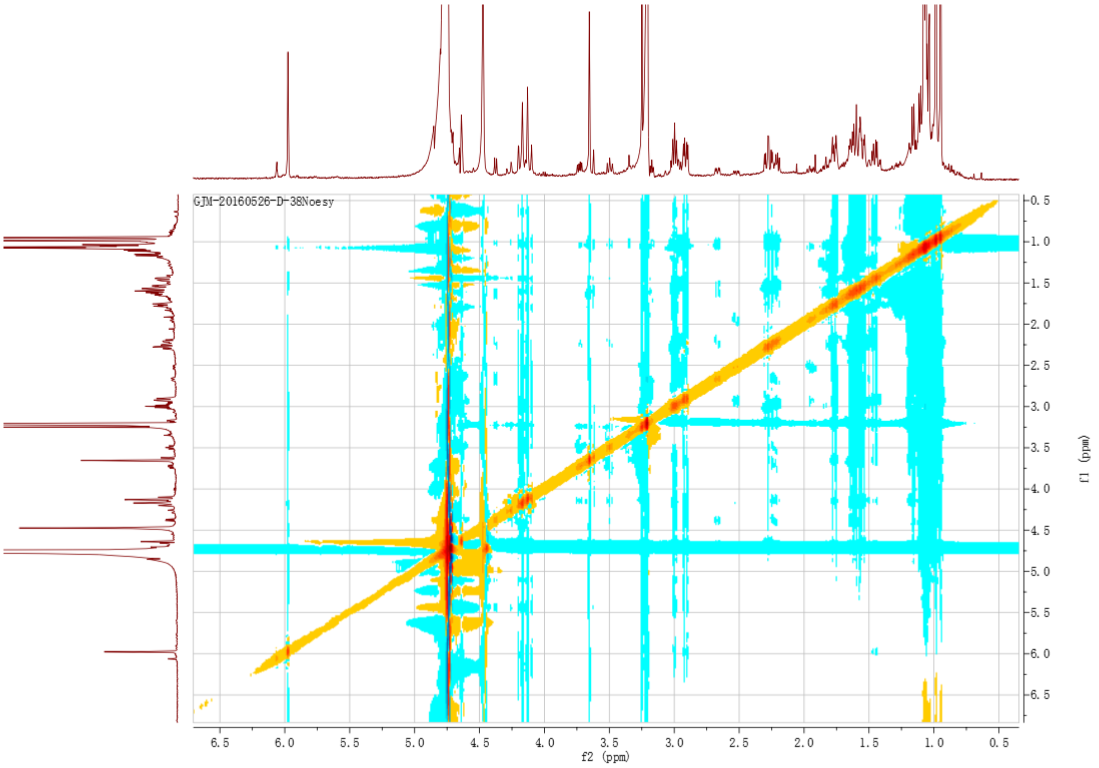

Figure S29. HRESIMS spectrum of compound **6**.

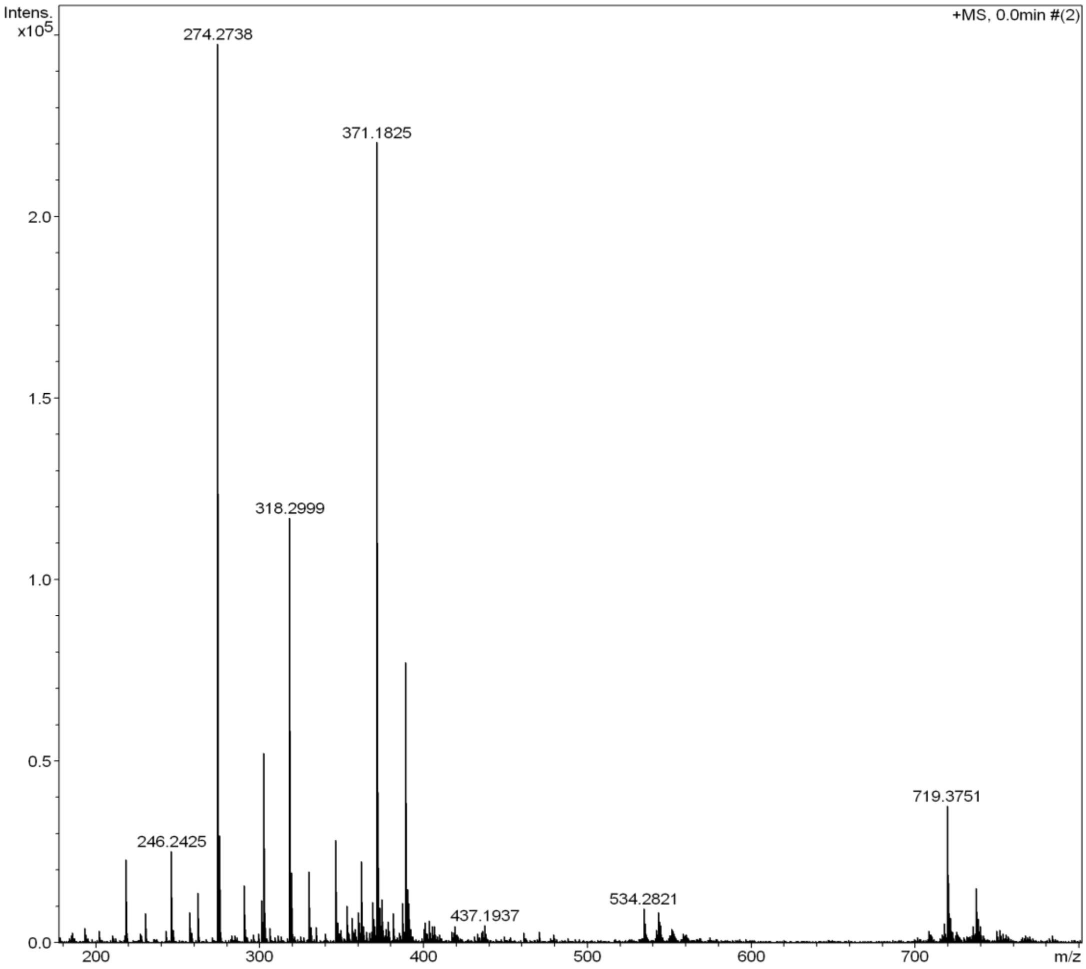

Figure S30. IR and UV spectra of compound **6**. This work is supported by Bruker (Beijing) Scientific Technology Co. Ltd.

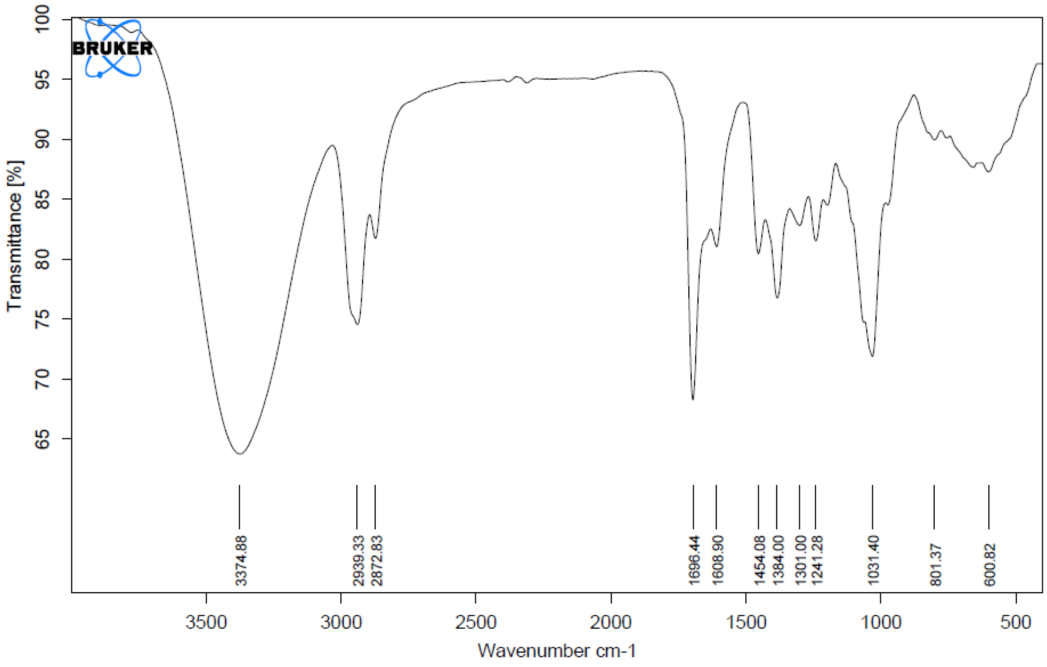

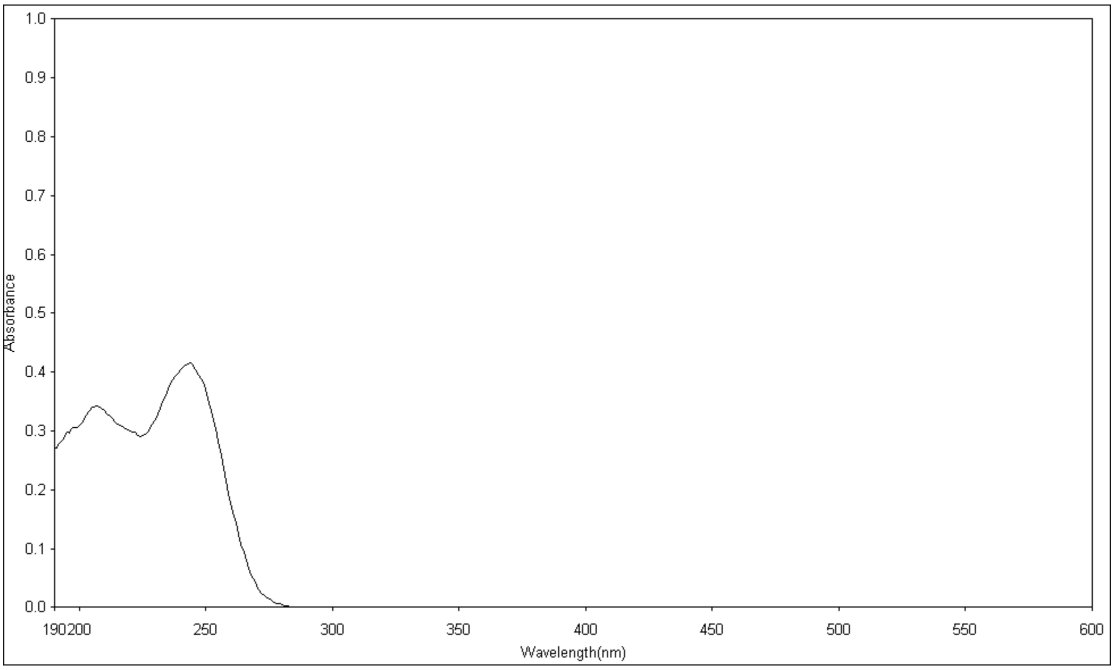

Figure S31. ^{1}H NMR and ^{13}C spectra of compound **7** in CD_3OD.

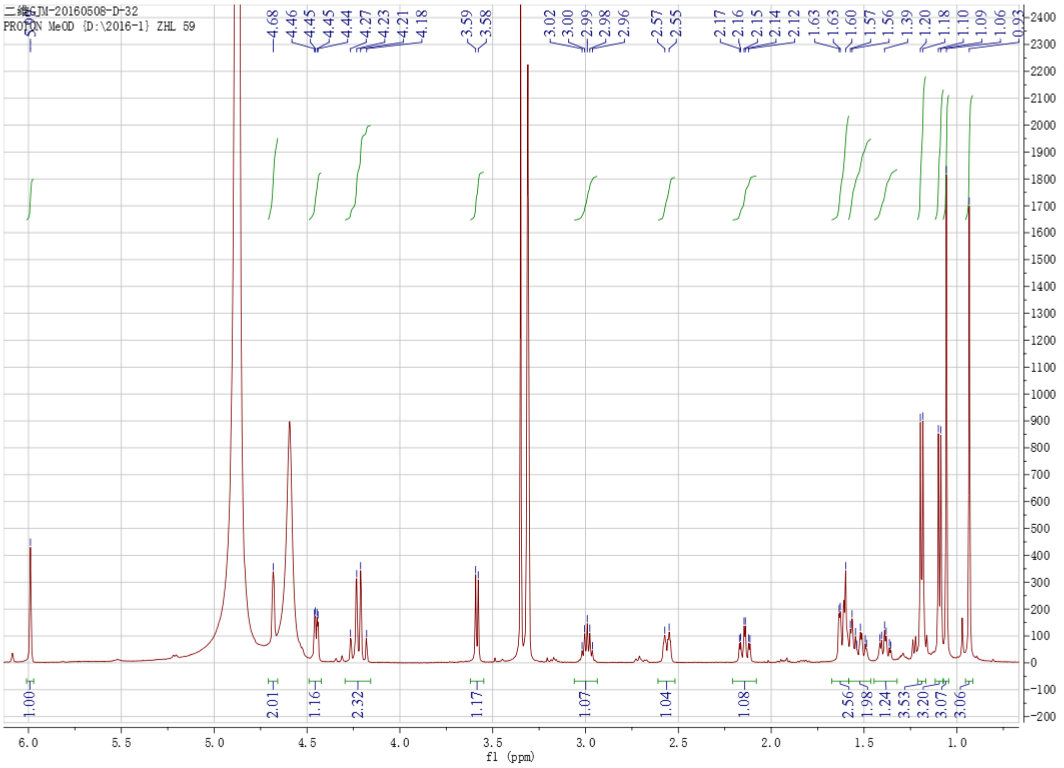

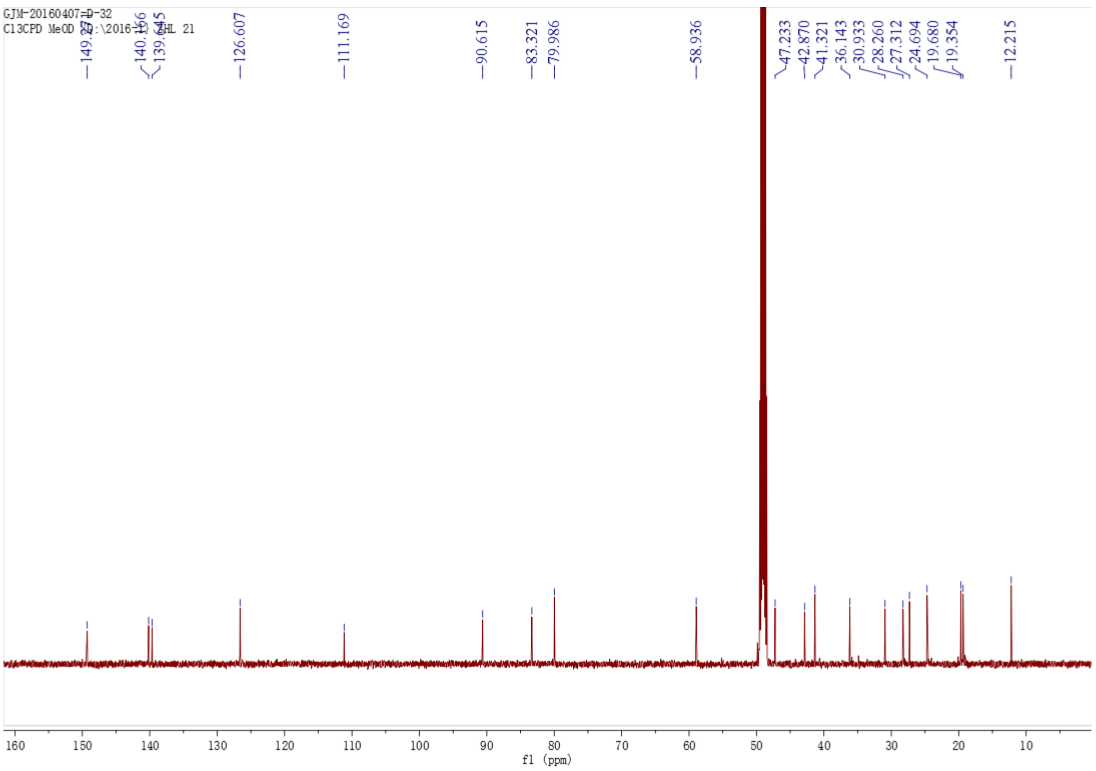

Figure S32. HSQC and ^{1}H-^{1}H COSY spectra of compound **7** in CD_3OD

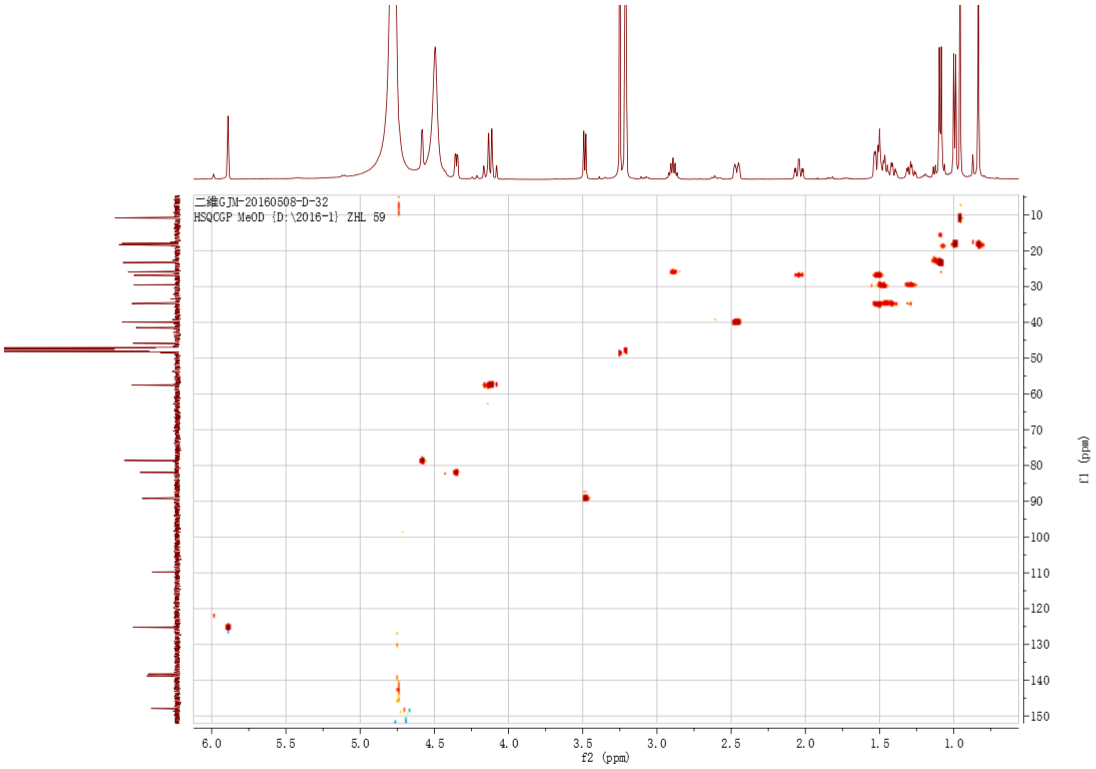

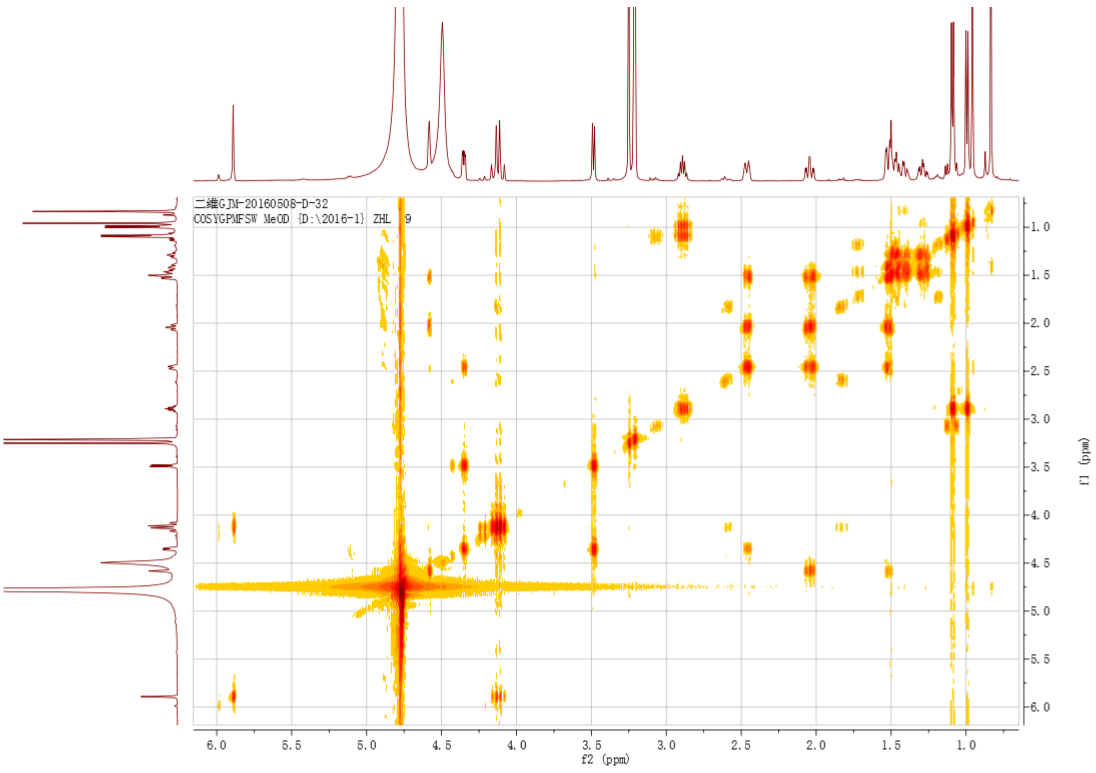

Figure S33. HMBC and NOESY spectra of compound **7** in CD_3OD.

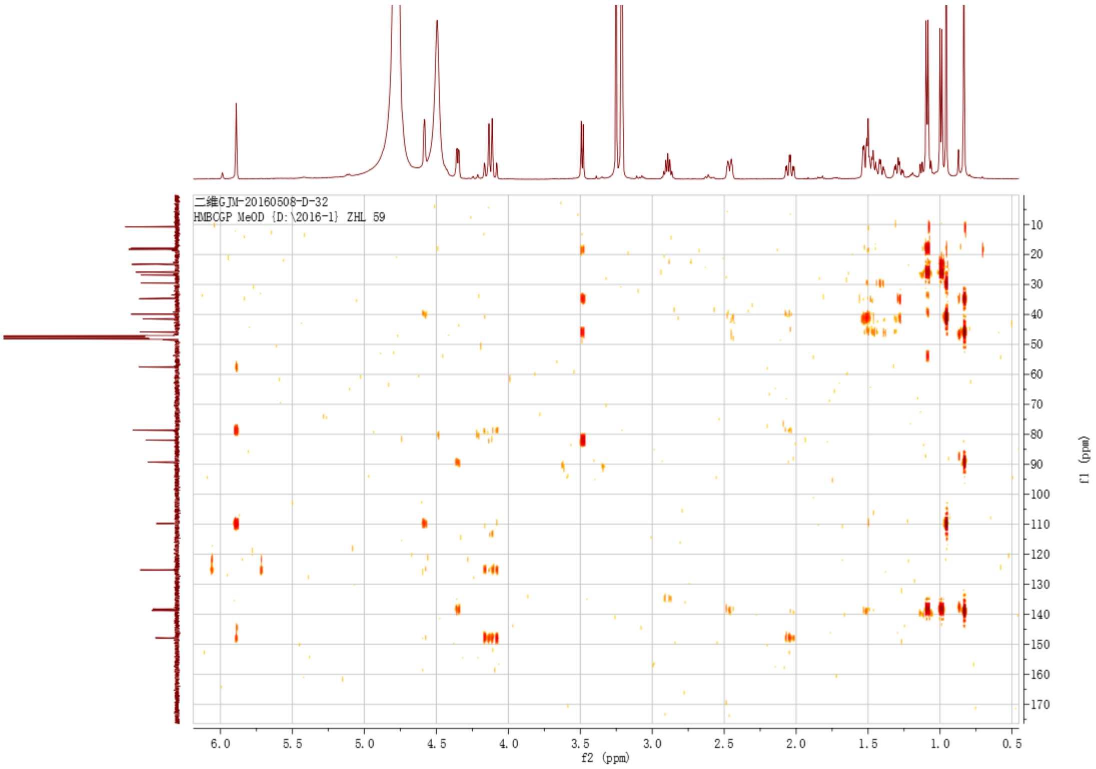

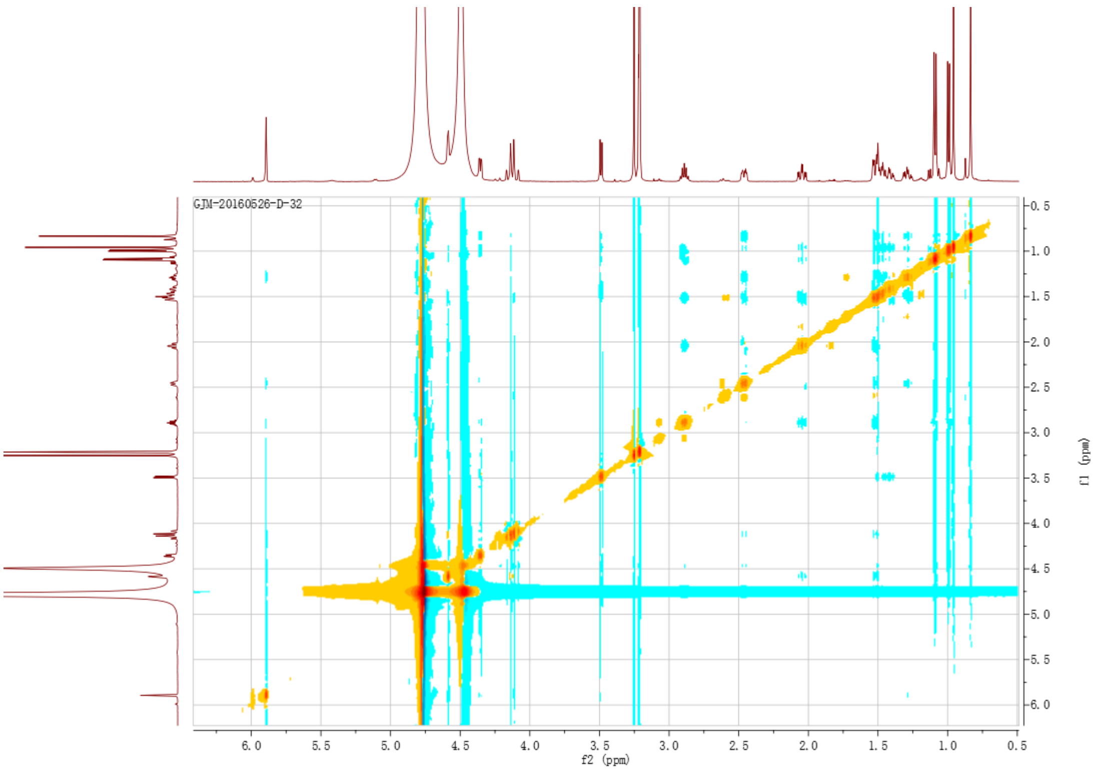

Figure S34. HRESIMS spectrum of compound **7**.

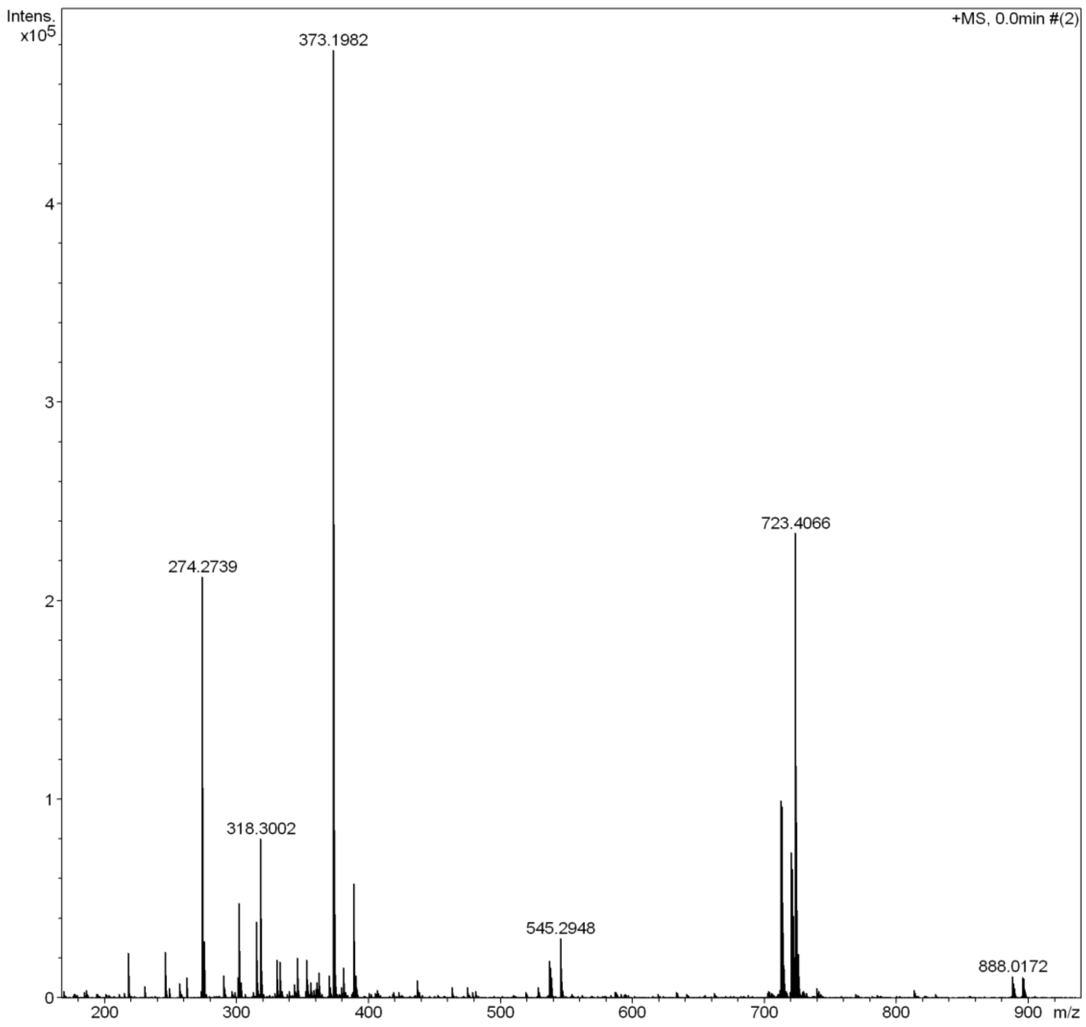

Figure S35. IR and UV spectra of compound **7**. This work is supported by Bruker (Beijing) Scientific Technology Co. Ltd.

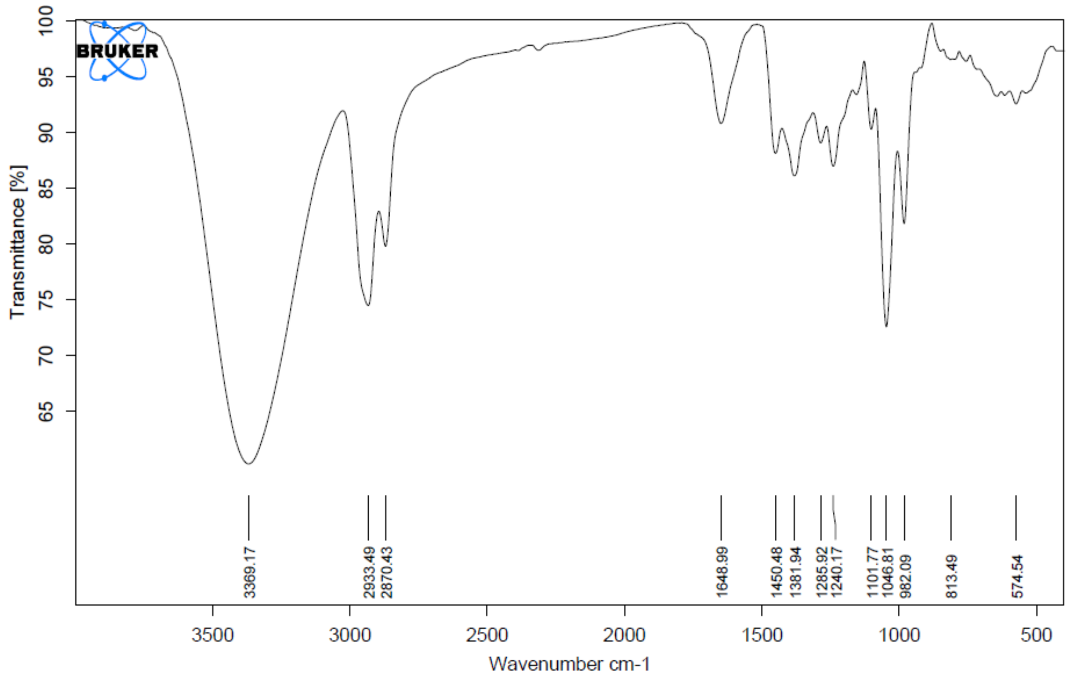

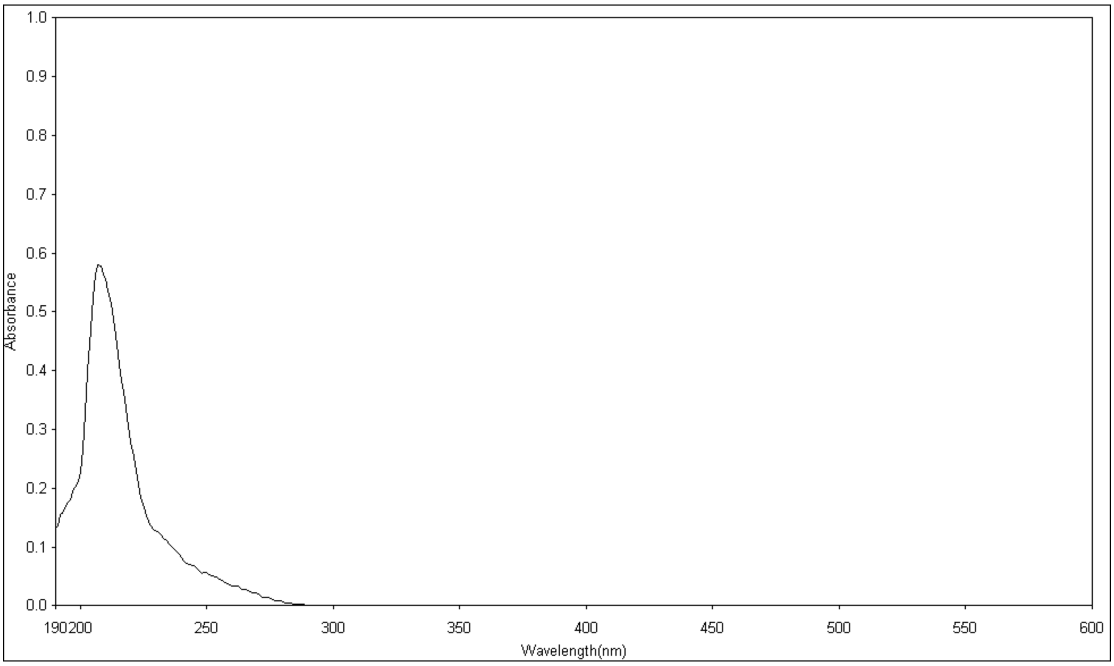

Figure S36. ^{1}H NMR and ^{13}C spectra of compound **8** in CD_3OD.

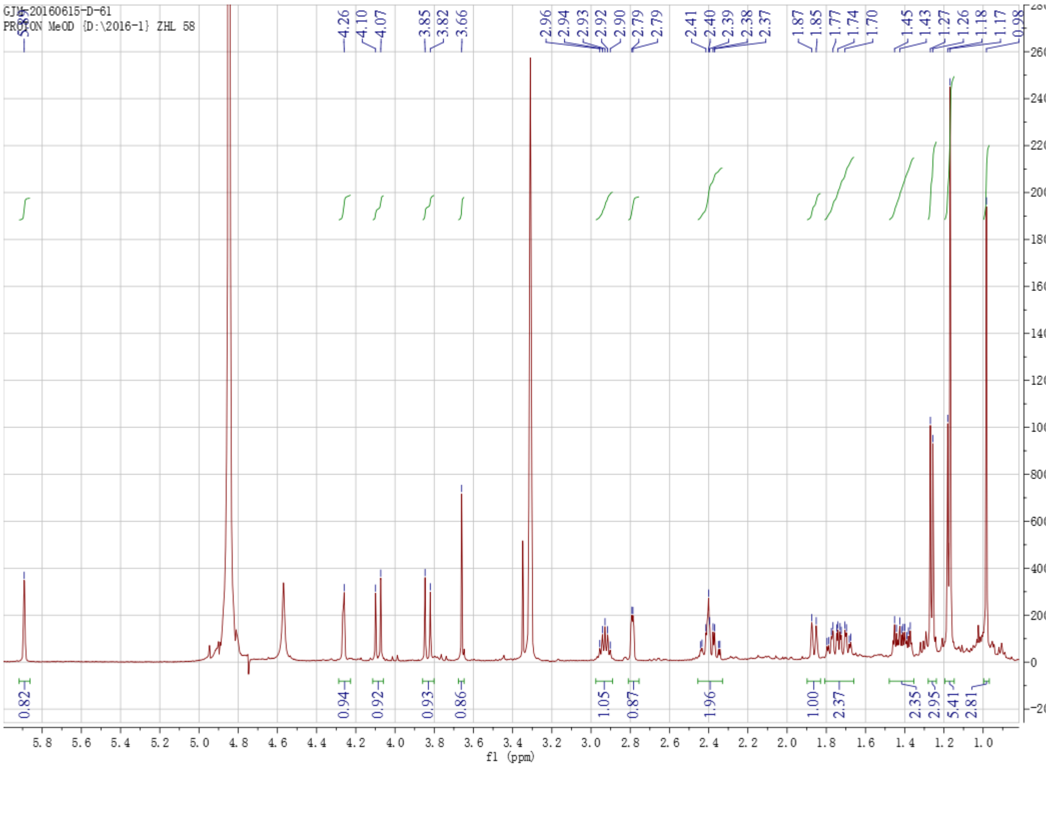

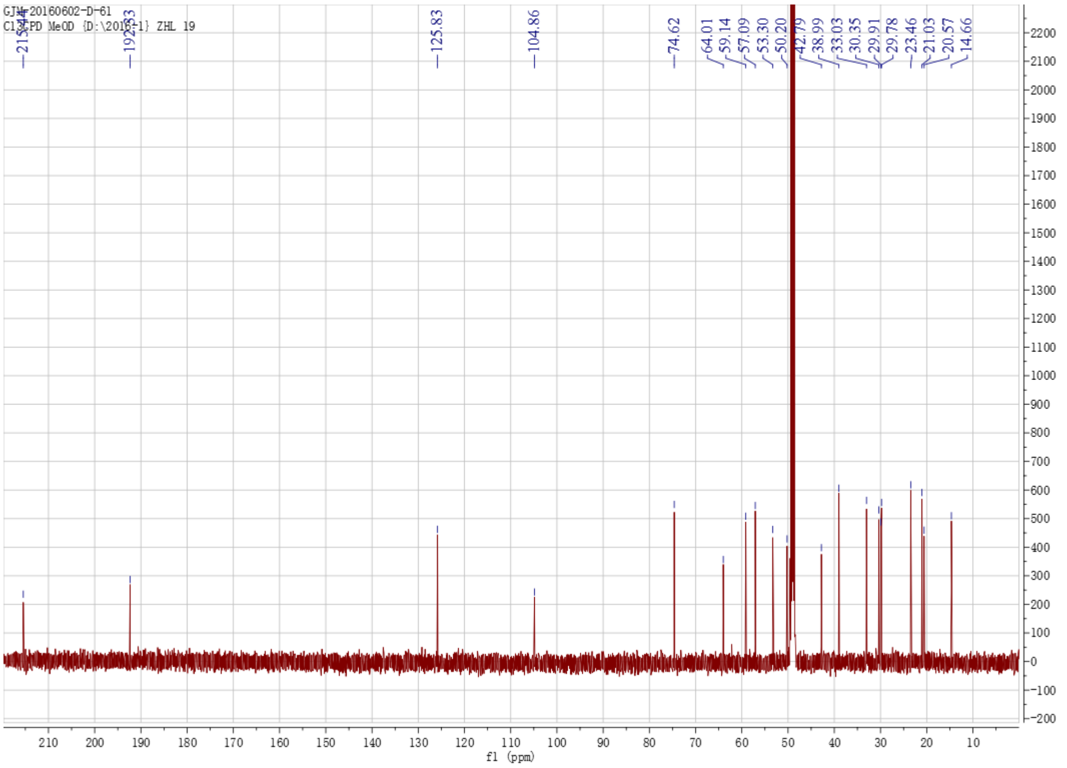

Figure S37. HSQC and ^{1}H-^{1}H COSY spectra of compound **8** in CD_3OD

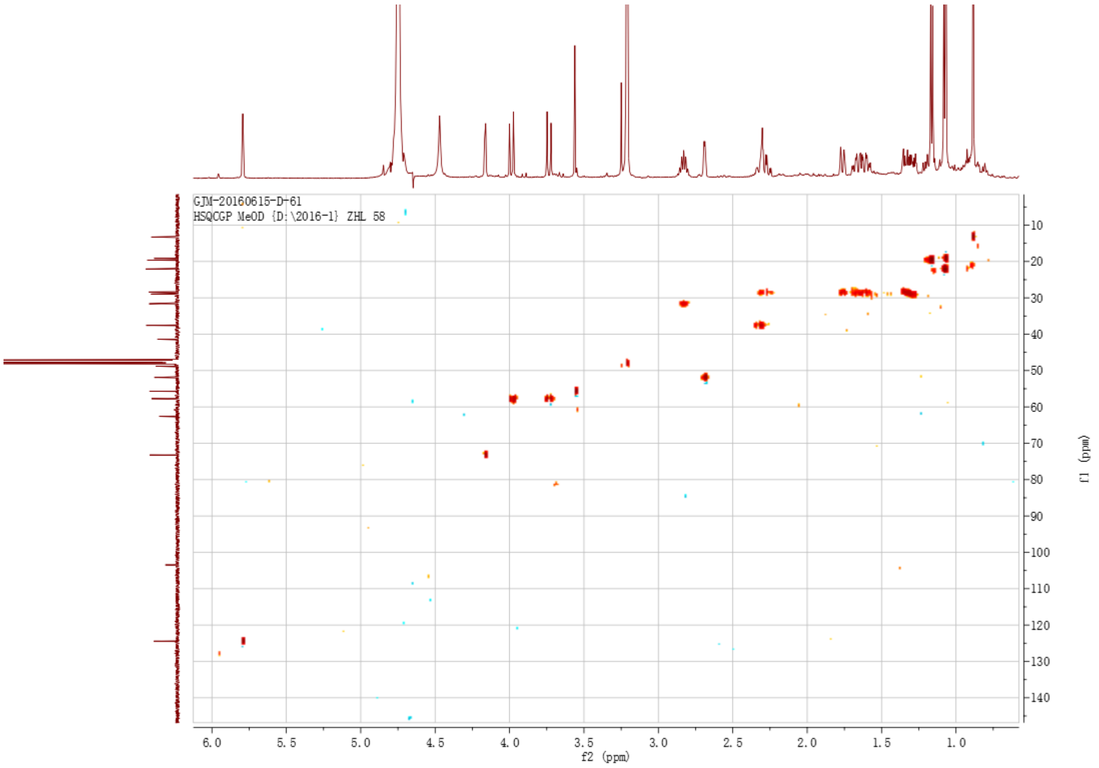

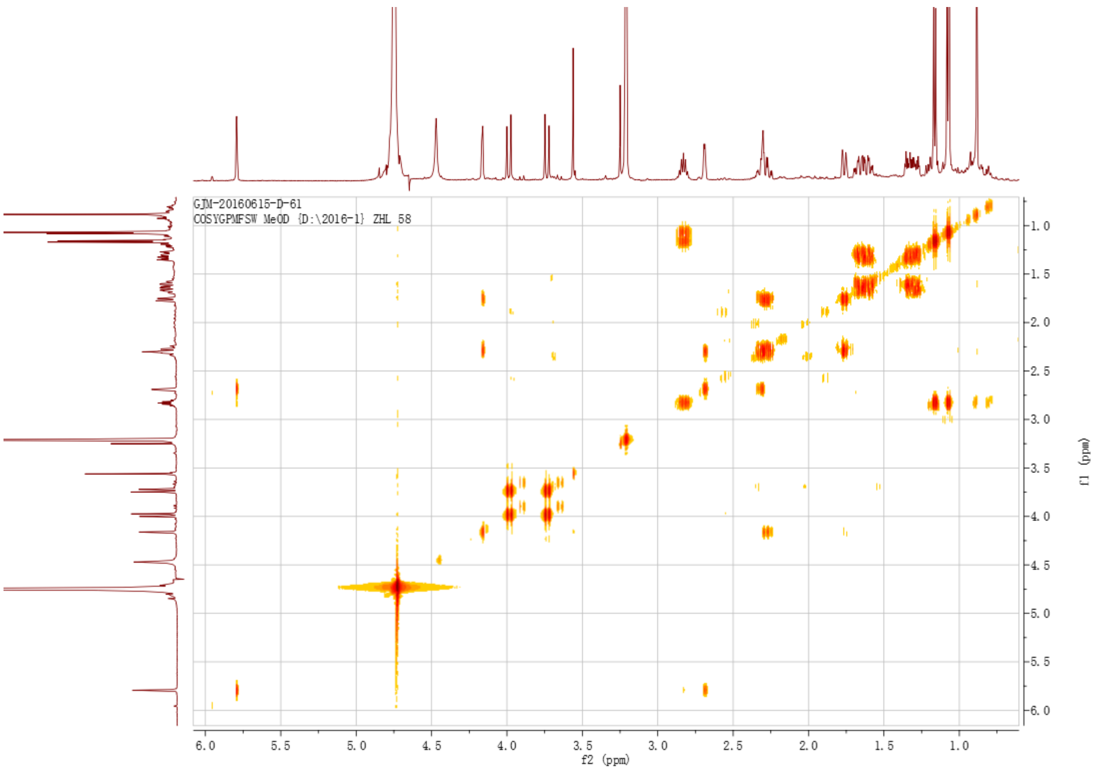

Figure S38. HMBC and NOESY spectra of compound **8** in CD_3OD.

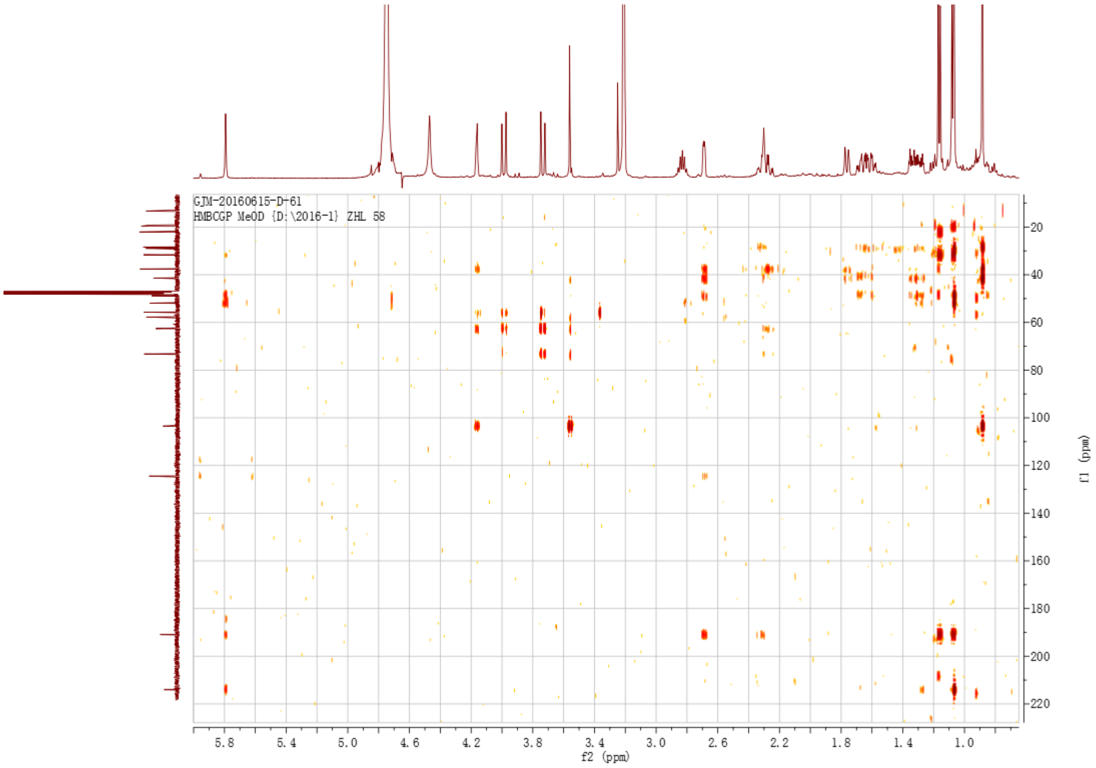

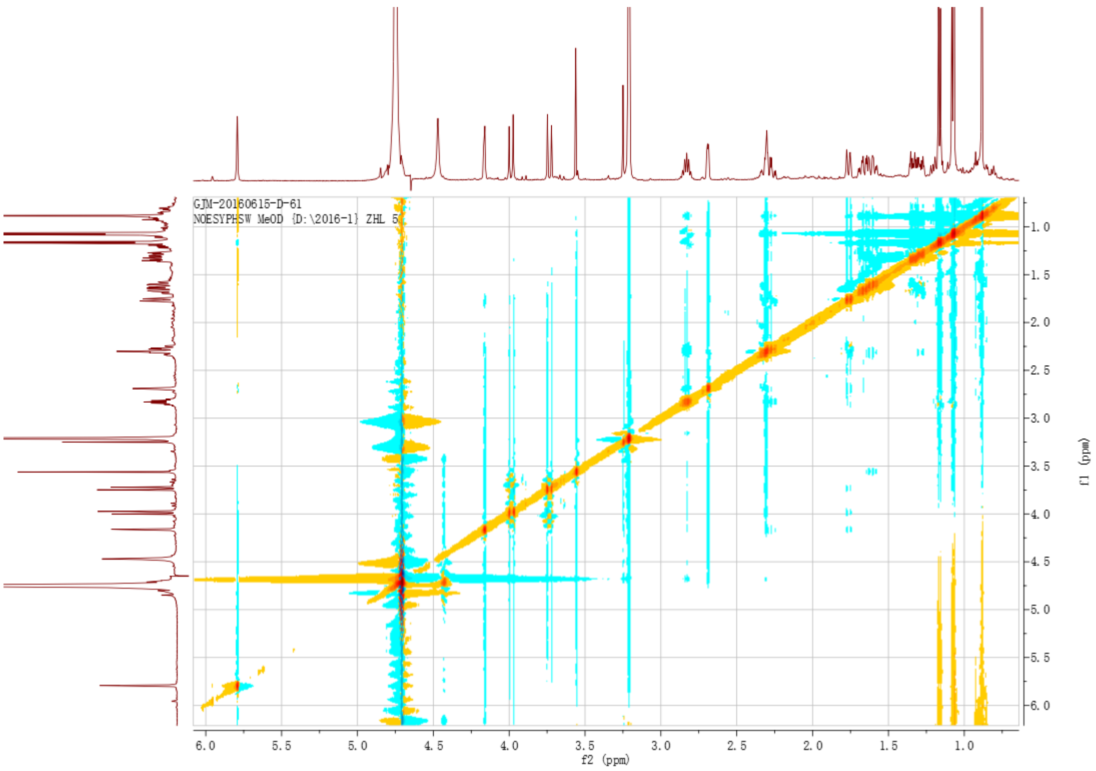

Figure S39. HRESIMS spectrum of compound **8**.

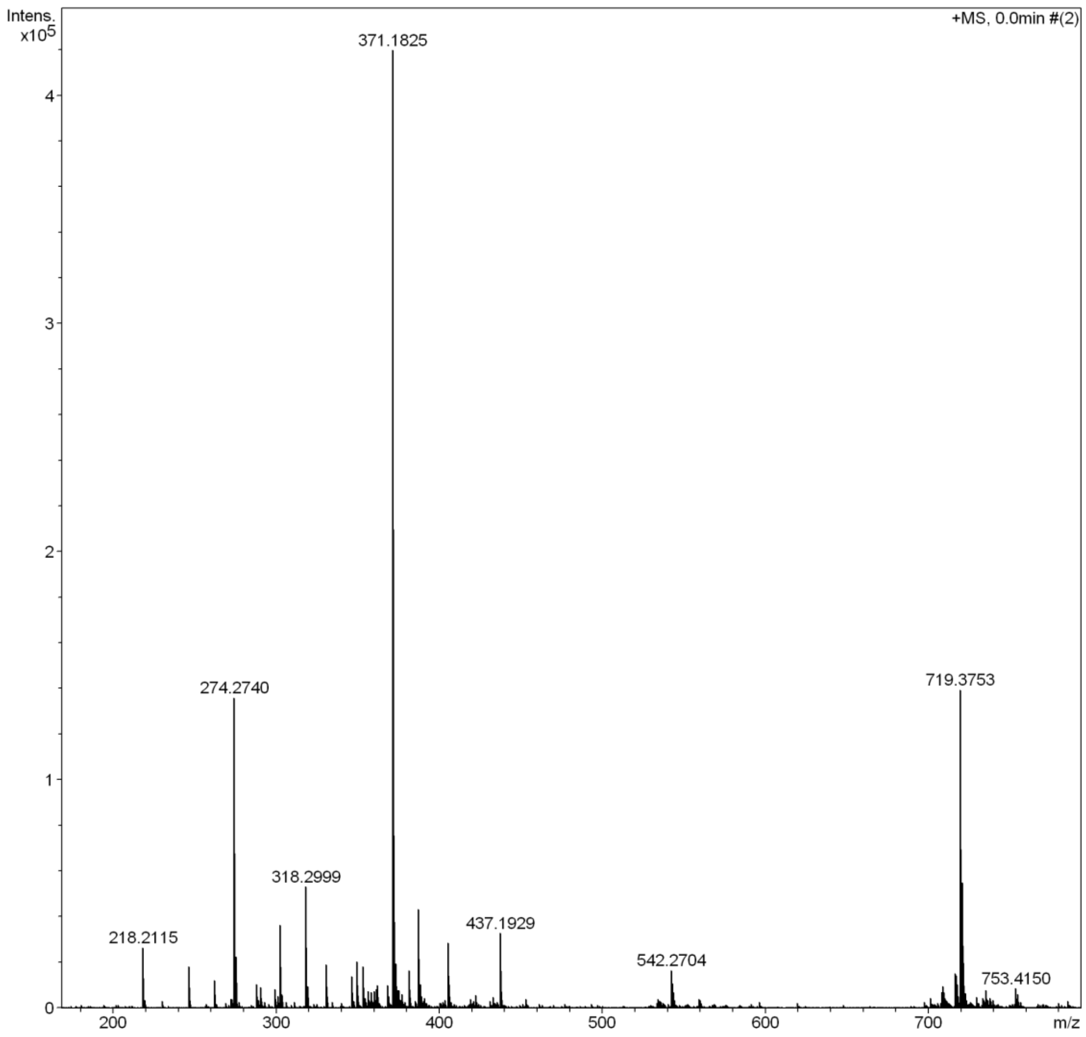

Figure S40. IR and UV spectra of compound **8**. This work is supported by Bruker (Beijing) Scientific Technology Co. Ltd.

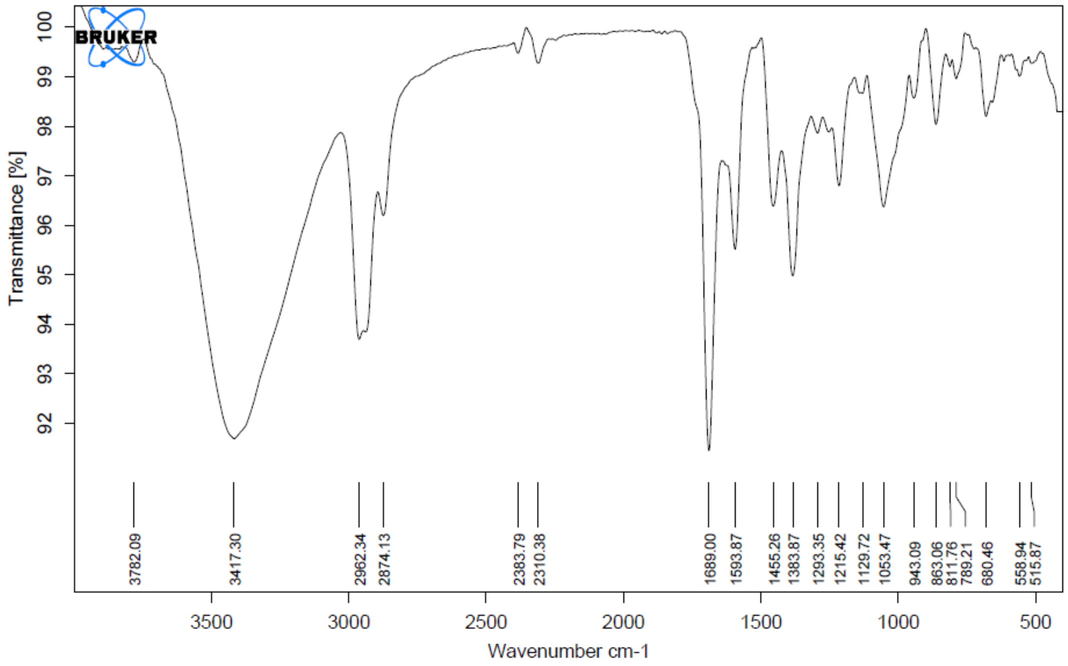

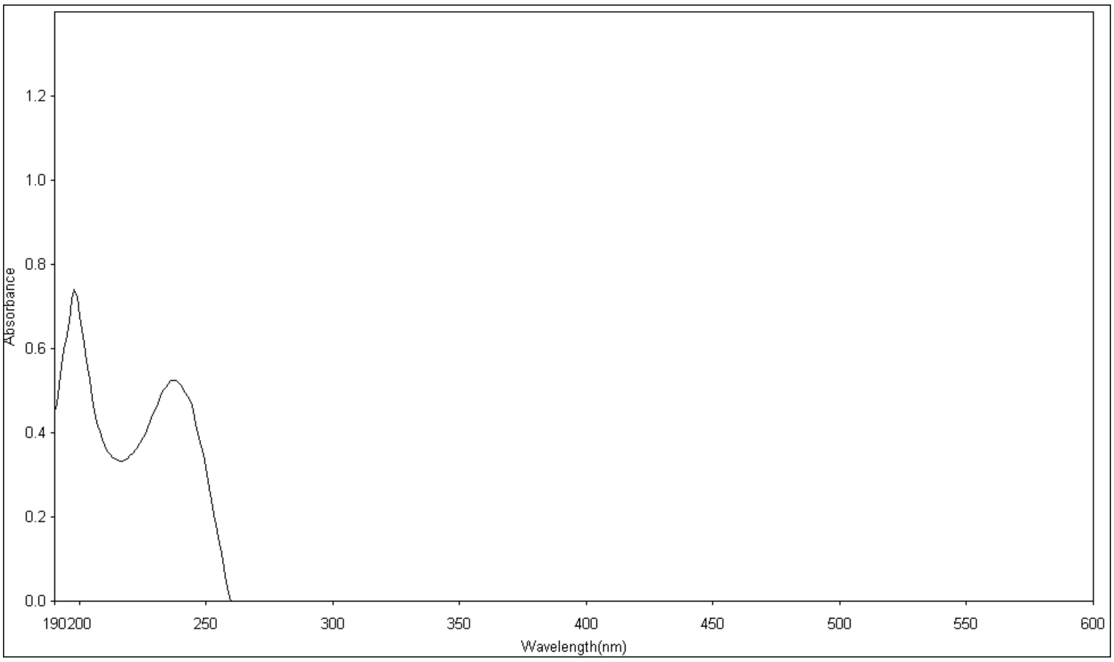

Figure S41. ^{1}H NMR and ^{13}C spectra of compound **9** in CD_3OD.

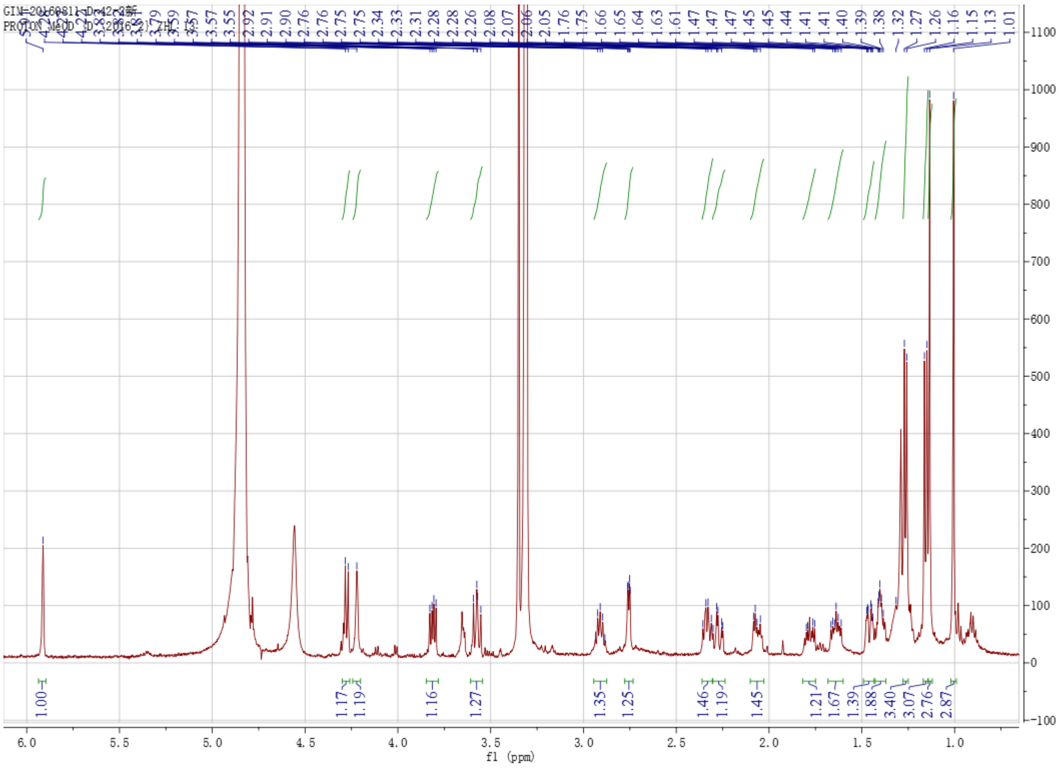

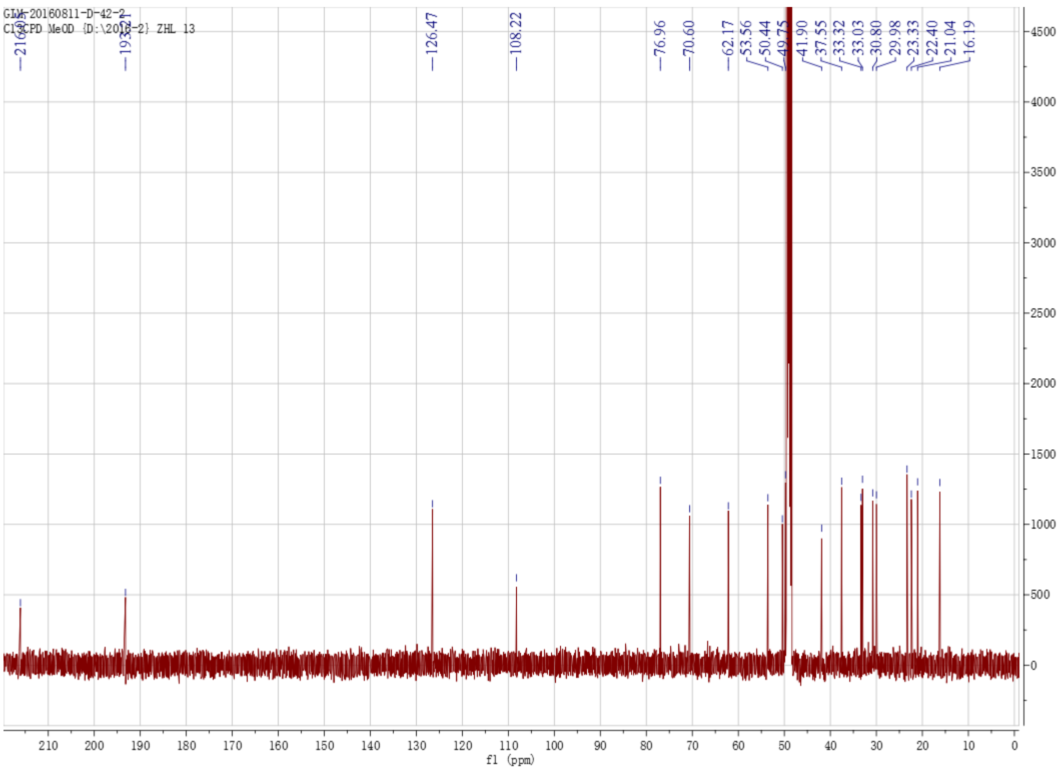

Figure S42. HSQC and ^{1}H-^{1}H COSY spectra of compound **9** in CD_3OD

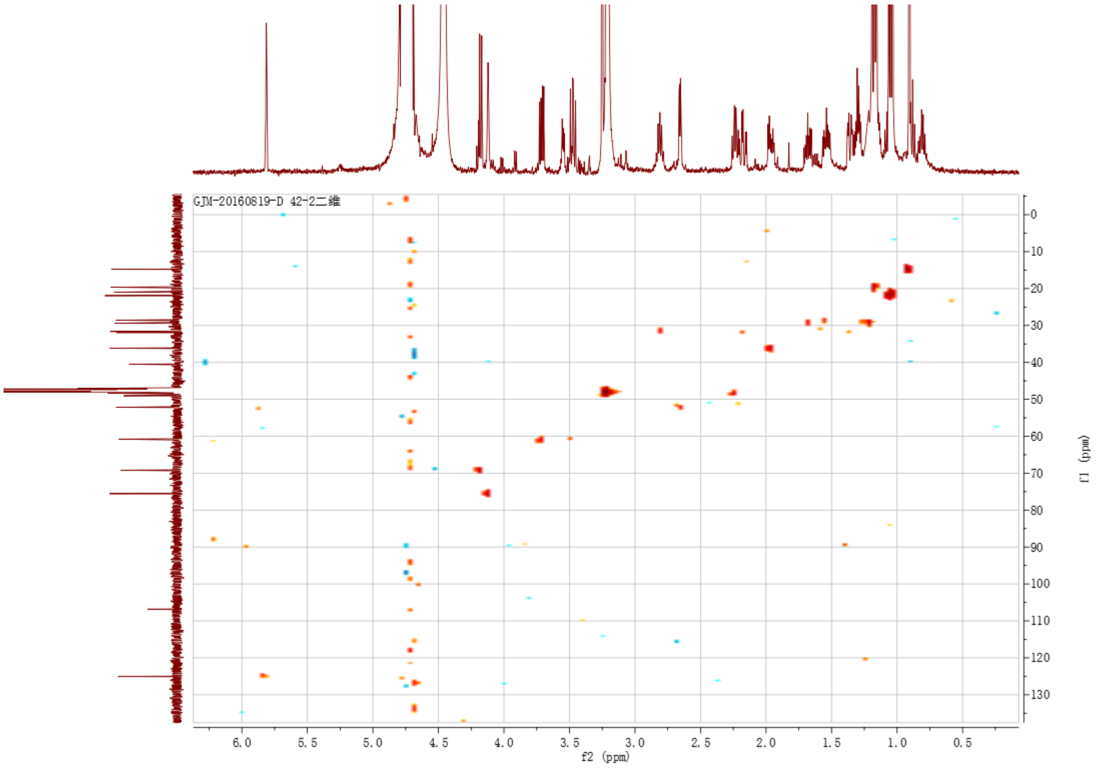

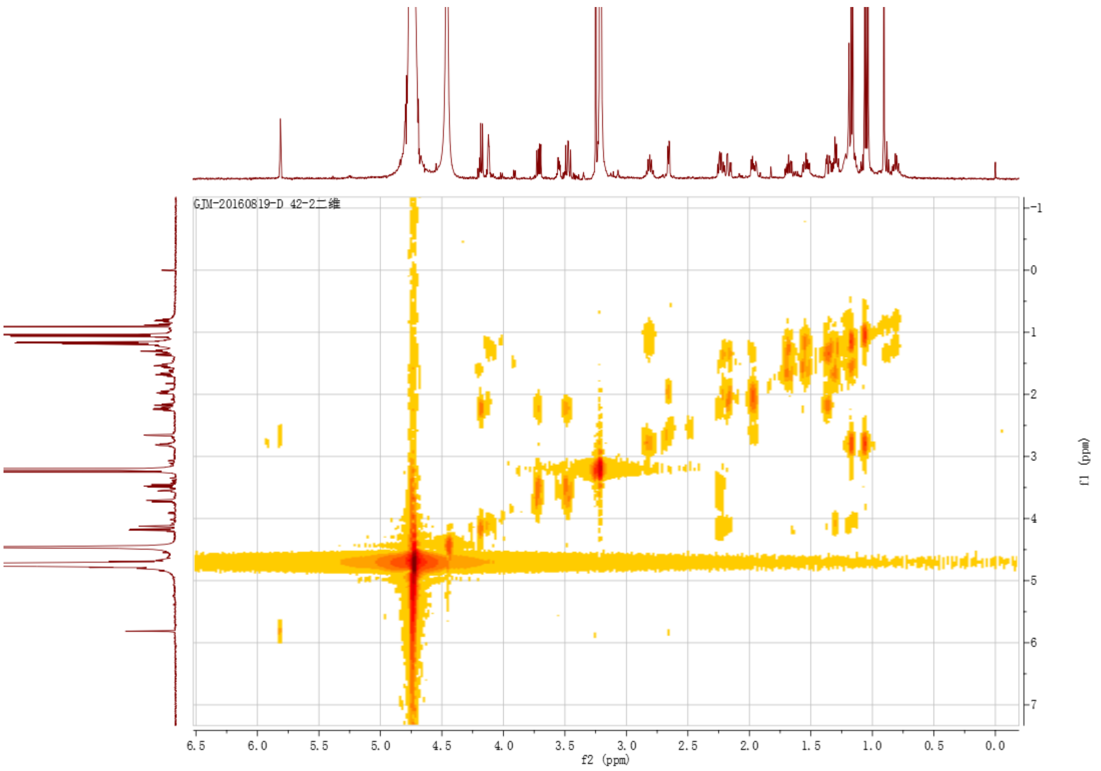

Figure S43. HMBC and NOESY spectra of compound **9** in CD_3OD.

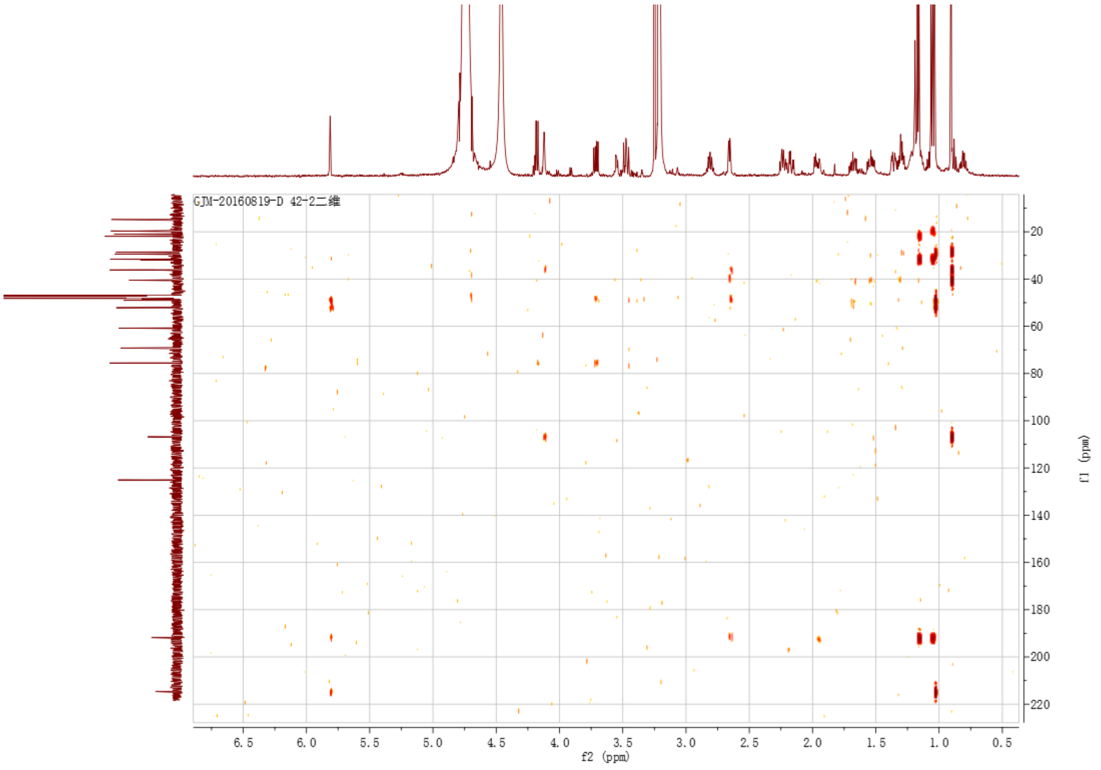

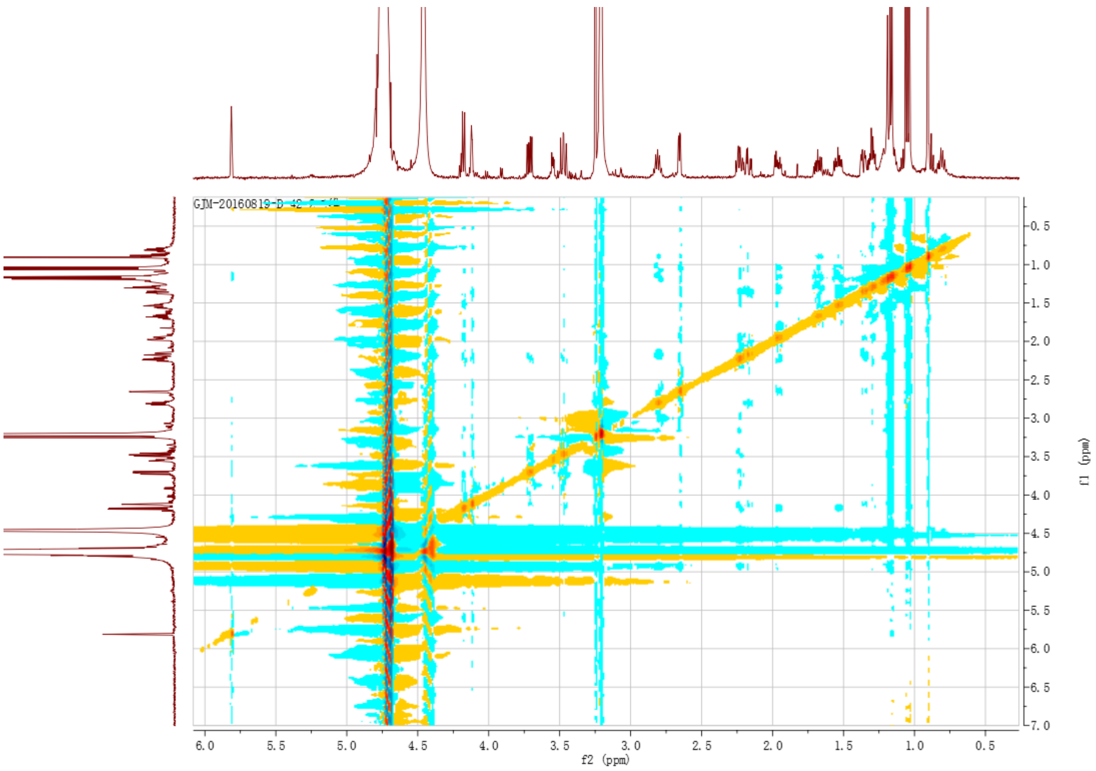

Figure S44. HRESIMS spectrum of compound **9**.

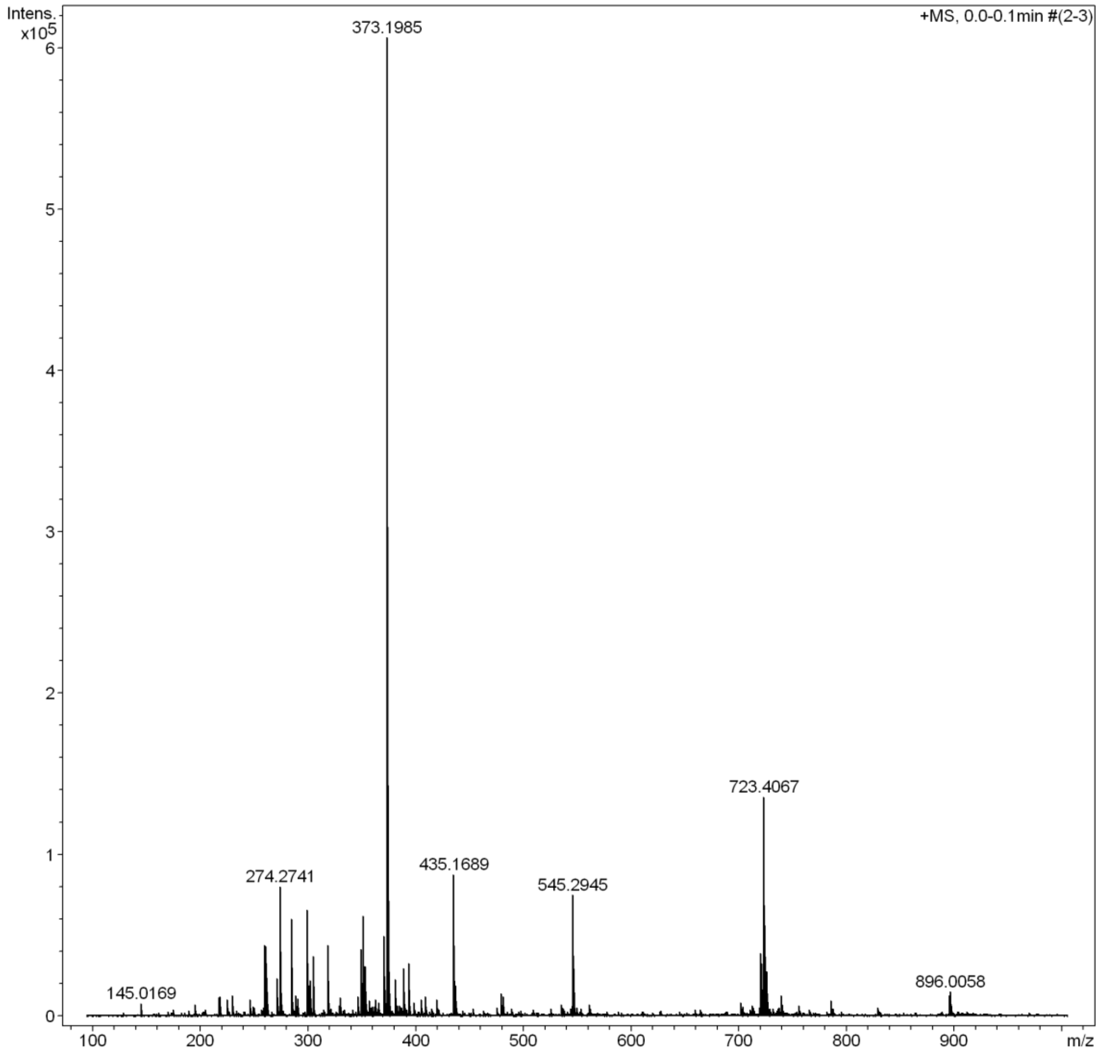

Figure S45. IR and UV spectra of compound **9**. This work is supported by Bruker (Beijing) Scientific Technology Co. Ltd.

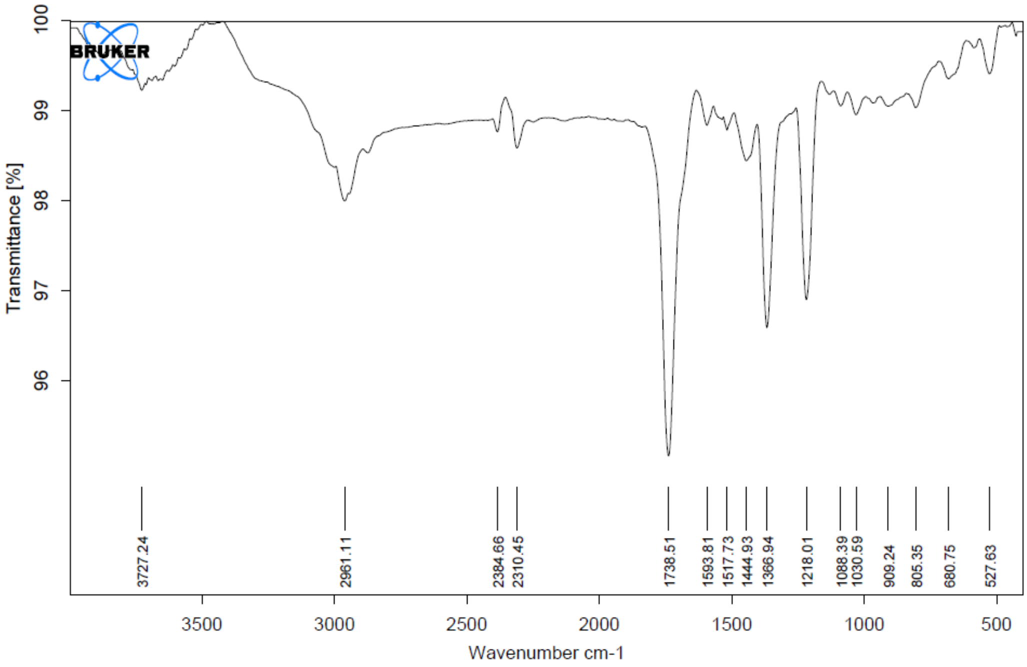

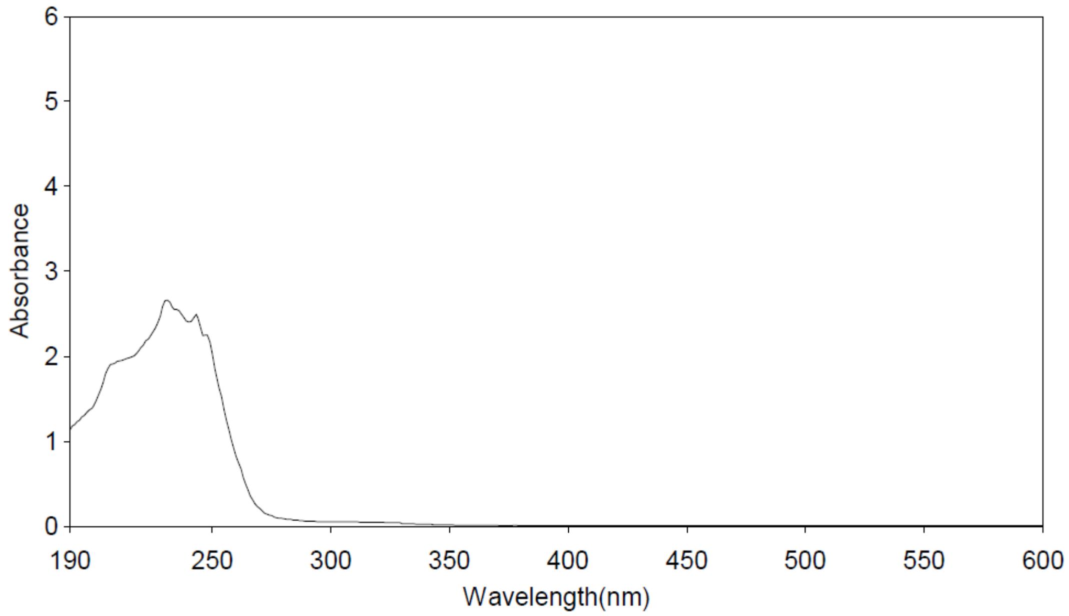

Figure S46. ^{1}H NMR and ^{13}C spectra of compound **10** in CD_3OD.

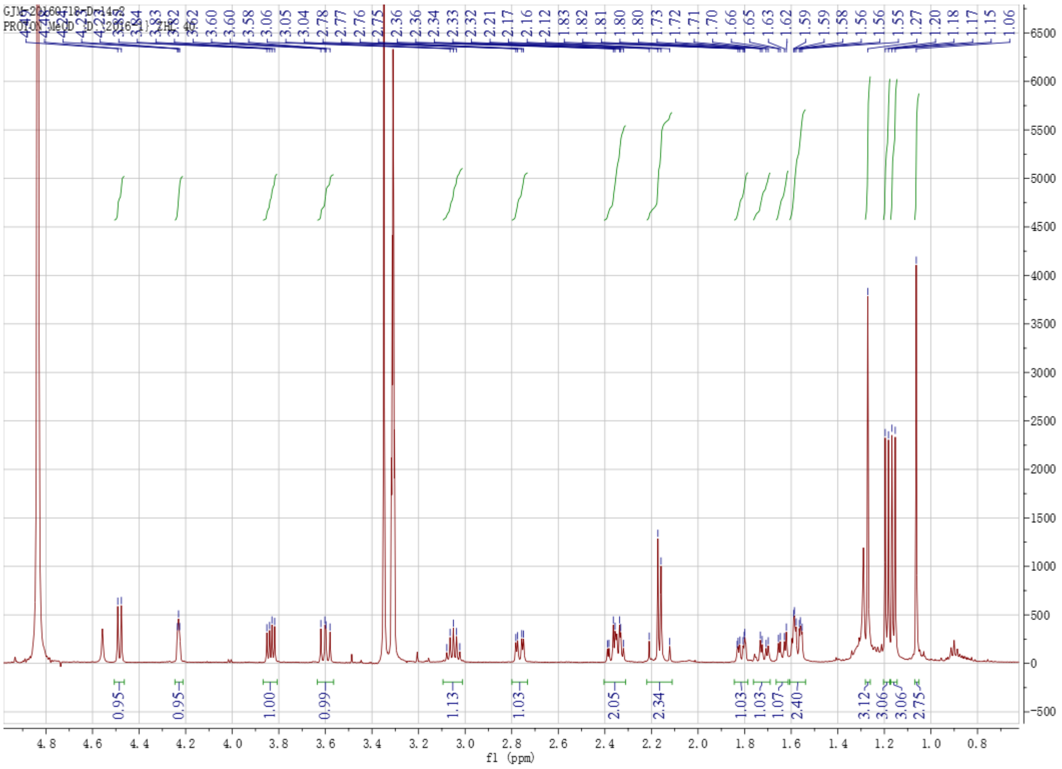

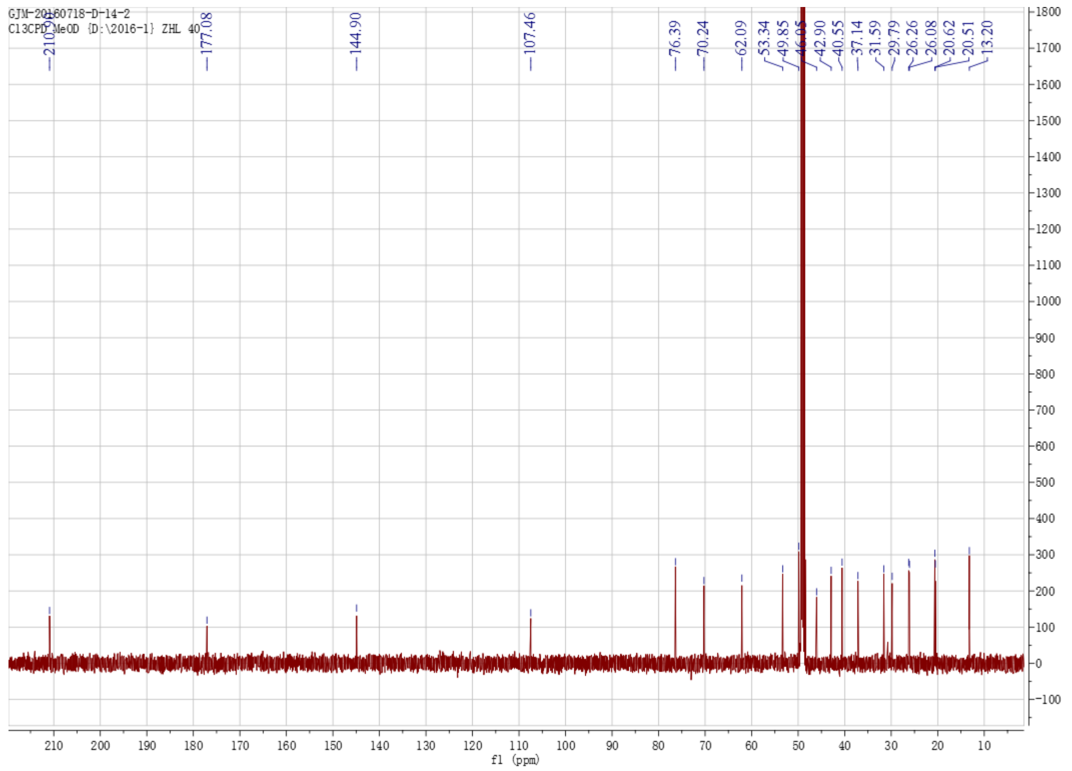

Figure S47. HSQC and ^{1}H-^{1}H COSY spectra of compound **10** in CD_3OD

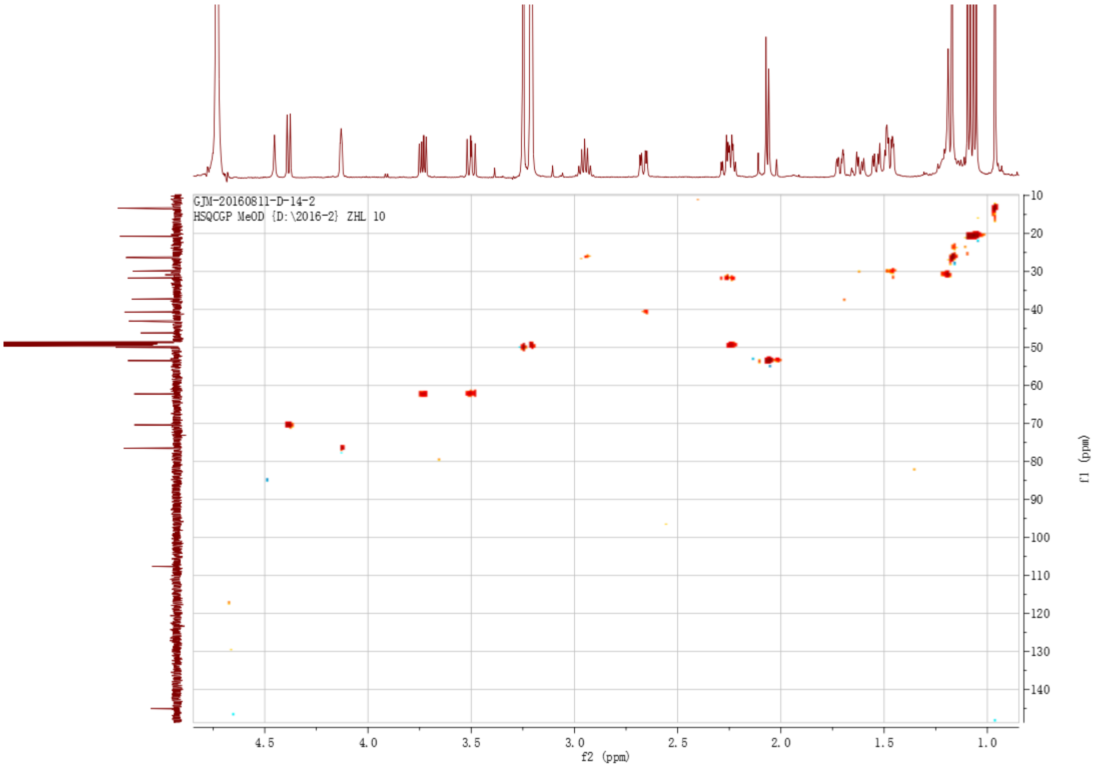

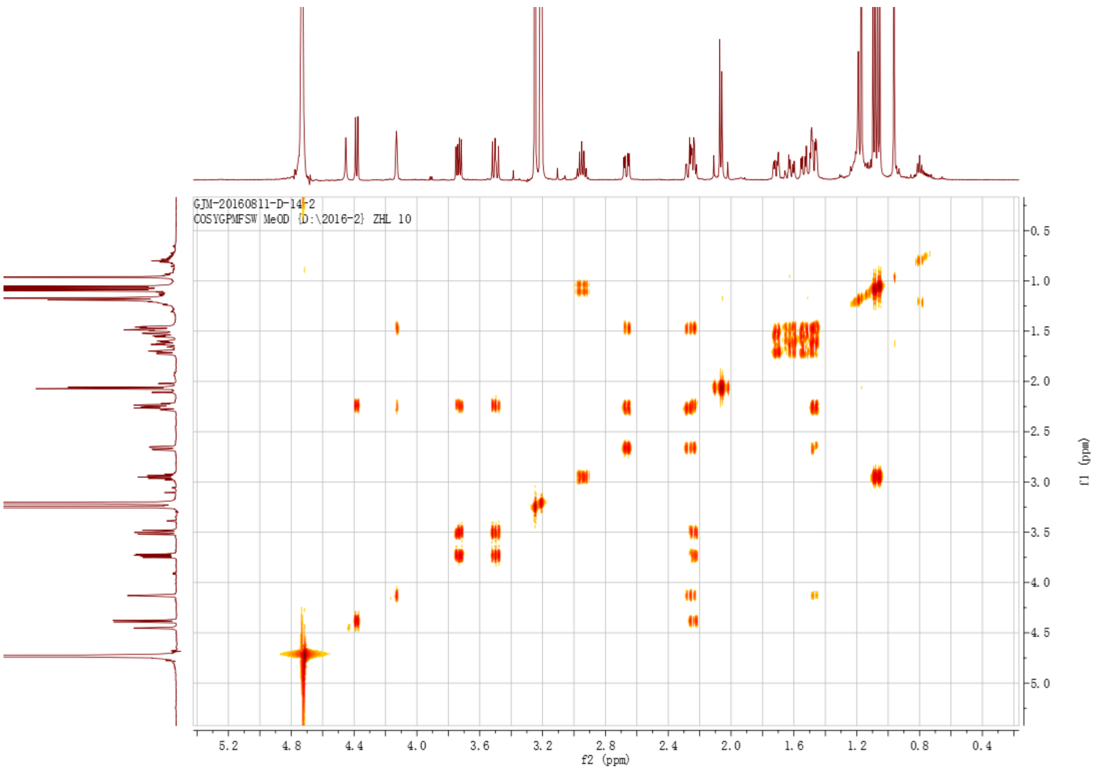

Figure S48. HMBC and NOESY spectra of compound **10** in CD_3OD.

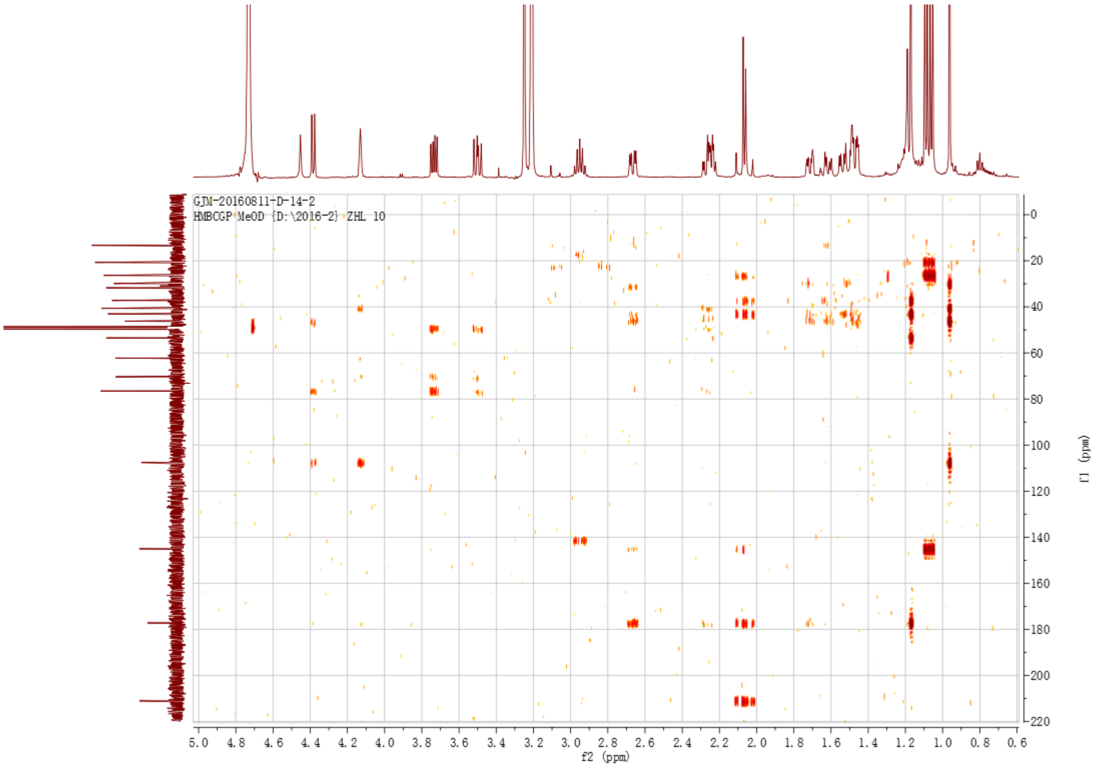

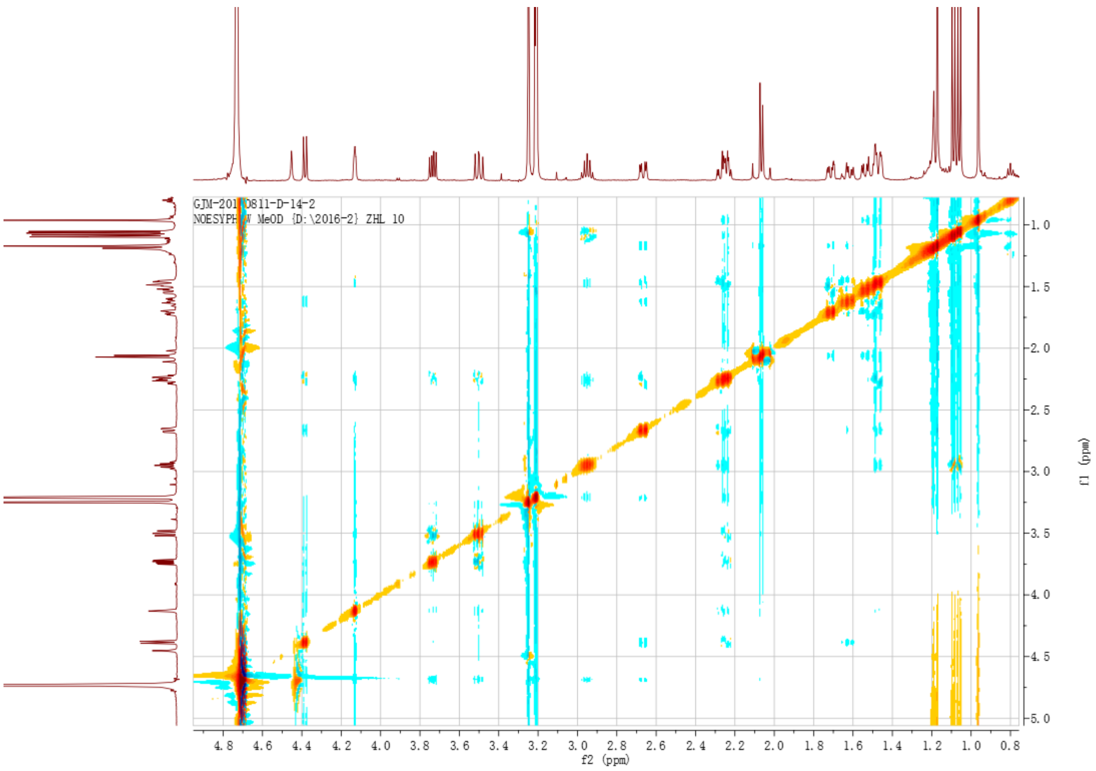

Figure S49. HRESIMS spectrum of compound **10**.

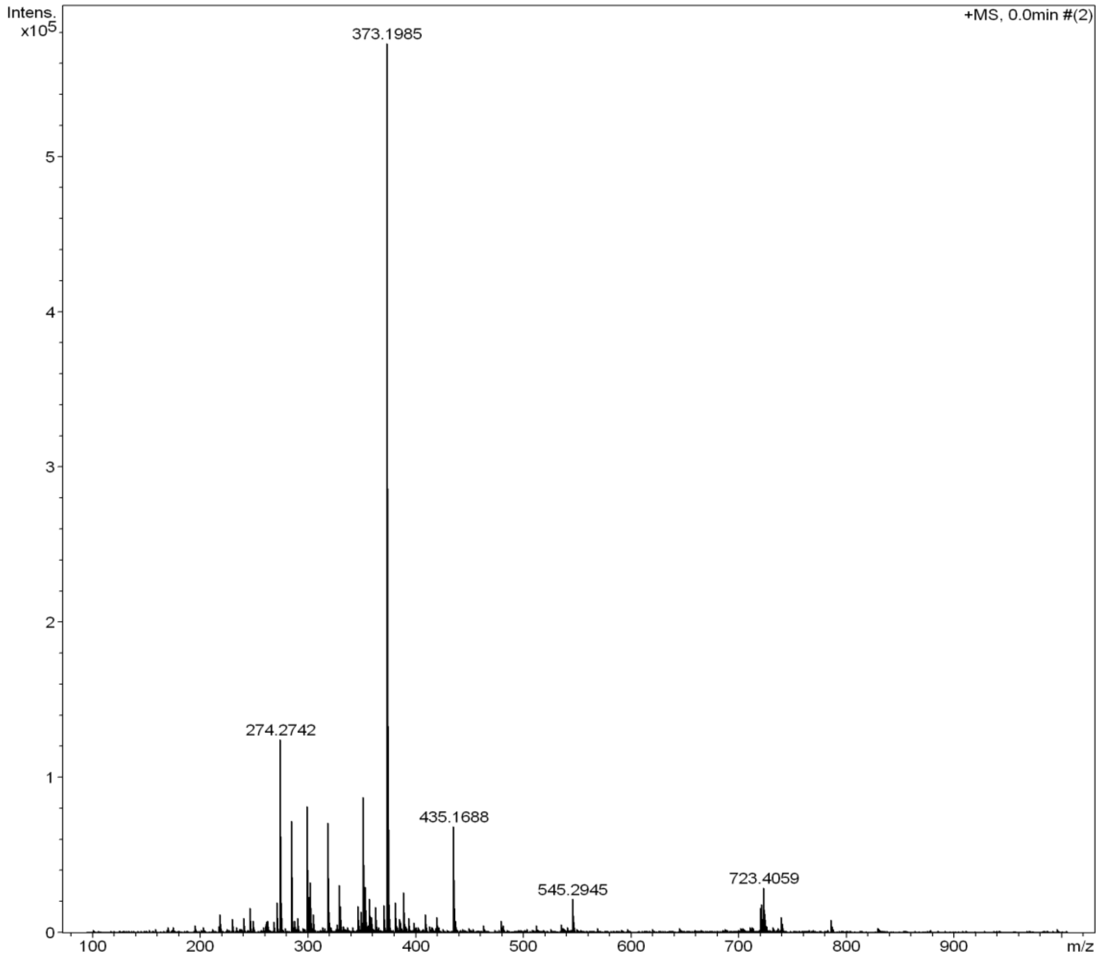

Figure S50. IR and UV spectra of compound **10**. This work is supported by Bruker (Beijing) Scientific Technology Co. Ltd.

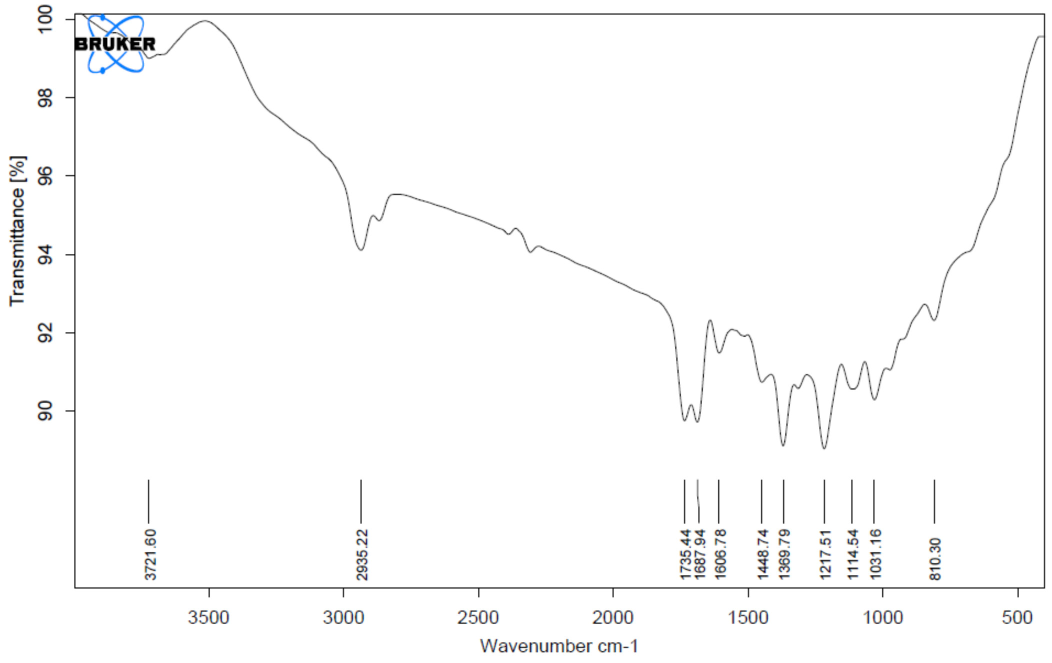

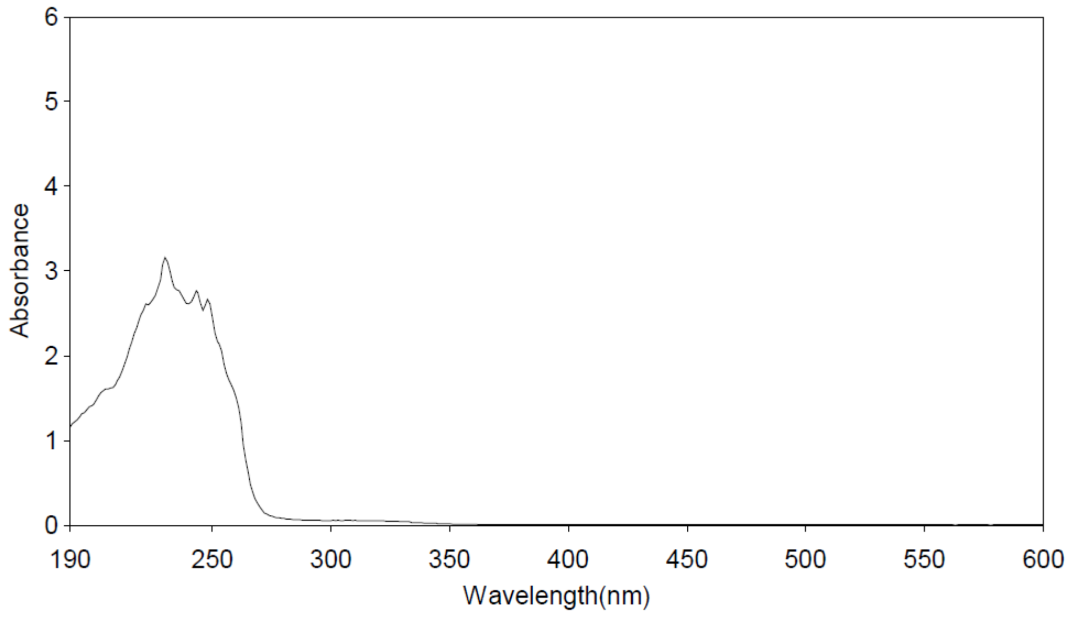

Figure S51. ^{1}H NMR and ^{13}C spectra of compound **11** in CD_3OD.

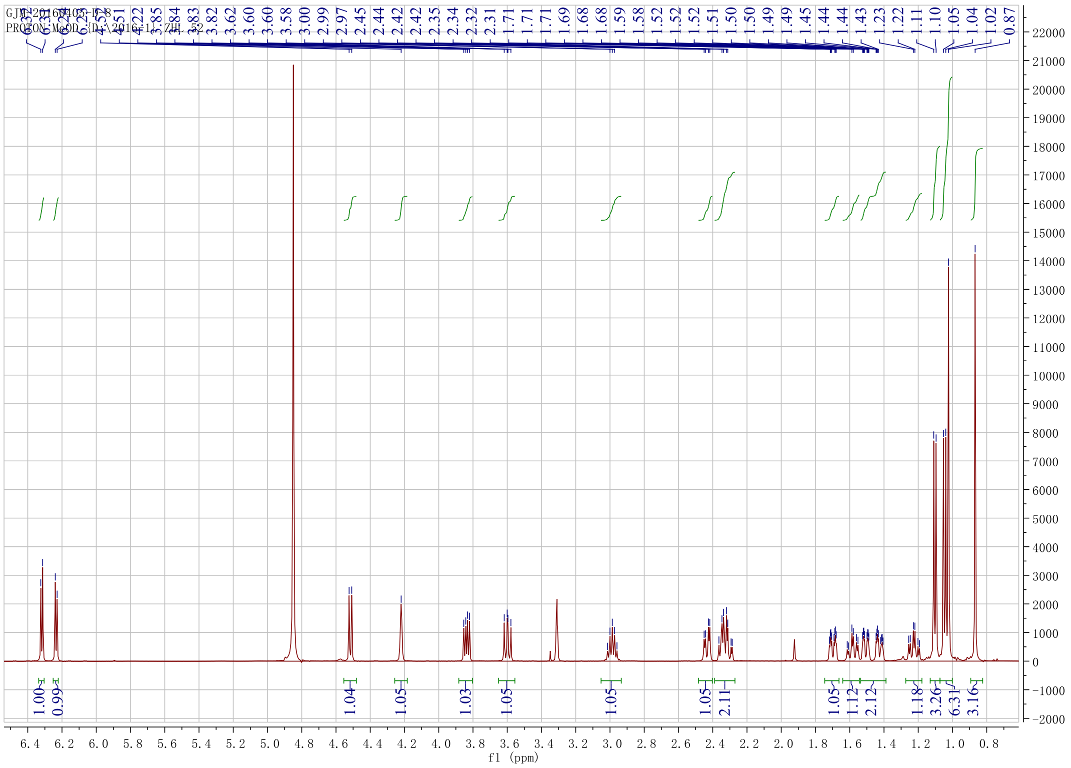

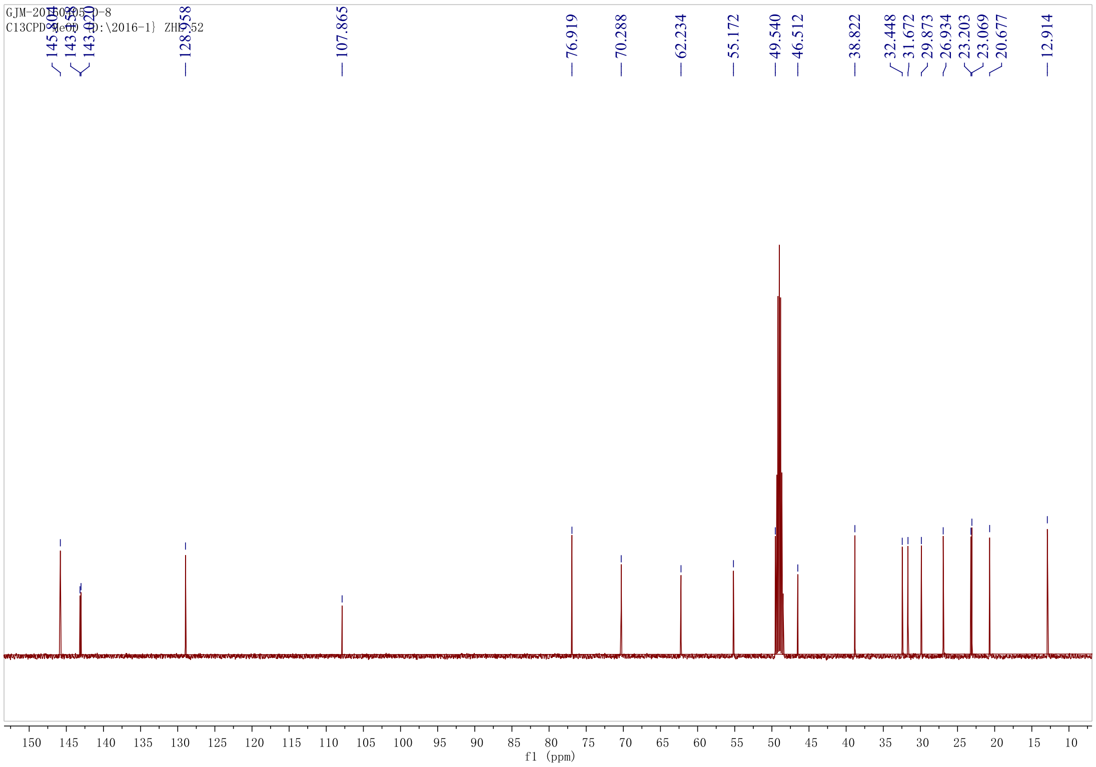

Figure S52. ESIMS spectrum of compound **11**.

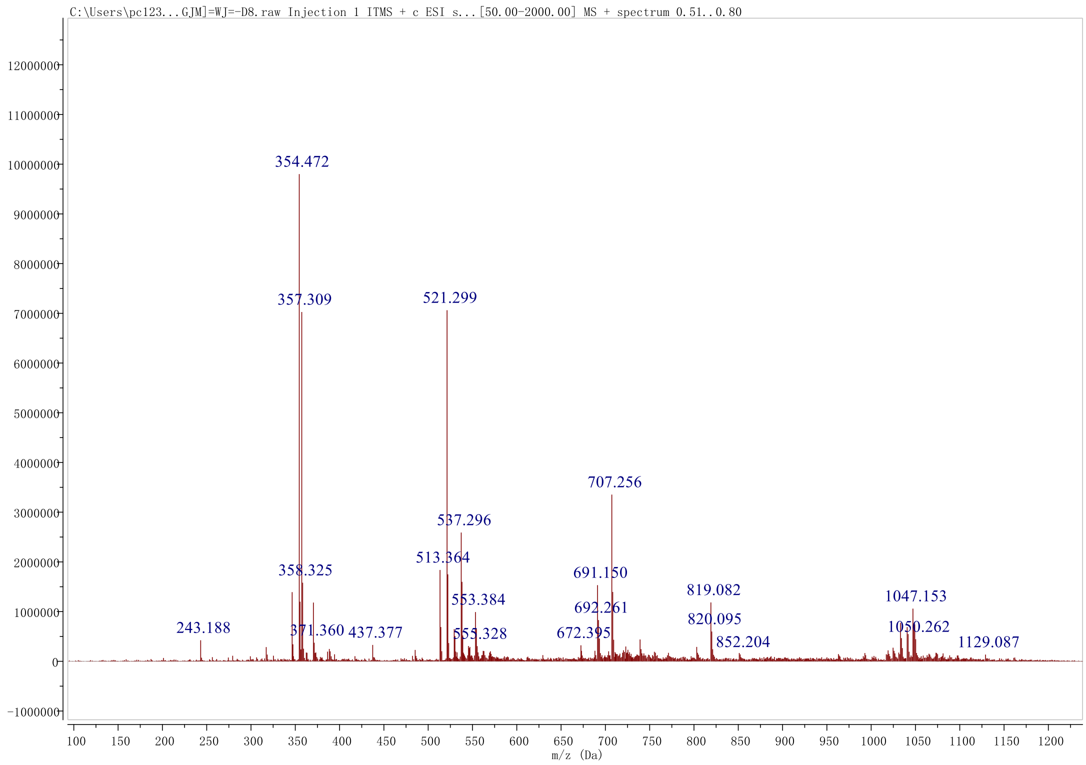

Figure S53. UV spectra of compound **11**.

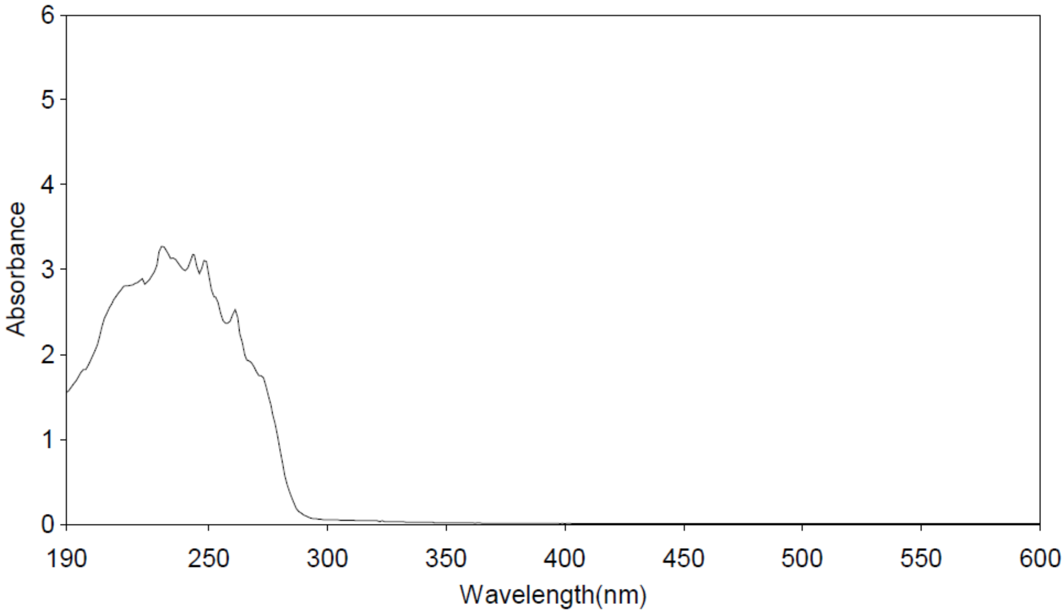

Figure S54. ^{1}H NMR and ^{13}C spectra of compound **12** in C_5D_5N.

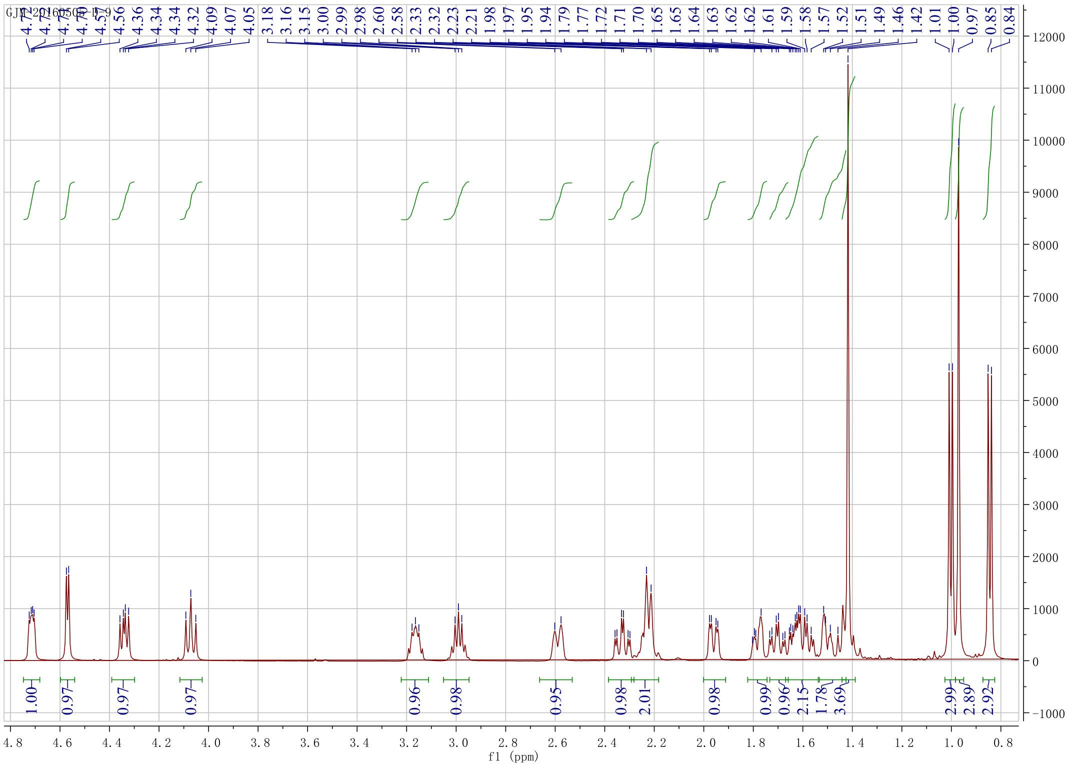

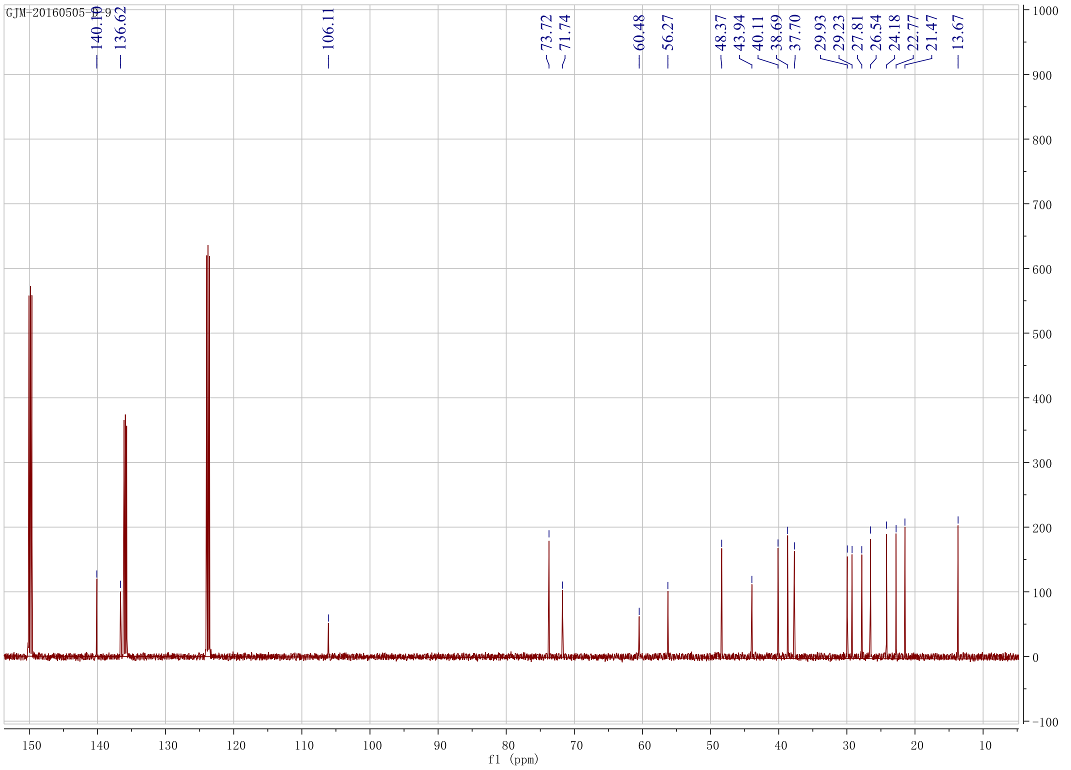

Figure S55. ESIMS spectrum of compound **12**.

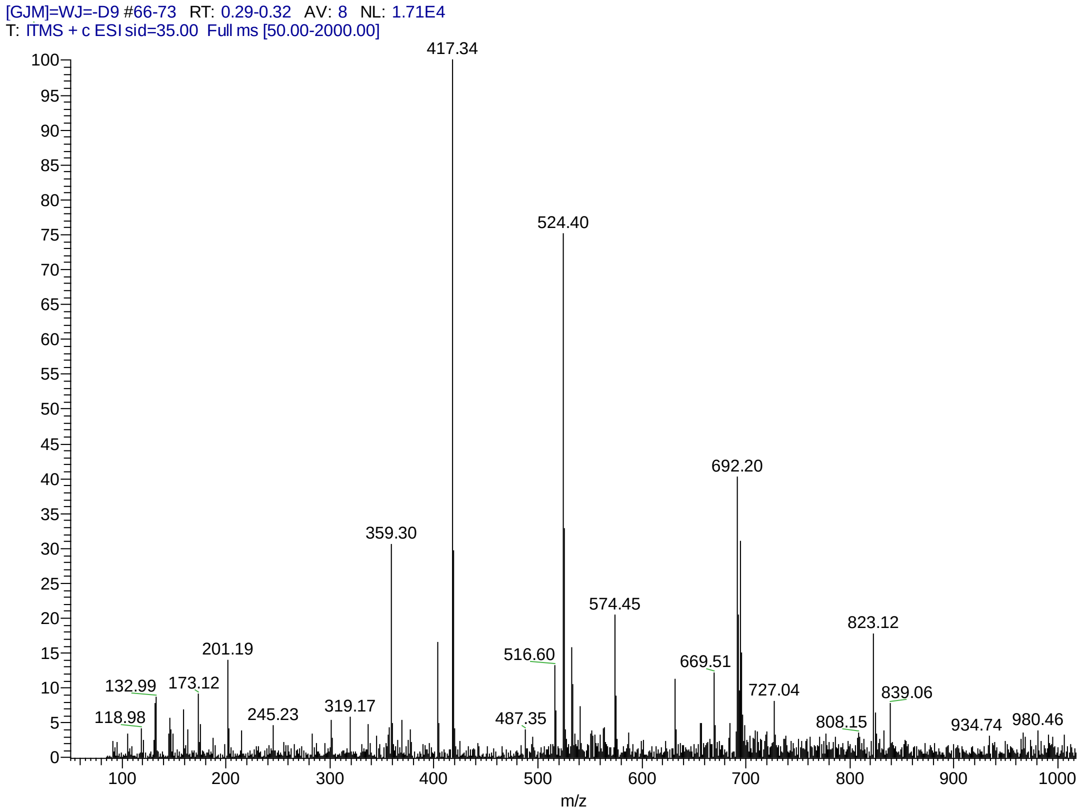

Figure S56. UV spectra of compound **12**.

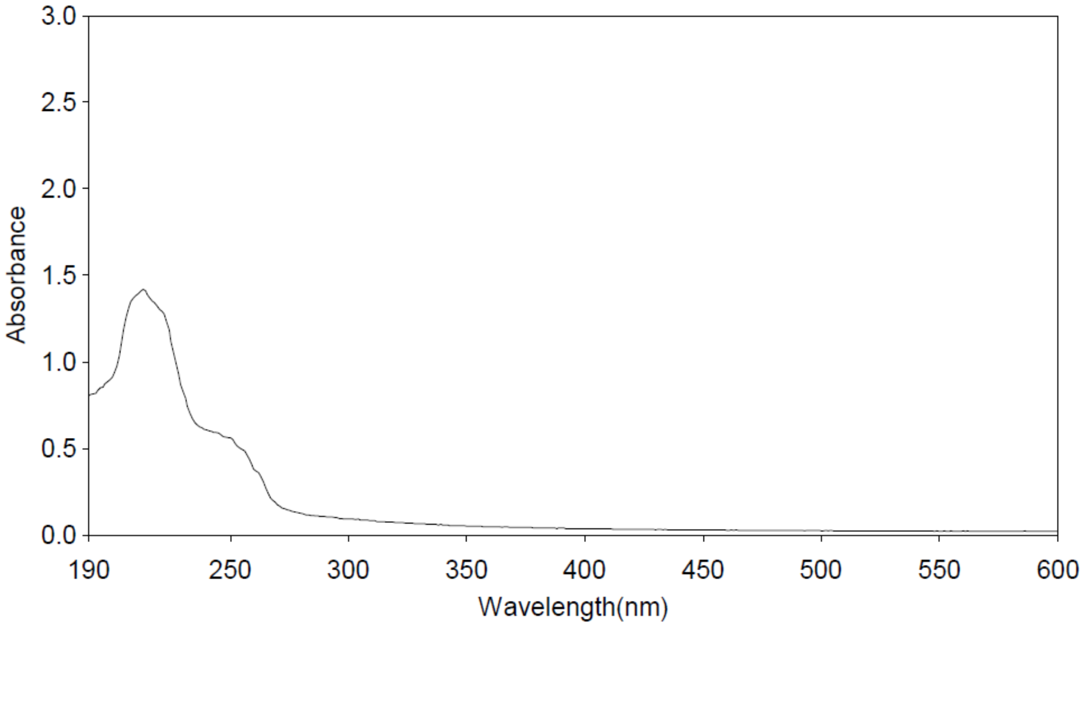

Figure S57. ^{1}H NMR and ^{13}C spectra of compound **13** in CD_3OD.

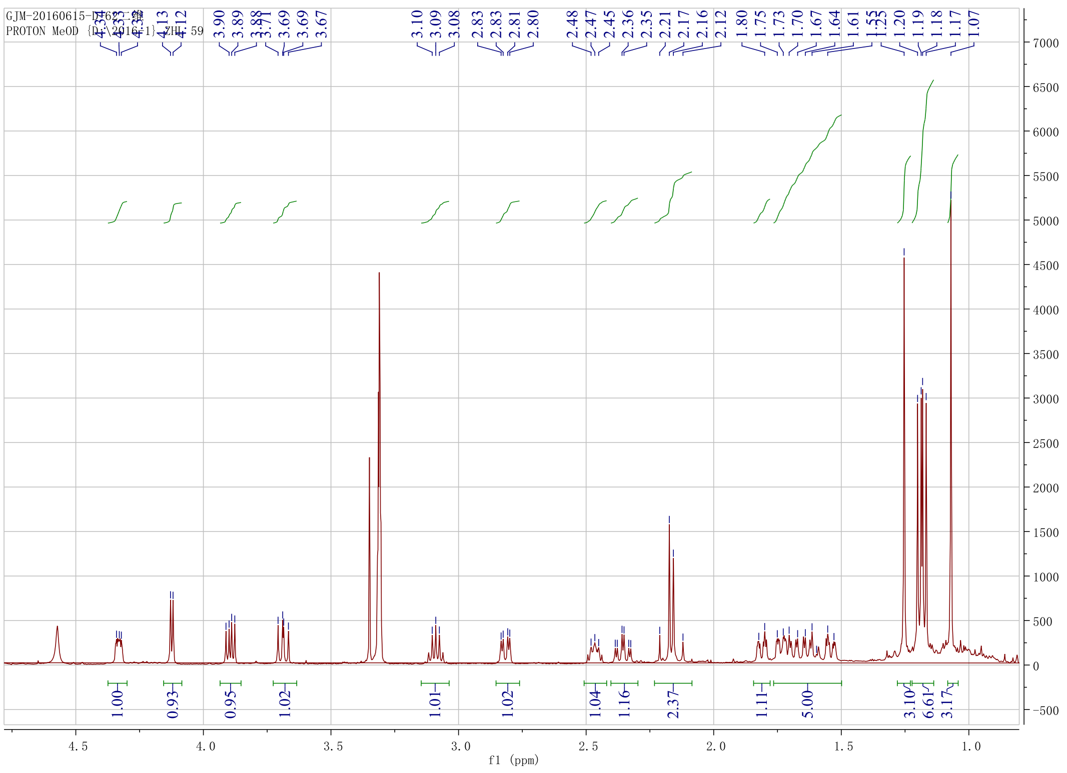

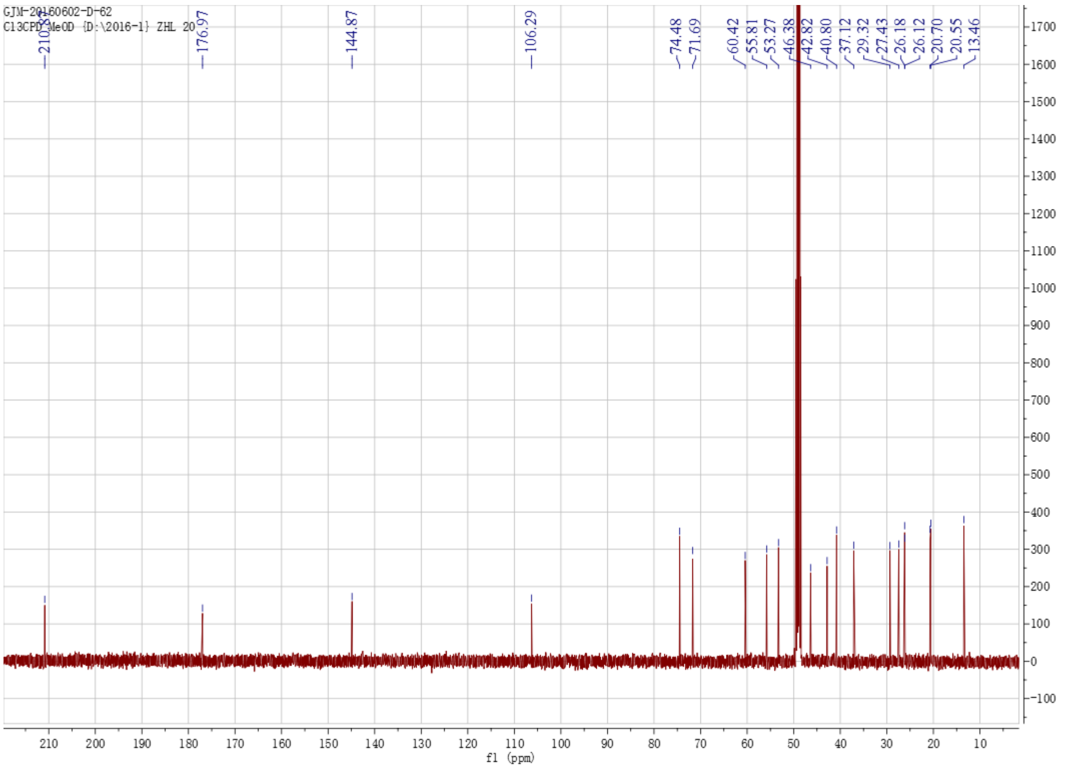

Figure S58. ESIMS spectrum of compound **13**.

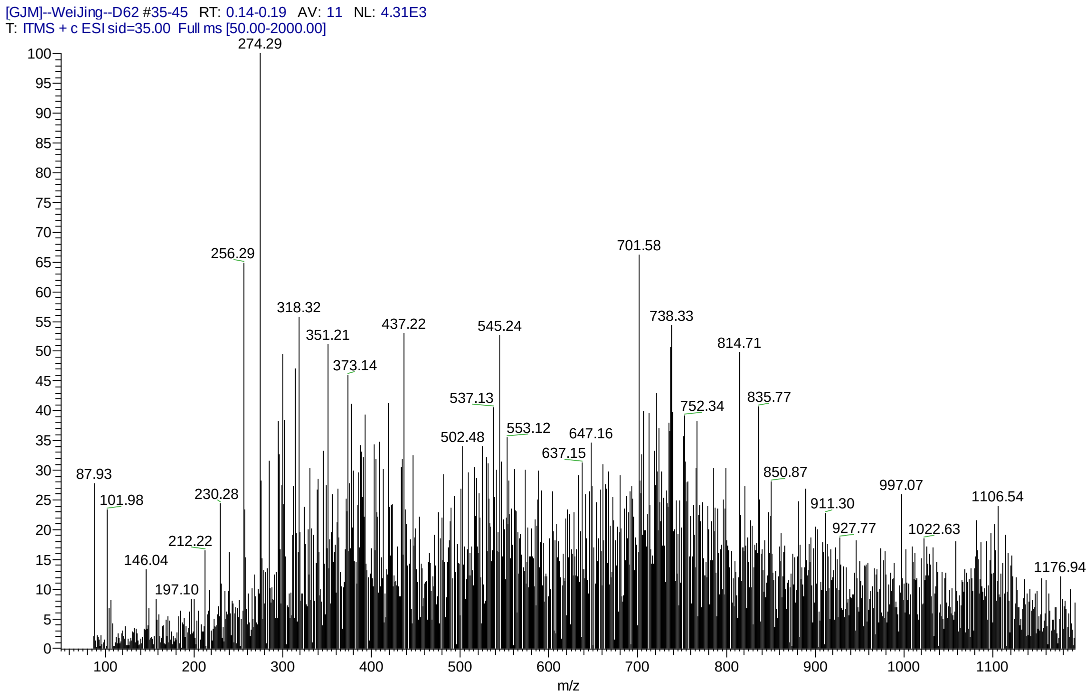

Figure S59. UV spectra of compound **13**.

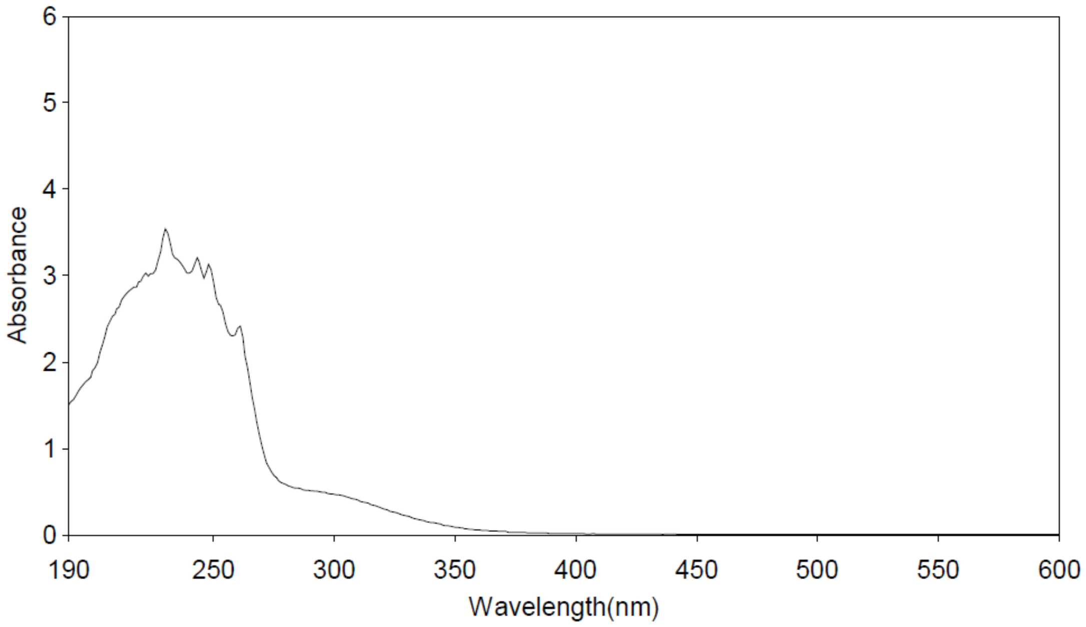

Figure S60. ^{1}H NMR and ^{13}C spectra of compound **14** in CD_3OD.

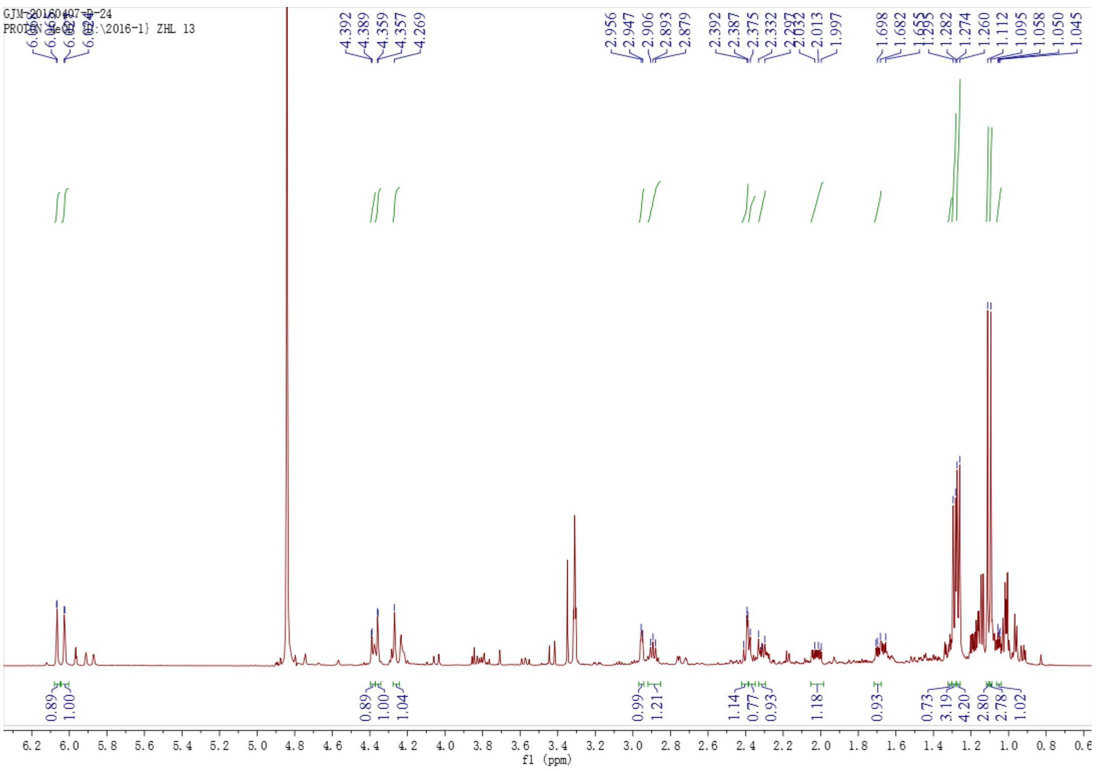

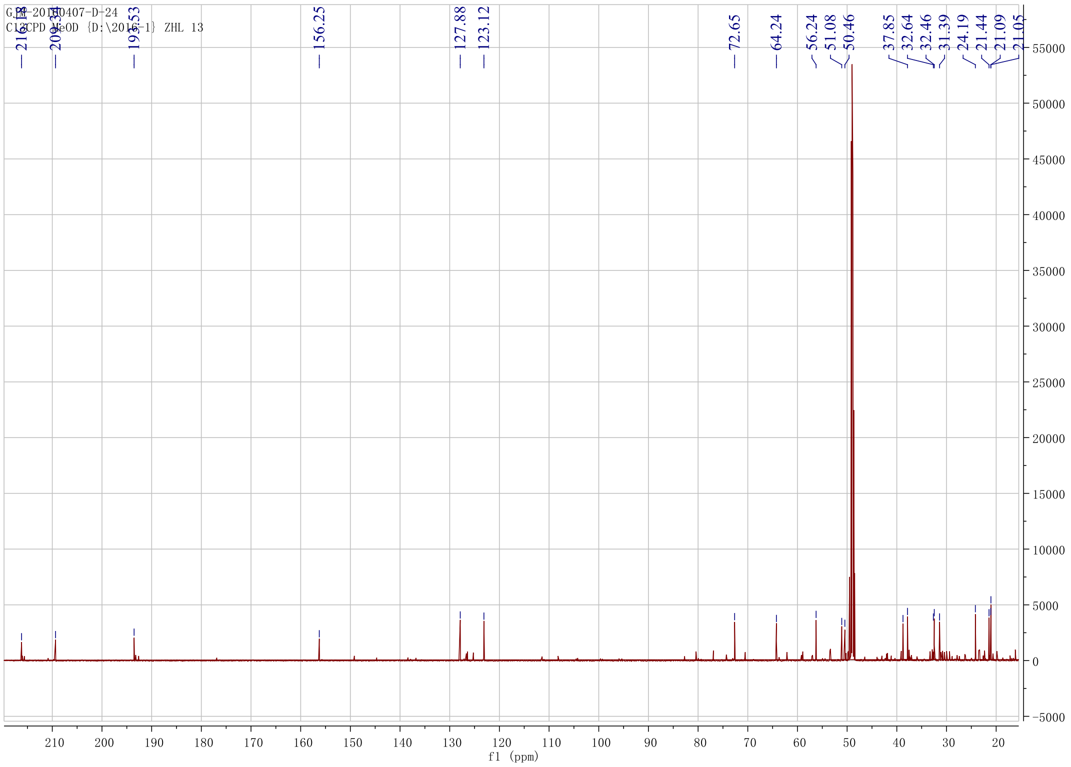

Figure S61. ESIMS spectrum of compound **14**.

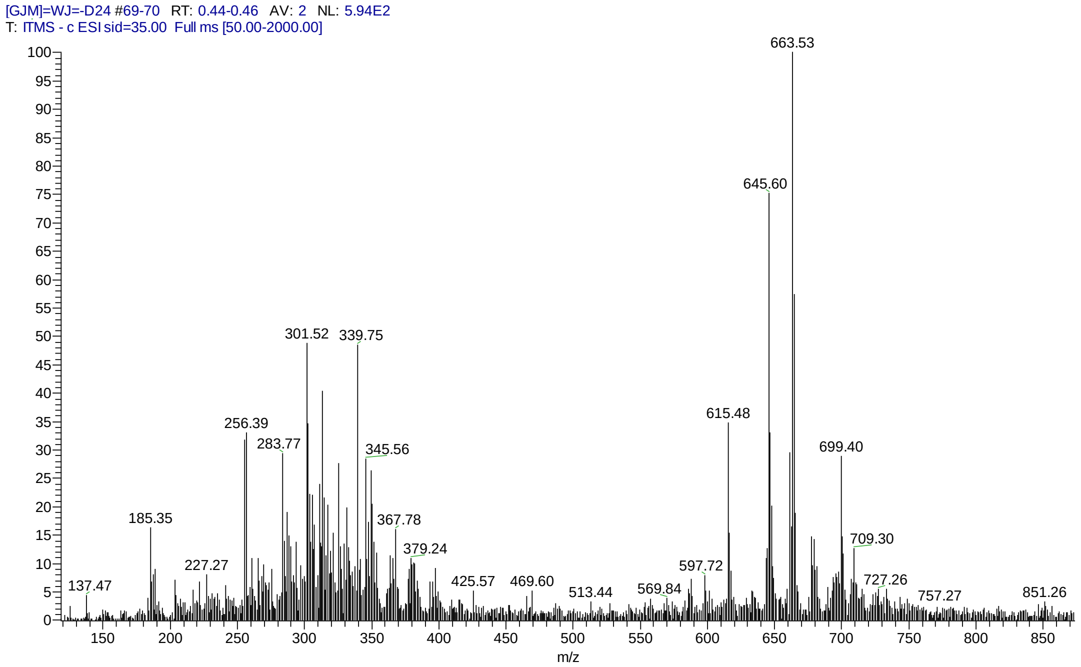

Figure S62. UV spectra of compound **14**.

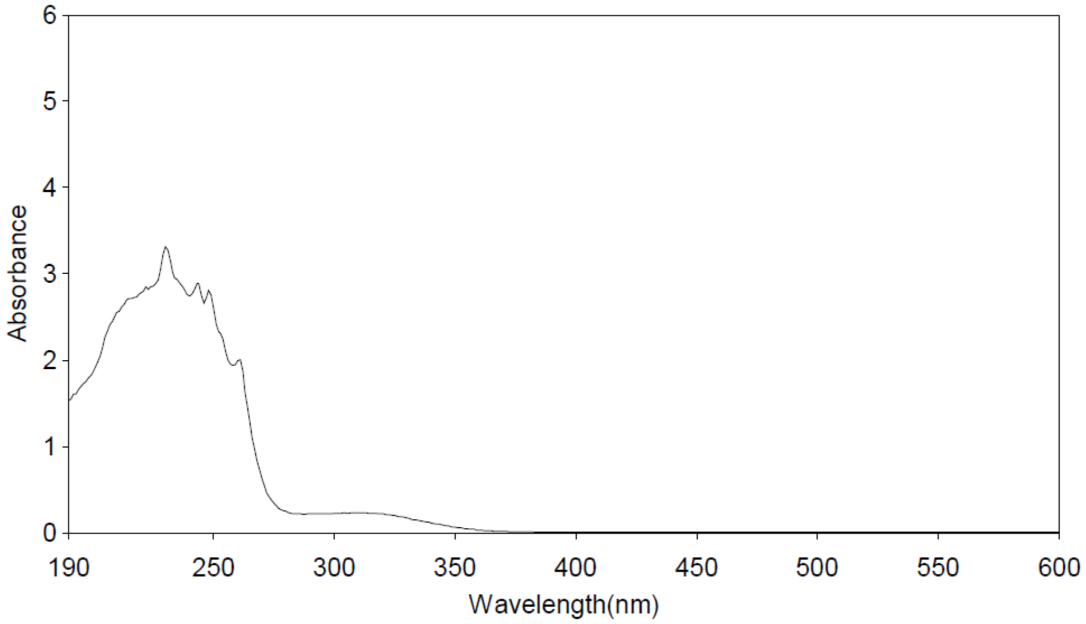

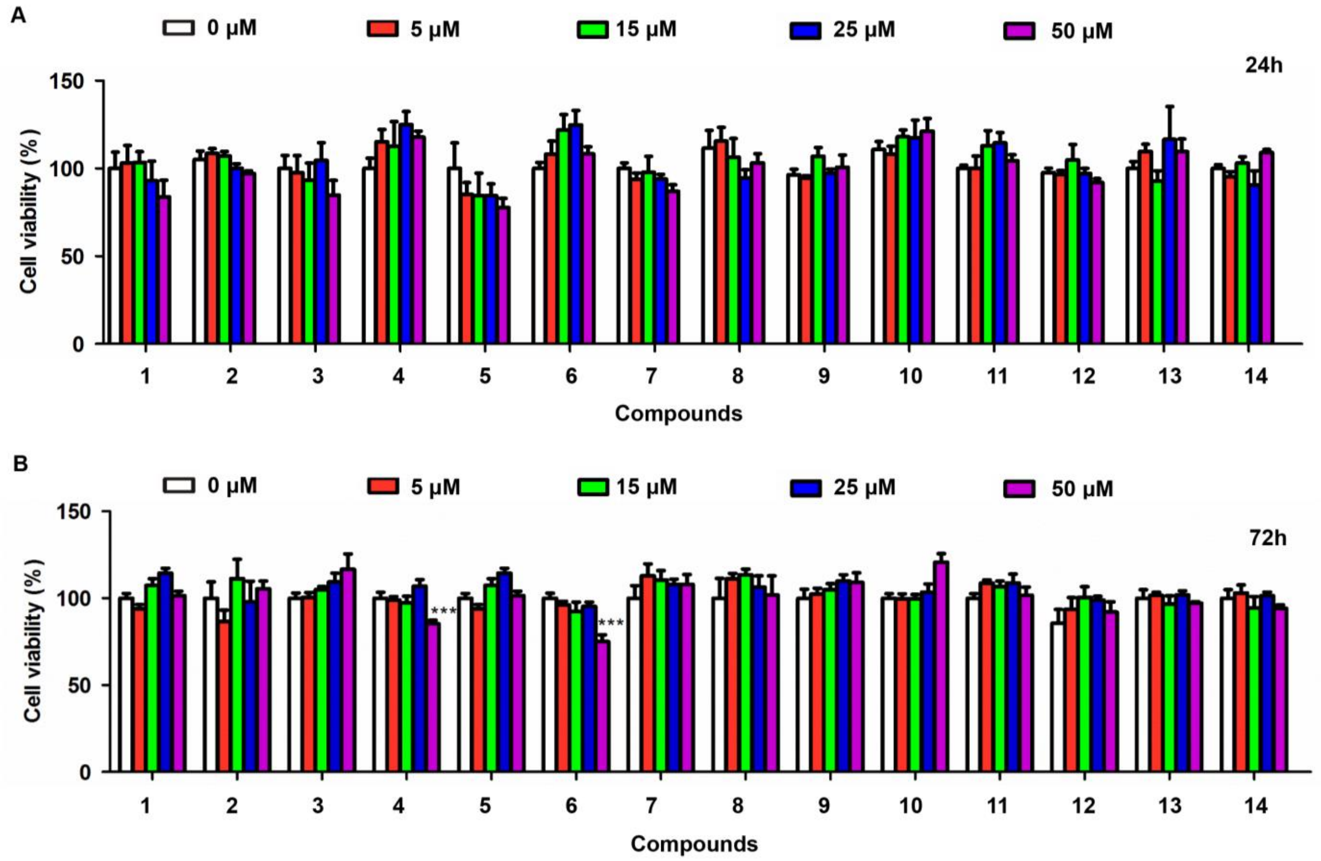

Figure S63. Cytotoxicity of compounds in BV2 microglia cells.

(A) Cell viability of BV2 cells following compounds treatment for 24 h. (B) Cell viability of BV2 cells following compounds treatment for 72 h. BV2 cells were seeded in 96-well plates and treated with different concentrations of compounds for 24 h or 72 h. Then the cell viability was measured by MTT assay. Drug treatment vs Control,***, $p < 0.001$.
